# Reliable reference miRNAs for quantitative gene expression analysis of stress responses in *Caenorhabditis elegans*

**DOI:** 10.1186/1471-2164-15-222

**Published:** 2014-03-21

**Authors:** Konstantinos Kagias, Agnieszka Podolska, Roger Pocock

**Affiliations:** 1 Biotech Research and Innovation Centre, University of Copenhagen, Ole Maaløes Vej 5, Copenhagen, Denmark

**Keywords:** Reference genes, miRNA, qPCR analysis, Normalization

## Abstract

**Background:**

Quantitative real-time PCR (qPCR) has become the “gold standard” for measuring expression levels of individual miRNAs. However, little is known about the validity of reference miRNAs, the improper use of which can result in misleading interpretation of data.

**Results:**

Here we undertook a systematic approach to identify highly stable miRNAs in different stress conditions such as low oxygen (hypoxia), UV-stress and high temperature (heat-stress) in the nematode *Caenorhabditis elegans*. We conducted genome-wide RNA-seq for small RNAs and selected abundant miRNAs with minimal variation of expression between the different conditions. We further validated the stable expression of a selection of those constitutively expressed candidates in the different stress conditions by SYBR Green qPCR. The selected miRNA candidates were analyzed for stability by applying the widely used geNorm logarithm. With this approach, we were able to successfully identify suitable reference miRNAs for each stress condition. Interestingly, we also found that 3 miRNAs, namely *mir-2-5p*, *mir-46-3p* and *mir-47-3p*, are stable in all the above-mentioned conditions suggesting that they might have general functions independent of stress.

**Conclusions:**

Our analysis offers a comprehensive list of stably expressed miRNAs in different stress conditions that can be confidently used as reference miRNAs for qPCR analysis in *C. elegans*.

## Background

The level at which molecules are expressed in a biological sample, under different conditions, often holds information about their mechanistic role(s). Quantitative real-time PCR (qPCR) has been established as one of the standard methods for semi-quantitative measurements of the abundance of various transcripts in virtually any biological sample. However, qPCR has been the source of many misleading reports due to being prone to experimental errors [1]. One of the most commonly encountered mistakes is the choice of unsuitable reference genes, which can lead to erroneous conclusions [2]. Reference genes are transcripts against which qPCR results are normalized. Currently, a number of normalization strategies for qPCR results exist, however, the use of reference genes is the most accepted one [3]. Reference genes have to exhibit stable expression between the conditions of interest in order to serve as suitable normalizers.

*Caenorhabditis elegans* has been widely used as an experimental model for a range or studies, spanning from development to drug discovery. Gene expression analysis in this model organism constitutes a powerful tool to discover new roles for different types of molecules, such as miRNAs. miRNAs are small RNA molecules (~22 nt) that mostly impact on gene expression post-transcriptionally [4] and have been implicated in different stress responses [5,6]. qPCR has extensively been used to assess expression levels of both protein coding genes and miRNAs in *C. elegans*. A previous study reported the validation and selection of appropriate reference genes for qPCR for protein coding genes [7], however, there is no report of stable miRNAs that can be used as reference genes in qPCR analysis of *C. elegans* miRNAs.

miRNA data normalization has traditionally relied on expression of other small RNAs, such as small nuclear (snRNAs) or small nucleolar (snoRNAs) RNAs (e.g. U8, U6 or RNU44). Using such transcripts is not ideal because these small RNAs are of different size and function, and are produced and/or processed by different pathways to miRNAs. Moreover, the use of only one or few popular reference genes in each and every qPCR experiment without experimental validation of the stable expression of these genes between the conditions used, can lead to heavily distorted results and misinterpretations.

**Figure 1:**
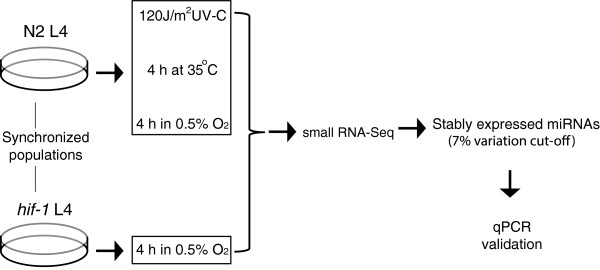
**Overview of the experimental pipeline.** Staged mid-L4 populations were subjected to the different stresses. Worm samples were collected after 4 h.

Here we undertook a systematic approach to identify stable miRNAs across a range of conditions (Figure 1). We chose to focus on three different, widely-studied, stress conditions in *C. elegans,* namely low oxygen (hypoxia), UV-stress and high temperature (heat-stress). We provide a selection of stably expressed and reliable reference genes for normalizing qPCR results obtained from stress studies in *C. elegans*.

## Results

### A battery of miRNAs is stably expressed throughout different stress conditions

**Figure 2:**
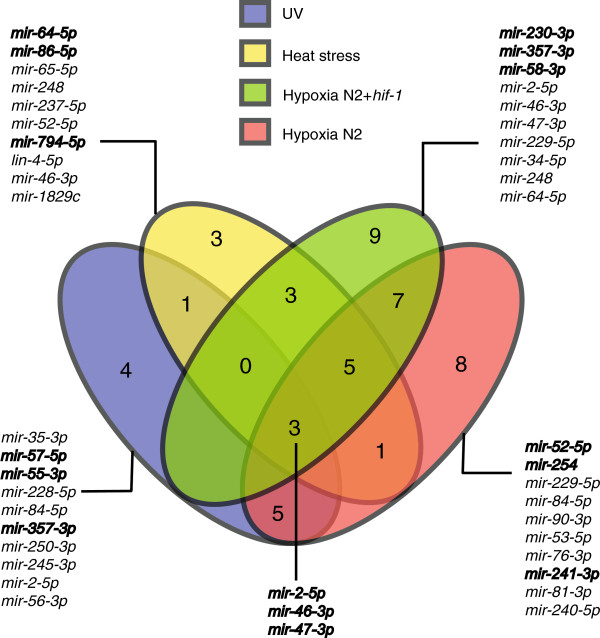
**Venn diagram of stably expressed miRNAs in the different conditions.** The 10 miRNAs with the least variation of expression are listed for each stress group as well as the three miRNAs that were found stable in all the conditions. Candidates chosen for further validation are indicated in bold.

**Figure 3:**
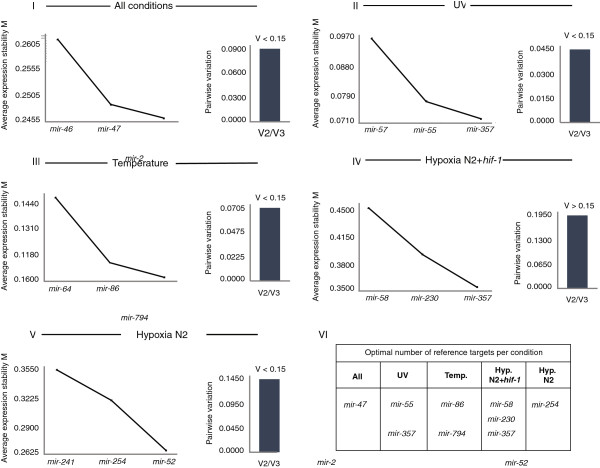
**Average expression stability (M) of remaining reference genes during stepwise exclusion of the least stable reference gene **[9]** and pairwise variation (V2/V3) of validated miRNAs. I**. *mir-46-3p*, *mir-47-3p* and *mir-2-5p* are stable in all conditions tested, **II**. *mir-57-5p*, *mir-55-3p* and *mir357-3p* are stable upon UV treatment, **III**. *mir-64-5p*, *mir-86-5p* and *mir-794-5p* are stable upon heat shock, **IV**. *mir-58-3p*, *mir-230-3p* and *mir-357-3p* are stable upon hypoxia regardless of HIF-1 presence, **V**. *mir-241-3p*, *mir-254* and *mir-52-5p* are stable upon hypoxia. **VI**. Table showing the optimal number of reference miRNAs suggested by V(V2/V3) analysis.

In an effort to identify miRNAs that are stably expressed in different stress conditions, we conducted genome-wide small RNA-seq on samples treated with hypoxia, UV-irradiation or high temperature, three widely studied stress conditions in *C. elegans* (Figure 1 and Additional file 1: Table S1). We then filtered the results for candidates that showed minimal variation of expression between unstressed conditions and each stress condition. We arbitrarily set a threshold of accepted expression variation to 7%, based on normalized number of reads for each miRNA in each sample. Furthermore, we discarded miRNAs with less than 500 reads as lowly expressed (Additional file 2: Table S2). We found 17 stably expressed miRNAs for UV-treated samples, 16 for the high temperature-treated samples, 32 for the hypoxia-treated samples and 28 for the hypoxia-treated samples, including samples of *hif-1* mutant animals (Figure 2, Additional file 3: Table S3). The *hif-1* mutant is a highly relevant strain for studying hypoxic responses and including *hif-1* samples in the study aimed to reveal *hif-1*-independent, stably expressed miRNAs. Notably, we found *mir-2-5p*, *mir-46-3p* and *mir-47-3p* to be stably expressed in all conditions (Figures 2 and 3 and Additional file 4: Figure S1, Additional file 5: Figure S2, Additional file 6: Figure S3). Thus, the high-throughput RNA-seq study provided a number of miRNAs exhibiting stable expression in each of the three stresses when compared to control conditions and three potential ‘general stress’ miRNA reference genes. These highly stable miRNAs may be involved in the regulation of basic cellular functions that are not influenced by any of the stresses used, thus their expression remains unchanged.

### Validation of stable expression of selected miRNAs by an inexpensive, selective and sensitive qPCR method and subsequent geNorm analysis

The use of three different reference genes has recently been shown to be sufficient for accurate normalization of results obtained by qPCR analysis for protein-coding genes in *C. elegans*[7,8], as was previously suggested [9]. For this reason we decided to validate the stable expression of three miRNAs in each stress condition, choosing candidates that span over a wide range of abundance (low, medium and high levels), as well as to validate the three miRNAs that are stable between all the conditions. We chose to use an optimized method for miRNA qPCR that is based on a single reverse transcription reaction for all miRNAs followed by a real-time qPCR reaction with a pair of DNA based miRNA specific primers [10]. This technique is highly sensitive and can recognize between miRNAs that differ by as little as one nucleotide [10]. Moreover, this technique is fairly inexpensive. We believe that the identified candidates would also be appropriate reference genes to be used with other qPCR chemistries, such as TaqMan or SYBR Green with LNA primers.

### miRNAs selected for validation of stable expression for each sample group

**Figure 4:**
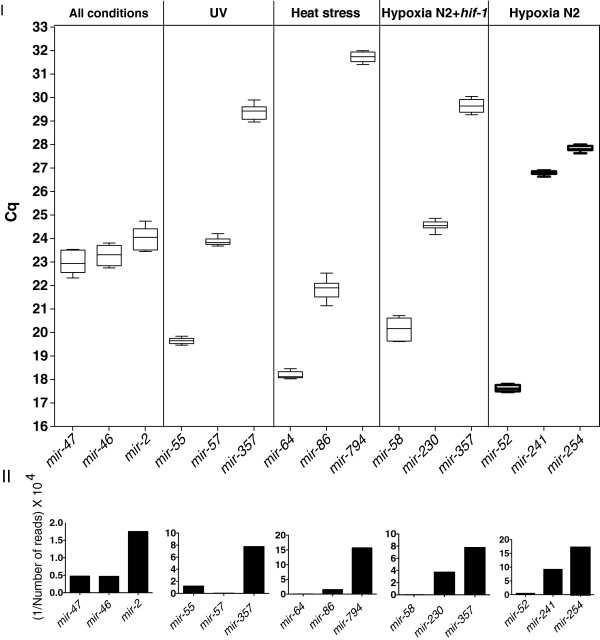
**Abundance of validated miRNAs as indicated by the Cq value of qPCR reactions and RNA-seq read counts. I**. Graphical representation of the Cq values obtained by qPCR in the control sample. Each box represents the range of Cq values of all the qPCR reactions performed for each miRNA. The horizontal lines in the boxes indicate the median Cq. **II**. Graphical representation of the RNA-seq read results for each miRNA. In order for the graphs to be in line with the data presentation on the panel I and therefore easily comparable, the number of reads was calculated as a fraction of 1 and the outcome was multiplied by a large number (10^4^) to give a high value. The bigger the number, the smaller the abundance of the miRNA, similar to Cq function.

**UV**: For the UV-treated samples, we chose ***mir-57-5p*** as highly expressed (215390,119 reads), ***mir-55-3p*** as intermediately expressed (8240,595 reads) and ***mir-357-3p*** as lowly expressed (1289,386 reads) to validate by qPCR (Figure 4).

**Heat**-**stress**: For the high temperature-treated samples, we selected ***mir-64-5p*** as highly expressed (163324,299 reads), ***mir-86-5p*** as intermediately expressed (6457,365 reads) and ***mir-794-5p*** as lowly expressed (636,433 reads) to validate by qPCR (Figure 4).

**Hypoxia N2**: For the hypoxia-treated samples, we selected ***mir-52-5p*** as highly expressed (19899,848 reads), ***mir-241-3p*** as intermediately expressed (1086,805 reads) and ***mir-254*** as lowly expressed (585,136 reads) to validate by qPCR (Figure 4).

**Hypoxia N2 +** ***hif-1***: For the hypoxia-treated samples including the *hif-1* mutant, we chose ***mir-58-3p*** as highly expressed (375488,779 reads), ***mir-230-3p*** as intermediately expressed (2681,376 reads) and ***mir-357-3p*** as lowly expressed (1287,386 reads) to validate by qPCR (Figure 4).

**All conditions**: ***mir-47-3p*** (21043,167 reads), ***mir-46-3p*** (22186,815 reads) and ***mir-2-5p*** (5977,944 reads) were stably expressed in all the conditions and therefore chosen to validate by qPCR (Figure 4).

The detected Cq (Quantification cycle) values for all but one (*mir-57-5p*) of the above miRNAs correlated with the read counts obtained by RNA-seq. *mir-57-5p* is found to be more abundant than *mir-55-3p* in UV-treated samples when tested by qPCR, probably due to RNA-seq inaccuracy (Figure 4).

### geNorm analysis results

The relative expression levels of all the selected miRNAs were calculated based on the Cq values obtained, with the primer efficiencies taken into account. Subsequently, the geNorm logarithm was used to calculate the average gene expression stability (M value) of each of the miRNAs. The more stable the expression of a gene is, the lower is its M value. All the miRNAs tested in our study showed an M value less than 0.5 (excluding *mir-58-3p* - M value equals 0.573), which is the suggested cut-off of geNorm corresponding to high expression stability. The coefficience of variation for all of the candidates was < 0.2. Based on M values we conclude that the selected miRNAs are stably expressed between the conditions tested and can confidently be used as reference genes in qPCR analysis in *C. elegans,* when the particular stress conditions are under study. The order for each group of miRNAs based on M value is shown in Figure 3.

Next, we wanted to determine the optimal number of reference genes to be used in qPCR studies of stress responses, as assessed by the pairwise variation value (Vn/n + 1 value). The V2/3 value for all but ‘Hypoxia N2 + *hif-1*′ miRNA groups was <0.15, which indicates that the use of the two best miRNAs, as previously evaluated by M value, is sufficient for accurate normalization (Figure 3). In ‘Hypoxia N2 + *hif-1*′ group the V2/3 value was >0.15 (0.189) and therefore qBase suggests the use of all 3 miRNAs (Figure 3).

### Primer efficiency and specificity evaluation

All the primer pairs used for the qPCR reactions (Table 1) were highly efficient as calculated by the use of sequential dilutions of a representative cDNA sample (Table 2). Moreover, the specificity of the primers was assessed by standard melting curve analysis (Additional file 7: Figure S4). All primer pairs gave a single melting peak indicating their specificity in amplifying only one product. ‘No reverse transcription’ controls as well as ‘no template controls’ showed no amplification suggesting no DNA contamination in the samples and no contamination in the reagents used for qPCR experiment, respectively. Moreover, the RNA used was of excellent quality as assessed by Nanodrop and Bioanalyzer, a crucial prerequisite for reliable gene expression estimation (Additional file 8: Figure S5).

**Table 1:** Primers used to perform qPCR of selected candidates

**All conditions**			
**miRNA**	**Sequence**	**Forward primer**	**Reverse primer**
*mir-2-5p*	UAUCACAGCCAGCUUUGAUGUGC	TCACAGCCAGCTTTGATG	GTCCAGTTTTTTTTTTTTTTTGCAC
*mir-46-3p*	UGUCAUGGAGUCGCUCUCUUCA	TGTCATGGAGTCGCTCTC	GGTCCAGTTTTTTTTTTTTTTTGAAGA
*mir-47-3p*	UGUCAUGGAGGCGCUCUCUUCA	CATGGAGGCGCTCTC	GGTCCAGTTTTTTTTTTTTTTTGAAGA
**UV**			
*mir-357-3p*	AAAUGCCAGUCGUUGCAGGAGU	TGCCAGTCGTTGCAG	GGTCCAGTTTTTTTTTTTTTTTACTC
*miR-57-5p*	UACCCUGUAGAUCGAGCUGUGUGU	AGTACCCGTATAAGTTTCTGCT	GGTCCAGTTTTTTTTTTTTTTTCTC
*miR-55-3p*	UACCCGUAUAAGUUUCUGCUGAG	GCAGTATGACACTGAAGCGT	GTCCAGTTTTTTTTTTTTTTTCGGT
**Heat-stress**			
**miRNA**	**Sequence**	**Forward primer**	**Reverse primer**
*miR-64-5p*	UAUGACACUGAAGCGUUACCGAA	GCAGTATGACACTGAAGCGT	GTCCAGTTTTTTTTTTTTTTTCGGT
*miR-86-5p*	UAAGUGAAUGCUUUGCCACAGUC	AGTAAGTGAATGCTTTGCCA	GTCCAGTTTTTTTTTTTTTTTGAC
*miR-794-5p*	UGAGGUAAUCAUCGUUGUCACU	CAGTGAGGTAATCATCGTTGT	GTCCAGTTTTTTTTTTTTTTTAGTGA
**Hypoxia N2 +** ***hif-1***			
**miRNA**	**Sequence**	**Forward primer**	**Reverse primer**
*miR-230-3p*	GUAUUAGUUGUGCGACCAGGAGA	GGTATTAGTTGTGCGACCAG	GGTCCAGTTTTTTTTTTTTTTTCTC
*miR-357-3p*	AAAUGCCAGUCGUUGCAGGAGU	TGCCAGTCGTTGCAG	GGTCCAGTTTTTTTTTTTTTTTACTC
*miR-58-3p*	UGAGAUCGUUCAGUACGGCAAU	CAGTGAGATCGTTCAGTACG	GGTCCAGTTTTTTTTTTTTTTTATTGC
**Hypoxia N2**			
**miRNA**	**Sequence**	**Forward primer**	**Reverse primer**
*miR-52-5p*	CACCCGUACAUAUGUUUCCGUGCU	ACCCGTACATATGTTTCCGT	GGTCCAGTTTTTTTTTTTTTTTAGCA
*miR-254*	UGCAAAUCUUUCGCGACUGUAGG	GCAAATCTTTCGCGACTGT	GGTCCAGTTTTTTTTTTTTTTTCCT
*miR-241-3p*	AUUGUCUCUCAGCUGCUUCAUC	AGATTGTCTCTCAGCTGCT	GTCCAGTTTTTTTTTTTTTTTGATGAAG

**Table 2:** Amplification efficiency of primers used

**All conditions**		
**miRNA**	**Efficiency (%)**	**R square**
*mir-2-5p*	119.6	0.980
*mir-46-3p*	107.2	0.926
*mir-47-3p*	102.6	0.918
**UV**		
**miRNA**	**Efficiency (%)**	**R square**
*mir-357-3p*	111.7	0.924
*miR-57-5p*	106.5	0.910
*miR-55-3p*	112.2	0.965
**Heat**-**stress**		
**miRNA**	**Efficiency (%)**	**R square**
*miR-64-5p*	104.2	0.918
*miR-86-5p*	117.9	0.904
*miR-794-5p*	92.8	0.921
**Hypoxia N2 +** ***hif-1***		
**miRNA**	**Efficiency (%)**	**R square**
*miR-230-3p*	106.5	0.84
*miR-357-3p*	111.7	0.924
*miR-58-3p*	89	0.902
**Hypoxia N2**		
**miRNA**	**Efficiency (%)**	**R square**
*miR-52-5p*	115.6	0.873
*miR-254*	96.9	0.908
*miR-241-3p*	95.6	0.962

## Discussion

In recent years, significant effort has been directed towards re-evaluating the way that qPCR experiments are conducted [2], with much focus on the identification of suitable reference genes in different experimental conditions. In this study, we used genome-wide RNA-seq for small RNAs as an unbiased approach to discover stably expressed miRNAs in a range of different stress conditions. This approach allowed us to identify a number of miRNAs with stable expression levels ranging from 500 to many thousands of reads (Additional file 2: Table S2). We further evaluated a subset of these miRNAs by applying the commonly used geNorm logarithm on qPCR measurements. All the miRNAs selected for further evaluation showed high expression stability. This strong consistency between RNA-seq results and qPCR analysis suggests that the stable miRNAs found by RNA-seq could all be potentially used as reference genes in qPCR analysis. In fact, a previous sequencing study has shown that two of the miRNAs identified in our study, *mir-46-3p* and *mir-47-3p*, are remarkably stable when expression levels are compared between young adults and aged animals [11]. However, we strongly advise the prospective user to perform qPCR validation in each experimental setup and test the stability of chosen miRNAs using geNorm or other similar analysis tool (e.g. NormFinder [12], BestKeeper [13]), before confidently using them as reference genes. Nevertheless, we have validated the stable expression of a sufficient number of miRNAs for each condition, which the user can confidently use in qPCR analysis.

The method of choice for performing the qPCR was described by Balcells et al. [10]. This method has key advantages; it is simple (single RT-reaction) and inexpensive (regular DNA primers) with high sensitivity and specificity. Although we used this method to validate stable miRNAs, we believe that the identified candidates would be appropriate reference genes to be used with other qPCR chemistries, such as TaqMan or qPCR with LNA primers.

## Conclusions

Our study provides a comprehensive list of miRNAs that can confidently be used for normalization of qPCR data in *C. elegans* stress biology. Moreover, it demonstrates an unbiased approach to identify and validate appropriate reference miRNAs based on their stable expression in genome-wide RNA seq data.

## Methods

### Worm maintenance and worm sample preparation

Mixed populations of worms were grown on *E. coli* (strain OP50) seeded NGM (Nematode-Growth-Medium) agar plates for 2–3 days at 20°C as previously described [14]. Worms were washed off the plates and bleached to obtain eggs. These eggs were kept in sterile M9 buffer (3 g KH_2_PO_4_, 6 g Na_2_HPO_4_, 5 g NaCl, 1 ml 1 M MgSO_4_, H_2_O to 1 liter) overnight to hatch and developmentally arrest at the L1 stage. Starvation staged L1 worm populations were grown on OP50 seeded NGM plates until the L4 stage. For the hypoxia-treated samples, mid-L4 animals were placed in a hypoxic chamber with 0.5% O_2_ for 4 h and harvested immediately. For the heat-stress-treated samples, mid-L4 animals were heated at 35°C for 4 h and harvested immediately. For the UV-treated samples, mid-L4 animals were irradiated with 120 J/m^2^ UV-C and harvested 4 h post-treatment. Samples were harvested by washing with M9. One set of samples was used for the small RNA-seq and three biologically independent sets of samples for the qPCR validation.

### RNA isolation

Worm pellets were kept in Trizol and lysed by freeze-cracking. A round of chloroform phase separation was followed by an addition of 1.5 volumes of 100% ethanol. Consecutive miRNeasy column purification was performed as per supplier instructions starting from step nine in the miRNeasy guidebook (Qiagen) [15]. RNA concentration and purity was assessed by Nanodrop (Thermo Scientific) and followed by RNA integrity assessment using Bioanalyzer RNA Nano Chips (Agilent Technologies). The RIN (RNA Integrity Number) of the total RNA samples ranged from 10.0-7.1 with 10 being the highest possible value (Additional file 8: Figure S5).

### Genome-wide small RNA-seq

The preparation of the RNA samples for small RNA-seq and the analysis of sequencing data was performed as described in Friedlander *et al.*[16] and is detailed below.

### Small RNA library preparation for sequencing

Small RNA library preparation was obtained from 1 μg of total RNA per sample. 5′-monophosphate dependent small RNA cloning and library preparation was then performed with the TruSeq Small RNA Sample Preparation Kit (Illumina, USA). Cluster generation as well as sequencing of the prepared libraries was performed on the Illumina cluster station and GAIIx (Illumina, USA).

### miRNA fold-change analysis

Preprocessing of raw sequencing data was performed with a custom PERL script to group reads with different barcodes into separate fasta files. These fasta files were processed with the mapper.pl module from miRDeep2 [16] to clip sequencing adapters, collapse reads and map them to the *C. elegans* genome (WS205) with the following command: mapper.pl config.txt -c -d -i -j -k TGGAATTCTCGGGTGCCAAGG -l 18 -m -p c_elegans.WS205.dna -s reads.fa -t reads_vs_genome.arf -v. Raw read counts as output by the quantifier module from the miRDeep2 package were normalized to the total number of miRNA reads per barcode then multiplied by the mean value of all miRNA reads of all barcode files. A pseudocount of 10 was added and the normalized values were log2 transformed. A regression line with slope 1 was calculated in R for all pairwise sample comparisons and the deviation from each data point to this line (residual) was used to estimate the fold change between two samples.

### Primer design

qPCR DNA primers were designed according to previously published instructions [10] using a custom-made primer design software that can be downloaded from Aalborg University server:

“http://vbn.aau.dk/en/publications/mirprimerdesign3(bcd08649-3883-4016-b493-52dceedd04f8).html.” (Courtesy of Peter Kamp Busk, University of Aalborg, Denmark). A list of the primers used can be found in the Table 1.

### cDNA synthesis and quantitative real-time PCR

cDNA synthesis was essentially performed as previously described [10]. Briefly, single tube poly (A) synthesis and reverse transcription was performed with 100 ng of RNA in a final volume of 10 μl combined with 1 μl MuLV reverse transcriptase (New England Biolabs), 1 μl of poly (A) polymerase (New England Biolabs), 1 μl 10x poly (A) polymerase buffer, 1 μl 0.1 mM ATP, 1 μl 10 μM RT-primer (5′-CAGGTCCAGTTTTTTTTTTTTTTTVN where V is A, C and G and N is A, C, G and T). The reaction was incubated at 42°C for 1 hour followed by enzyme inactivation at 95°C for 5 minutes. A panel of three technical cDNA replicates for each of the three biological samples per condition was constructed. The cDNA was diluted 5× before used in qPCR reactions.

miR-specific qPCR was performed as previously described in [16]. Briefly, 0.25 μl of 10 μM miRNA specific forward and 0.25 μl of 10 μM miRNA specific reverse primer, 5 μl of 2× LightCycler 480 SYBRGreen I Mastermix (Roche) and 3.5 μl of RNAse free H_2_O and 1 μl of cDNA of each samples was combined in 10 μl reaction and subjected to qPCR performed on LightCycler 480 (Roche).

### Data analysis

Sequential dilutions of a representative cDNA sample were made in order to calculate the primer pair efficiency (E) using the formula E% = (10^(1/slope)^-1) × 100. The Cq values obtained from the qPCR runs were applied into the geNorm algorithm [9] as part of the qBase software package [17] to calculate the expression stability value (M value) and the pairwise variation Vn/n + 1. The M value is defined as the average pairwise variation of a single candidate reference gene to all other tested candidates. The commonly accepted cut off of 0.15 for the pairwise variation was used to assess the minimum number of reference genes required for accurate normalization [9].

## Competing interests

The authors declare no competing financial or non-financial interests.

## Authors’ contributions

Conceived, designed and performed the experiments: AP and KK. Analyzed the data: KK and AP. Wrote the paper: KK, AP and RP. All authors read and approved the final manuscript.

## Supplementary Material

Additional file 1: Table S1 Total number of uniquely mapped reads obtained from RNA sequencing. Click here for file

Additional file 2: Table S2 Results of the small RNA-seq listed according to their variability between the different samples. A 7% cut-off for variation was chosen. Click here for file

Additional file 3: Table S3 miRNAs included in Venn’s diagram of Figure 1. Click here for file

Additional file 4: Figure S1 Graphical output files from mirDeep2 showing the reads, counts per read and mapping on the hairpin for *mir-2*, *mir-46* and *mir-47*. Click here for file

Additional file 5: Figure S2 Graphical output files from mirDeep2 showing the reads, counts per read and mapping on the hairpin for *mir-2*, *mir-46* and *mir-47*. Click here for file

Additional file 6: Figure S3 Graphical output files from mirDeep2 showing the reads, counts per read and mapping on the hairpin for *mir-2*, *mir-46* and *mir-47*. Click here for file

Additional file 7: Figure S4 Melting curves (smaller graphs on the top) and melting peaks (bigger graphs at the bottom) of the primers used to validate selected miRNAs by qPCR. All pairs of primers show harmonious curves and single peaks, which indicate specificity in amplifying one product only. Click here for file

Additional file 8: Figure S5 RNA quality and integrity assessed by Nanodrop and Bioanalyzer. Bioanalyzer image for all samples (numbering of the samples as in b) where high integrity of the RNA samples is observed. bp = base pairs. b. 260/280 and 260/230 ratios obtained by the Nanodrop as well as RIN (RNA Integrity Number) measurements obtained by the Bioanalyzer for all the samples. Click here for file
