# Supplementary material for: Reliable reference miRNAs for quantitative gene expression analysis of stress responses in Caenorhabditis elegans
Source: BMC Genomics. 2014 Mar 21;15:222. doi: 10.1186/1471-2164-15-222 (PMC3997968; doi:10.1186/1471-2164-15-222)
Supplement: Additional file 4: Figure S1 — Graphical output files from mirDeep2 showing the reads, counts per read and mapping on the hairpin for mir-2, mir-46 and mir-47. [file 1471-2164-15-222-S4.pdf]

miRBase precursor : cel-mir-2
Total read count : 40667
cel-miR-2 read count : 40237
cel-miR-2* read count : 412
remaining reads : 18

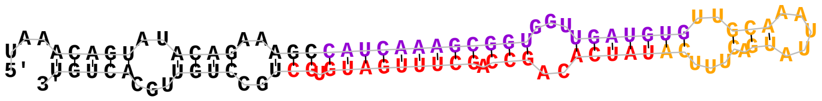

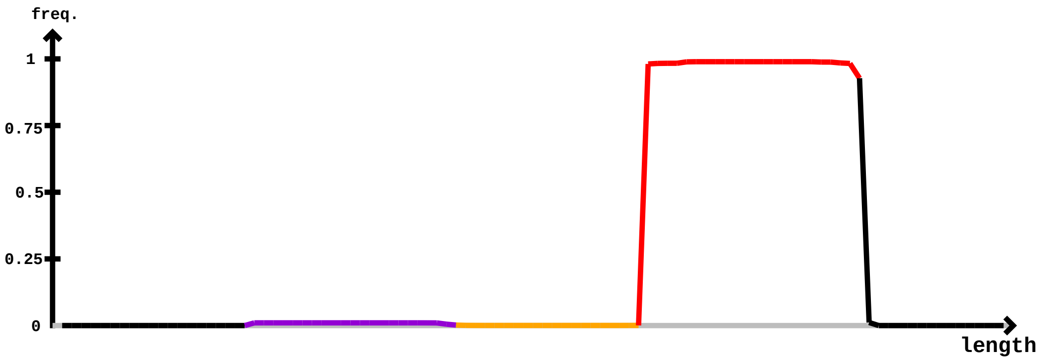


```
                                                                          cel-miR-2
                                   cel-miR-2*
5'- uaaacaguauacagaaagccaucaaagcggugguugaugugguugcaaauuaugacuuucauaucacagccagcuuugaugugcugccuguugcacugu   -3'    exp
    ...(((((..((((..(((((((((((((((...(((((((..(((.....)).)...)))))))..))).))))))))).)))..))))...)))))   reads     mm       sample
    ...................caucaaagcggugguugau............................................................   1         0        bc4
    ...................caucaaagcggugguuUaug...........................................................   1         1        bc4
    ...................caucaaagcggugguugaug...........................................................   16        0        bc4
    ...................caucaaagcggugguugaugu..........................................................   6         0        bc4
    ...................caucaaagcggugguugaugug.........................................................   8         0        bc4
    .........................................uugcaaauuaugacuuuca......................................   1         0        bc4
    ............................................................uaucacagccagcuuugaug..................   7         0        bc4
    ............................................................uaucacagccagcuuugaugu.................   5         0        bc4
    ............................................................uaucacagccagcuuugaugug................   106       0        bc4
    ............................................................uaucacaUccagcuuugaugug................   1         1        bc4
    ............................................................uaucacagccagcuuugauguU................   4         1        bc4
    ............................................................uaucaUagccagcuuugaugug................   1         1        bc4
    ............................................................uaucacagccagcuuugauguA................   1         1        bc4
    ............................................................uauUacagccagcuuugaugugc...............   1         1        bc4
    ............................................................uaucacagccagcuuugaugGgc...............   5         1        bc4
    ............................................................uaucGcagccagcuuugaugugc...............   1         1        bc4
    ............................................................uaucacagccagcuuuUaugugc...............   2         1        bc4
    ............................................................uaucacagccagcuuugaugugG...............   11        1        bc4
    ............................................................uaucCcagccagcuuugaugugc...............   2         1        bc4
    ............................................................uaucacagccagcuuugaAgugc...............   1         1        bc4
    ............................................................uaucacagccGgcuuugaugugc...............   1         1        bc4
    ............................................................uaucacagccagGuuugaugugc...............   2         1        bc4
    ............................................................uaucacagccagcuuugaugCgc...............   1         1        bc4
    ............................................................uaucacagccagcuuugaGgugc...............   1         1        bc4
    ............................................................uaucaUagccagcuuugaugugc...............   1         1        bc4
    ............................................................uauAacagccagcuuugaugugc...............   1         1        bc4
    ............................................................uaucaAagccagcuuugaugugc...............   3         1        bc4
    ............................................................uaucacagccagcuuugauUugc...............   1         1        bc4
    ............................................................uaucacaUccagcuuugaugugc...............   1         1        bc4
    ............................................................uGucacagccagcuuugaugugc...............   1         1        bc4
    ............................................................uaucacagAcagcuuugaugugc...............   1         1        bc4
    ............................................................uaucacagccagcuuuAaugugc...............   1         1        bc4
    ............................................................uaucacagccUgcuuugaugugc...............   2         1        bc4
    ............................................................uaucacagccCgcuuugaugugc...............   2         1        bc4
```

cel-miR-2

cel-miR-2*

uaaacaguauacagaaagccaucaaagcggugguugauguguugcaaauuaugacuuucauaucacagccagcuuugaugugcugccuguugcacugu

....uaucacagccagcuuugaugugA...............	4	1	bc4
....uaucacagccagcuuugauguAc...............	1	1	bc4
....uaucacagccagcuuugaugugU...............	13	1	bc4
....uaucacagccagcuuugaugAgc...............	1	1	bc4
....uaucacagcGagcuuugaugugc...............	1	1	bc4
....Gaucacagccagcuuugaugugc...............	1	1	bc4
....uaucacUgccagcuuugaugugc...............	2	1	bc4
....uaucacagccagcuuugauguCc...............	1	1	bc4
....uaucacagccagcuuugaCgugc...............	1	1	bc4
....uaucacagccagcuuugaugugc...............	2111	0	bc4
....uaucacGgccagcuuugaugugc...............	2	1	bc4
....uauGacagccagcuuugaugugc...............	1	1	bc4
....uaucacagccagcuuugaugugcu..............	16	0	bc4
....uaucacagccagcuuugaugugUu..............	1	1	bc4
....uaucacagccagcuuugaugGgcu..............	1	1	bc4
....uaucacagAcagcuuugaugugcu..............	1	1	bc4
....aucacagccagcuuugaugugc...............	1	0	bc4
....aucacagccagcuuugaugugcu..............	1	0	bc4
....ucacagccagcuuugaugugc...............	1	0	bc4
....acagccagcuuugaugug................	1	0	bc4
....acagccagcuuugaugugc...............	12	0	bc4

...................caucaaagcggugguugaug...	11	0	bc1
...................caucaaagcggugguugaugu..	12	0	bc1
...................caucaaagcggugguugaugug...	2	0	bc1
...................caucaaagcggugguugaugugu..	1	0	bc1
....uugcaaauuaugacuuuca......................................	2	0	bc1
....uaucacagcAagcuuuga....................	1	1	bc1
....uaucacagccagcuuuga....................	4	0	bc1
....uaucacagccagcuuugaug..................	7	0	bc1
....uaucaUagccagcuuugaug..................	1	1	bc1
....uaucacagccagcuuugaugu.................	1	0	bc1
....uaucacagccagcuuugauguU................	5	1	bc1
....uaucacagccagcuuugauguA................	1	1	bc1
....uaucacagccagcuuugaugug................	110	0	bc1
....uaucacagccagcCuugaugug................	1	1	bc1
....uaucacagccagGuuugaugugc...............	1	1	bc1
....uaucacagcAagcuuugaugugc...............	3	1	bc1
....uaucacGgccagcuuugaugugc...............	2	1	bc1
....uaucacagccagcuuugauguCc...............	1	1	bc1
....uaucacagccagcuuugaugugU...............	6	1	bc1
....uaucaAagccagcuuugaugugc...............	1	1	bc1
....uaucacagccagcuuugaugugc...............	2689	0	bc1
....uaucacagccagcuuugUugugc...............	2	1	bc1
....uaucacagccagcuuugGugugc...............	1	1	bc1
....uaucacagccagcuuuUaugugc...............	2	1	bc1
....Caucacagccagcuuugaugugc...............	1	1	bc1
....uaucacCgccagcuuugaugugc...............	1	1	bc1
....uaucacagccagcuuugaugugG...............	6	1	bc1
....uaucacagccagcuuugaAgugc...............	2	1	bc1
....uaucacagcGagcuuugaugugc...............	2	1	bc1
....uaucacagccagcuuuCaugugc...............	1	1	bc1
....uaucacagccagcuuugCugugc...............	1	1	bc1
....uaucacagccagcuuugaugGgc...............	8	1	bc1
....uGucacagccagcuuugaugugc...............	1	1	bc1
....uaucacagccagcuuugauAugc...............	1	1	bc1
....uaucacUgccagcuuugaugugc...............	1	1	bc1
....uaucacagccagAuuugaugugc...............	2	1	bc1
....uaucacaUccagcuuugaugugc...............	2	1	bc1
....uauGacagccagcuuugaugugc...............	2	1	bc1
....uaucacagccagcuuugaGgugc...............	3	1	bc1
....uaucacagccagcuuugaugugA...............	2	1	bc1
....uaucacagccagcuuugaugCgc...............	1	1	bc1
....uaucacagccagcuuuAaugugc...............	2	1	bc1
....uaucacaCccagcuuugaugugc...............	1	1	bc1
....uaucacagccagcuuugauUugc...............	3	1	bc1
....uaucacagccagcuGugaugugcu..............	1	1	bc1
....uaucacagccagcuuugaugugcA..............	3	1	bc1
....uaucacagccagcuuugaugugcu..............	32	0	bc1

cel-miR-2

cel-miR-2*

uaaacaguauacagaaagccaucaaagcggugguugauguguugcaaauuaugacuuucauaucacagccagcuuugaugugcugccuguugcacugu

....uaucacagccagcuuugaugugcC..............	1	1	bc1
....aucacagccagcuuugaugugc...............	5	0	bc1
....aucacagccagcuuugaugGgc...............	1	1	bc1
....aucacagccagcuuugaugugcu..............	1	0	bc1
....ucacagccagcuuugaugugcu..............	1	0	bc1
....cacagccagcuuugaugugc...............	1	0	bc1
....acagccagcuuugaugugc...............	19	0	bc1
...................caucaaagcggugguugau..	1	0	bc3
...................caucaaagcggugguugaug...	20	0	bc3
...................caucaaagcggugguugaugu..	14	0	bc3
...................caucaaagcggugguugauUu..	1	1	bc3
...................caucaaagcggugguugaugug...	10	0	bc3
...................caucaaagcggugguugauguU...	1	1	bc3
...................caucaaagcggugguugaugugu..	2	0	bc3
...................caucaaagcggugguugauguguugcaaauuaugacuuucau.....................................	1	0	bc3
....uugcaaauuaugacuuuca......................................	2	0	bc3
....uugcaaauuaugCcuuuca......................................	1	1	bc3
....Cuaucacagccagcuuugaugugc...............	1	1	bc3
....uaucacagccagcuuuga....................	8	0	bc3
....uaucacagccagcuuugau...................	1	0	bc3
....uaucacagccagcuuugaug..................	14	0	bc3
....uGucacagccagcuuugaugu.................	1	1	bc3
....uaucacagccagcuuugaugu.................	12	0	bc3
....uaucacagccGgcuuugaugug................	1	1	bc3
....uaucacagccagcuuugauguC................	3	1	bc3
....uaucacagccagcuuuUaugug................	1	1	bc3
....uaucacagccagcuuugaugug................	278	0	bc3
....uaucacagccagcuuugauguU................	12	1	bc3
....uaucacagccagcuuugGugug................	1	1	bc3
....uaucacagccagcuuugauAug................	2	1	bc3
....uaucacagUcagcuuugaugugc...............	1	1	bc3
....uaucacagccaUcuuugaugugc...............	1	1	bc3
....uaucacagcGagcuuugaugugc...............	1	1	bc3
....uaucacagccagcuuugauguUc...............	1	1	bc3
....uaucacagccagGuuugaugugc...............	1	1	bc3
....uaucacagccUgcuuugaugugc...............	1	1	bc3
....uaucacaUccagcuuugaugugc...............	3	1	bc3
....uaucacagccagcuuugauCugc...............	3	1	bc3
....uaucacagccagcuuugaugugA...............	8	1	bc3
....uaucCcagccagcuuugaugugc...............	1	1	bc3
....uaucacagccagUuuugaugugc...............	2	1	bc3
....uaCcacagccagcuuugaugugc...............	2	1	bc3
....uaucacagcUagcuuugaugugc...............	1	1	bc3
....uaucacagccagcuuugaugugc...............	5428	0	bc3
....uaucacagccagcuuugaGgugc...............	1	1	bc3
....uaucacagccCgcuuugaugugc...............	2	1	bc3
....uaucacCgccagcuuugaugugc...............	1	1	bc3
....uaucacagccagAuuugaugugc...............	1	1	bc3
....uaucacagccagcuuAgaugugc...............	1	1	bc3
....uaucacagccagcuuuUaugugc...............	3	1	bc3
....uaucacagccagcuuugauguAc...............	4	1	bc3
....uaucacagccagcuuCgaugugc...............	1	1	bc3
....uaucacagccagcuuugaugugU...............	14	1	bc3
....uaucacagccagcuuugaCgugc...............	1	1	bc3
....uauAacagccagcuuugaugugc...............	1	1	bc3
....uaucGcagccagcuuugaugugc...............	2	1	bc3
....uaucacagccagcuuugauAugc...............	3	1	bc3
....uaucacagccGgcuuugaugugc...............	3	1	bc3
....uaucacagccagcuuugGugugc...............	1	1	bc3
....uauGacagccagcuuugaugugc...............	1	1	bc3
....uaucacagccagcuuugaugGgc...............	17	1	bc3
....uauUacagccagcuuugaugugc...............	1	1	bc3
....uaucacagccagcuuugaugCgc...............	5	1	bc3
....uaucaGagccagcuuugaugugc...............	1	1	bc3
....uaucacagAcagcuuugaugugc...............	1	1	bc3
....uaucacagccagcAuugaugugc...............	1	1	bc3
....uaucacagccaCcuuugaugugc...............	2	1	bc3
....Aaucacagccagcuuugaugugc...............	1	1	bc3

cel-miR-2

cel-miR-2*

uaaacaguauacagaaagccaucaaagcggugguugauguguugcaaauuaugacuuucauaucacagccagcuuugaugugcugccuguugcacugu

....uaucacagccagcCuugaugugc...............	7	1	bc3
....uaucacagccagcuuugaugugG...............	9	1	bc3
....uaucacGgccagcuuugaugugc...............	2	1	bc3
....uaucaUagccagcuuugaugugc...............	1	1	bc3
....uaucacagccagcuuugauUugc...............	1	1	bc3
....uaucacagccagcuuugaugugcA..............	13	1	bc3
....uaAcacagccagcuuugaugugcu..............	1	1	bc3
....uaucacagccagcuuugaugugcu..............	51	0	bc3
....uaucacagccagcuuugaugugUu..............	1	1	bc3
....aucacagccagcuuugaug..................	1	0	bc3
....aucacagccagcuuugaugug................	3	0	bc3
....aucacagccagcuuugaugugc...............	5	0	bc3
....ucacagccagcuuugaugugcA..............	1	1	bc3
....ucacagccagcuuugaugugcu..............	2	0	bc3
....cacagccagcuuugaugugc...............	1	0	bc3
....acagccagcuuugaugug................	2	0	bc3
....acagccagcuuugaugugc...............	24	0	bc3
....acagccagcuuugaugugcu..............	2	0	bc3
...................caucaaagcggugguuga...	2	0	bc6
...................caucaaagcggugguugau..	2	0	bc6
...................caucaaCgcggugguugau..	1	1	bc6
...................caucaaagcggugguugaug...	40	0	bc6
...................caucaaagcggAgguugaug...	1	1	bc6
...................caucaaagcggCgguugaug...	1	1	bc6
...................cCucaaagcggugguugaug...	1	1	bc6
...................caucaaagcggugguugaugu..	27	0	bc6
...................caucaaagcggGgguugaugu..	1	1	bc6
...................caucaaaCcggugguugaugu..	1	1	bc6
...................caucaaagcggugguugaugug...	15	0	bc6
...................caucaaagcggugguugauAug...	1	1	bc6
...................caucaaagcggugguugaugugu..	3	0	bc6
...................caucaaagcggugguugauguguugcaaauuaugacuuucauUu..................................	1	1	bc6
....uugcaaauuaugacuuuca......................................	4	0	bc6
....uugcaaauuaugacuuucUua....................................	1	1	bc6
....Aauaucacagccagcuuugaugugc...............	2	1	bc6
....uaucacagccagcuuuga....................	15	0	bc6
....uaucacagccagcuuugaug..................	20	0	bc6
....uaucacagccagAuuugaugu.................	1	1	bc6
....uaucacagccagcuuugaugu.................	19	0	bc6
....uaucacagccagcuuugauguC................	2	1	bc6
....uaucacagccaAcuuugaugug................	1	1	bc6
....uaucacagccagcuuugauUug................	1	1	bc6
....uaucacagccUgcuuugaugug................	1	1	bc6
....uaucacagccagcuuugauguA................	1	1	bc6
....uaucacagccagcuuugaugug................	328	0	bc6
....uaucacagccagcuuugaugGg................	1	1	bc6
....uaucacagccagcuuugauguU................	10	1	bc6
....uaucacagccagcuuugaugugc...............	5847	0	bc6
....uaucacaAccagcuuugaugugc...............	2	1	bc6
....uaucacagccagAuuugaugugc...............	2	1	bc6
....uaucacagccaAcuuugaugugc...............	1	1	bc6
....uaucaAagccagcuuugaugugc...............	1	1	bc6
....uaucacagccagcuuugaugugA...............	11	1	bc6
....uGucacagccagcuuugaugugc...............	1	1	bc6
....uaucacagccagcuuCgaugugc...............	2	1	bc6
....uaucCcagccagcuuugaugugc...............	1	1	bc6
....uaucacagccagcuuugaugugU...............	41	1	bc6
....uaucacagccagcuuugaugGgc...............	14	1	bc6
....uaucacagccGgcuuugaugugc...............	2	1	bc6
....uaucacagccagUuuugaugugc...............	1	1	bc6
....Aaucacagccagcuuugaugugc...............	1	1	bc6
....uauAacagccagcuuugaugugc...............	1	1	bc6
....uaucacagccagcuuugauguAc...............	2	1	bc6
....uaucacagccCgcuuugaugugc...............	4	1	bc6
....Caucacagccagcuuugaugugc...............	1	1	bc6
....uaucacagccagcuuugauCugc...............	3	1	bc6
....uaucacagccUgcuuugaugugc...............	2	1	bc6
....uaucGcagccagcuuugaugugc...............	1	1	bc6

```
                                                            cel-miR-2
                   cel-miR-2*
uaaacaguauacagaaagccaucaaagcggugguugauguguugcaaauuaugacuuucauaucacagccagcuuugaugugcugccuguugcacugu

............................................................uaucaUagccagcuuugaugugc...............    2     1     bc6
............................................................uaucacUgccagcuuugaugugc...............    1     1     bc6
............................................................uaucacagccagcuuuAaugugc...............    2     1     bc6
............................................................uauUacagccagcuuugaugugc...............    2     1     bc6
............................................................uaucacagccagcuuuUaugugc...............    3     1     bc6
............................................................uaucacagccagcuuugauguUc...............    1     1     bc6
............................................................uaCcacagccagcuuugaugugc...............    2     1     bc6
............................................................uaucacagcUagcuuugaugugc...............    4     1     bc6
............................................................uaucacagccagcuuugauUugc...............    3     1     bc6
............................................................uaucacagccagcCuugaugugc...............    2     1     bc6
............................................................uaucacagccagcuuugaugugG...............    44    1     bc6
............................................................uaucacagUcagcuuugaugugc...............    1     1     bc6
............................................................uaucacagccagcuuugaugCgc...............    5     1     bc6
............................................................uaucacagccagcuuugaugAgc...............    1     1     bc6
............................................................uaucacGgccagcuuugaugugc...............    3     1     bc6
............................................................uaGcacagccagcuuugaugugc...............    1     1     bc6
............................................................uaucacaCccagcuuugaugugc...............    2     1     bc6
............................................................uaucacagccagcuuugaCgugc...............    3     1     bc6
............................................................uaucacagccagcuuugaGgugc...............    2     1     bc6
............................................................uaucacagccagcuGugaugugc...............    1     1     bc6
............................................................uaucacagccagcuuugauAugc...............    7     1     bc6
............................................................uaucacCgccagcuuugaugugc...............    2     1     bc6
............................................................uaucacagccagcuuugUugugc...............    1     1     bc6
............................................................uaucacagccaAcuuugaugugcu..............    1     1     bc6
............................................................uaucacagccagcuuugaugugcG..............    1     1     bc6
............................................................uaucacagccagcuuugaugugcu..............    55    0     bc6
............................................................uaucacagccagcuuugaugugcA..............    8     1     bc6
............................................................uaucacagccagcuuugaugugcuU.............    1     1     bc6
.............................................................aucacagccagcuuugaugug................    1     0     bc6
.............................................................aucacagccagcuuugaugugA...............    1     1     bc6
.............................................................aucacagccagcuuugaugugc...............    11    0     bc6
.............................................................aucacagccagcuuugaugugcu..............    2     0     bc6
.............................................................aucacagccagcuuugaugugcA..............    3     1     bc6
..............................................................ucacagccagcuuugaugugc...............    2     0     bc6
..............................................................ucacagccagcuuugaugugcu..............    1     0     bc6
..............................................................ucacagccagcuuugaugugcA..............    1     1     bc6
................................................................acagccagcuuugaugug................    2     0     bc6
................................................................acagccagcuuugaugugc...............    34    0     bc6
.................................................................cagccagcuuugaugugc...............    9     0     bc6

...................caucaaagcggugguuga.............................................................    2     0     bc5
...................caucaaagcggGgguugaug...........................................................    1     1     bc5
...................caucaaagcggugguugaGg...........................................................    1     1     bc5
...................caucaaagcggugguugaug...........................................................    71    0     bc5
...................caucaaagcggugguugaugu..........................................................    42    0     bc5
...................caucaaagcgguggGugaugu..........................................................    1     1     bc5
...................caCcaaagcggugguugaugu..........................................................    1     1     bc5
...................caucaaagcggGgguugaugu..........................................................    1     1     bc5
...................caucaaagcggugguugaugug.........................................................    14    0     bc5
...................caucaaagcggugguugaugGg.........................................................    1     1     bc5
...................caucaaagcggugguugaAgug.........................................................    1     1     bc5
...................caucaaagcggugguugaugugu........................................................    2     0     bc5
...................caucaaagcggugguugauguguu.......................................................    1     0     bc5
....................................auguguugcaaauuaugacu..........................................    1     0     bc5
.........................................uugcaaauuaugacuuuca......................................    3     0     bc5
...........................................................Cuaucacagccagcuuugaugugc...............    2     1     bc5
............................................................uaucacagccagcuuuga....................    9     0     bc5
............................................................uaucacagccagcuuugaug..................    24    0     bc5
............................................................uaucacagccagcuuugaugu.................    16    0     bc5
............................................................uaucacagccagcuuugaugGg................    2     1     bc5
............................................................uaucacagccagcuuugauguU................    17    1     bc5
............................................................uaucacagccagcuuugCugug................    1     1     bc5
............................................................uaucacagccagcuuugauUug................    1     1     bc5
............................................................uaucacagccagcuuugUugug................    2     1     bc5
............................................................uaucacagccagcuuugaGgug................    1     1     bc5
............................................................uaucacCgccagcuuugaugug................    1     1     bc5
............................................................uaucacagccagcuuugauguC................    6     1     bc5
............................................................uaucacagccagcuuugaugug................    518   0     bc5
............................................................uaucacagccagcuuugauguA................    3     1     bc5
```

```
                                                            cel-miR-2
                   cel-miR-2*
uaaacaguauacagaaagccaucaaagcggugguugauguguugcaaauuaugacuuucauaucacagccagcuuugaugugcugccuguugcacugu

............................................................uaCcacagccagcuuugaugugc...............   2     1   bc5
............................................................uaucacagccagcuuugaugugG...............   34    1   bc5
............................................................uaucacagccagcuuugaugAgc...............   2     1   bc5
............................................................uaucacagAcagcuuugaugugc...............   1     1   bc5
............................................................uaucacUgccagcuuugaugugc...............   2     1   bc5
............................................................uaucacagccagcuuugaugGgc...............   14    1   bc5
............................................................uaucacagccagcCuugaugugc...............   5     1   bc5
............................................................uaucacagccagcuuugaCgugc...............   5     1   bc5
............................................................uaucacagccagcuuugaGgugc...............   3     1   bc5
............................................................Caucacagccagcuuugaugugc...............   1     1   bc5
............................................................uaucacagccagcuuugauAugc...............   9     1   bc5
............................................................uaucacagccCgcuuugaugugc...............   1     1   bc5
............................................................uaucacaCccagcuuugaugugc...............   2     1   bc5
............................................................uGucacagccagcuuugaugugc...............   2     1   bc5
............................................................uauGacagccagcuuugaugugc...............   3     1   bc5
............................................................uaucacagccagcuuugauUugc...............   6     1   bc5
............................................................uaucacagccagcuuugGugugc...............   4     1   bc5
............................................................uaucacagccaUcuuugaugugc...............   2     1   bc5
............................................................uaucacagGcagcuuugaugugc...............   1     1   bc5
............................................................uaucacagccagcuuugaugugc...............   8417  0   bc5
............................................................uaucacagccagcuuugCugugc...............   2     1   bc5
............................................................Aaucacagccagcuuugaugugc...............   1     1   bc5
............................................................uaucacagccGgcuuugaugugc...............   6     1   bc5
............................................................uauAacagccagcuuugaugugc...............   2     1   bc5
............................................................uCucacagccagcuuugaugugc...............   1     1   bc5
............................................................uaucacagccagcuuugUugugc...............   4     1   bc5
............................................................uaucacGgccagcuuugaugugc...............   2     1   bc5
............................................................uaucacagccagcuuugaugugA...............   16    1   bc5
............................................................uaucacagccagcuuuUaugugc...............   3     1   bc5
............................................................uaucaUagccagcuuugaugugc...............   4     1   bc5
............................................................uaGcacagccagcuuugaugugc...............   1     1   bc5
............................................................uaucacagccagcuuugauguAc...............   4     1   bc5
............................................................uaucacagccagcuCugaugugc...............   1     1   bc5
............................................................uaucacCgccagcuuugaugugc...............   1     1   bc5
............................................................uaucacagccagcuuuCaugugc...............   1     1   bc5
............................................................uaucacaUccagcuuugaugugc...............   2     1   bc5
............................................................uaucacagUcagcuuugaugugc...............   1     1   bc5
............................................................uaucacagccagGuuugaugugc...............   1     1   bc5
............................................................uaucacagccagcuuugauCugc...............   3     1   bc5
............................................................uaucacagccagcuuugaugCgc...............   3     1   bc5
............................................................uaucacagccagcuuugaugugU...............   48    1   bc5
............................................................uaucacagccagcuuugauguUc...............   2     1   bc5
............................................................uaucacagccagcuuuAaugugc...............   3     1   bc5
............................................................uaucacagccaAcuuugaugugc...............   1     1   bc5
............................................................uaucaAagccagcuuugaugugc...............   1     1   bc5
............................................................uaucacaAccagcuuugaugugc...............   1     1   bc5
............................................................uaucacagcUagcuuugaugugc...............   3     1   bc5
............................................................uaucacagccagcuuugauguCc...............   3     1   bc5
............................................................uaucacagcAagcuuugaugugc...............   2     1   bc5
............................................................uaucacagccagcuuugaugGgcu..............   1     1   bc5
............................................................uaucacagccagcuuugaugugcA..............   12    1   bc5
............................................................uaucacagccagcuuugaugugcC..............   1     1   bc5
............................................................uaucacagccagcuuugaugugcG..............   5     1   bc5
............................................................uaucacagccagcuuugaugugcu..............   79    0   bc5
.............................................................aucacagccagcuuugaugug................   1     0   bc5
.............................................................aucacagccagcuuugaugugc...............   14    0   bc5
.............................................................aucacagccagcuuugaugugA...............   1     1   bc5
.............................................................aucacagccagcuuugaugugcu..............   2     0   bc5
.............................................................aucacagccagcuuugaugugcC..............   1     1   bc5
..............................................................ucacagccagcuuugaugugc...............   2     0   bc5
..............................................................ucacagUcagcuuugaugugc...............   1     1   bc5
..............................................................ucacagccagcuuugaugugcu..............   4     0   bc5
..............................................................ucacagccagcuuugauguUu..............   1     1   bc5
..............................................................ucacagccagcuuugaugugcA..............   2     1   bc5
...............................................................cacagccagcuuugaugug................   2     0   bc5
................................................................acagccagcuuugaugug................   5     0   bc5
................................................................acagAcagcuuugaugugc...............   1     1   bc5
................................................................acagccagcuuugaugugc...............   41    0   bc5
.................................................................cagccagcuuugaugugc...............   9     0   bc5
```

cel-miR-2

cel-miR-2*

uaaacaguauacagaaagccaucaaagcggugguugauguguugcaaauuaugacuuucauaucacagccagcuuugaugugcugccuguugcacugu

...................caucaaagcggugguugau..	2	0	bc2
...................caucaaagcggugguugaug...	2	0	bc2
...................caucaaagcggugguugaugu..	3	0	bc2
...................caucaaaUcggugguugaugu..	1	1	bc2
...................caucaaagcggugguugaugug...	3	0	bc2
...................caucaaagcggugguugaugugu..	1	0	bc2
....................aucaaagcggugguugaugug...	2	0	bc2
....Cuaucacagccagcuuugaugugc...............	1	1	bc2
....uaucacagccagcuuuga....................	10	0	bc2
....uaucacagccagcuuugau...................	1	0	bc2
....uaucacagccagcuuugaug..................	11	0	bc2
....uaucacagccagcuuugaugu.................	7	0	bc2
....uaucacagccagcuuugauCu.................	1	1	bc2
....uaucacaCccagcuuugaugug................	1	1	bc2
....uaucacagccagcuuugauguA................	2	1	bc2
....uaucacagAcagcuuugaugug................	1	1	bc2
....uaucacagccagcuuugauguU................	11	1	bc2
....uaucacagccagcuuugaugCg................	1	1	bc2
....uaucacagccagcuuugaugug................	241	0	bc2
....uaucacagccagcuuugauguC................	3	1	bc2
....uaucacCgccagcuuugaugugc...............	1	1	bc2
....uaucacagccagcuuugaugGgc...............	11	1	bc2
....uaucacagccagcCuugaugugc...............	3	1	bc2
....uaucacagccagcuuugaGgugc...............	1	1	bc2
....uaucacagccagcuuuUaugugc...............	1	1	bc2
....uaucacagccagcuuugauguUc...............	1	1	bc2
....uaucacagccagcuuugaugugU...............	15	1	bc2
....uauGacagccagcuuugaugugc...............	1	1	bc2
....uaucacagccagGuuugaugugc...............	1	1	bc2
....uaucacagccagcuuugauguCc...............	2	1	bc2
....uaucaUagccagcuuugaugugc...............	1	1	bc2
....uaucacagccagcuuCgaugugc...............	2	1	bc2
....uaucacagccaCcuuugaugugc...............	1	1	bc2
....uaucacagccagcuuugaugugc...............	3361	0	bc2
....uaucacagcAagcuuugaugugc...............	3	1	bc2
....uaucacagccagcuuugaugugA...............	13	1	bc2
....uaucacagccagcuuugauCugc...............	4	1	bc2
....Caucacagccagcuuugaugugc...............	2	1	bc2
....uaucacagccCgcuuugaugugc...............	1	1	bc2
....uaucacagccagcuuugGugugc...............	1	1	bc2
....uaucacagcUagcuuugaugugc...............	1	1	bc2
....uaucacagccagcuuugCugugc...............	1	1	bc2
....uaucacaCccagcuuugaugugc...............	1	1	bc2
....uaucacagccUgcuuugaugugc...............	1	1	bc2
....uGucacagccagcuuugaugugc...............	1	1	bc2
....uaucacagccagcuuugauUugc...............	1	1	bc2
....uaucacagccagcuuugaugAgc...............	2	1	bc2
....uauAacagccagcuuugaugugc...............	1	1	bc2
....uaucaGagccagcuuugaugugc...............	1	1	bc2
....uaucaAagccagcuuugaugugc...............	1	1	bc2
....uaucacagccagcuuugaugCgc...............	1	1	bc2
....uaucacagccGgcuuugaugugc...............	2	1	bc2
....uaucacGgccagcuuugaugugc...............	3	1	bc2
....uaucacagccagcuuugaugugG...............	9	1	bc2
....uaucacagccagcuuugaugugcG..............	1	1	bc2
....uaucacagccagcuuugaugugcu..............	40	0	bc2
....uaucacagccagcuuugaugugUu..............	4	1	bc2
....uaucacagccagcuuugaugugcC..............	1	1	bc2
....uaucacagccagcuuugaugugcA..............	12	1	bc2
....aucacagccagcuuugaugugc...............	6	0	bc2
....aucacagccagcuuugaugugA...............	1	1	bc2
....aucacagccagcuuugaugugcu..............	2	0	bc2
....ucacagccagcuuugaugugc...............	1	0	bc2
....acagccagcuuugaugug................	1	0	bc2
....acagccagcuuugaugugc...............	21	0	bc2
....acagccagcuuuUaugugc...............	1	1	bc2

...................caucaaagcggugguugau..	2	0	bc7

cel-miR-2

cel-miR-2*

uaaacaguauacagaaagccaucaaagcggugguugauguguugcaaauuaugacuuucauaucacagccagcuuugaugugcugccuguugcacugu

...................caucaaagcggugguugaug...	26	0	bc7
...................caucaaagcggugguugaugu..	21	0	bc7
...................cUucaaagcggugguugaugu..	1	1	bc7
...................caucaaagcggugguugaugug...	6	0	bc7
....uugcaaauuaugacuuuca......................................	1	0	bc7
....Cuaucacagccagcuuugaugugc...............	1	1	bc7
....uaucacagccagcuuuga....................	8	0	bc7
....uaucacagccagcuuugau...................	1	0	bc7
....uaucaUagccagcuuugaug..................	1	1	bc7
....uaucacagccagcuuugaug..................	44	0	bc7
....uaucacagccagcuuugaugu.................	16	0	bc7
....uaucacagccagcuuugaugug................	524	0	bc7
....uaucacagccagcuuugauguC................	2	1	bc7
....uaucacagccagcuuugauguA................	2	1	bc7
....uaucacagccagcuuugaugGg................	4	1	bc7
....uaucacagccagcuuuUaugug................	1	1	bc7
....uaucacagccagcuuugauguU................	12	1	bc7
....uaucacGgccagcuuugaugug................	1	1	bc7
....uaucaUagccagcuuugaugug................	1	1	bc7
....uaucacaCccagcuuugaugugc...............	1	1	bc7
....uaucacagccaUcuuugaugugc...............	1	1	bc7
....uaucacagcUagcuuugaugugc...............	4	1	bc7
....uaucacagccagcuuugaugCgc...............	3	1	bc7
....uaucaUagccagcuuugaugugc...............	3	1	bc7
....uaucacGgccagcuuugaugugc...............	2	1	bc7
....uaucacagccagUuuugaugugc...............	2	1	bc7
....uaucacagccagcuGugaugugc...............	1	1	bc7
....uaucacagcAagcuuugaugugc...............	2	1	bc7
....uaucacagccGgcuuugaugugc...............	6	1	bc7
....uauAacagccagcuuugaugugc...............	1	1	bc7
....uaucacagccagcuuuAaugugc...............	4	1	bc7
....uaucacCgccagcuuugaugugc...............	1	1	bc7
....uaucacagccagcuuugaAgugc...............	2	1	bc7
....uaucacagccagcuuugUugugc...............	3	1	bc7
....uaucUcagccagcuuugaugugc...............	1	1	bc7
....uaucacagccagcuuuCaugugc...............	2	1	bc7
....uaucacagccagcuuugaugugc...............	8093	0	bc7
....uaucacaAccagcuuugaugugc...............	2	1	bc7
....uaucacagccagcuuugGugugc...............	1	1	bc7
....Caucacagccagcuuugaugugc...............	2	1	bc7
....uaucacagccagcuuugCugugc...............	1	1	bc7
....uaucacagccCgcuuugaugugc...............	2	1	bc7
....uaucacagccagcuuugaugugU...............	40	1	bc7
....uaAcacagccagcuuugaugugc...............	2	1	bc7
....uaucacagccagcuuugaCgugc...............	1	1	bc7
....uaucacagccagAuuugaugugc...............	2	1	bc7
....uaucacagccagcuuugaugugA...............	17	1	bc7
....uaucacagccagcuuugauAugc...............	7	1	bc7
....uaucacagccagcCuugaugugc...............	4	1	bc7
....uauUacagccagcuuugaugugc...............	2	1	bc7
....uaucacagccagcuuugauguUc...............	3	1	bc7
....uaucacagccagcuuugaugAgc...............	2	1	bc7
....uaucacagccagcuuuUaugugc...............	6	1	bc7
....uaucacaUccagcuuugaugugc...............	1	1	bc7
....uaucacagccagcAuugaugugc...............	1	1	bc7
....uaucacagccagcuuugauUugc...............	6	1	bc7
....uaucacagccagcuuugauguCc...............	3	1	bc7
....uaucaAagccagcuuugaugugc...............	2	1	bc7
....uaucacagccagcuuugaGgugc...............	3	1	bc7
....uaucacagccagcuuugaugGgc...............	22	1	bc7
....uaucacagccagcuuugauguAc...............	2	1	bc7
....uaucCcagccagcuuugaugugc...............	1	1	bc7
....uaucacagccagcuuugaugugG...............	63	1	bc7
....uaucacagccagcuCugaugugc...............	1	1	bc7
....uaucacagccagcuuugauCugc...............	3	1	bc7
....uaucacagAcagcuuugaugugc...............	2	1	bc7
....uaucacagccagcuAugaugugc...............	2	1	bc7
....uGucacagccagcuuugaugugc...............	2	1	bc7
....uaucacagccagcuuCgaugugc...............	3	1	bc7

```
                                                            cel-miR-2
                   cel-miR-2*
uaaacaguauacagaaagccaucaaagcggugguugauguguugcaaauuaugacuuucauaucacagccagcuuugaugugcugccuguugcacugu

............................................................uaucGcagccagcuuugaugugc...............    6    1    bc7
............................................................uaucacagccagcuuugaugugcA..............    7    1    bc7
............................................................uaucacagccagcuuugaugugcC..............    1    1    bc7
............................................................uaucacagccagcuuugaugugcG..............    4    1    bc7
............................................................uaucacagccagcuuugaugugUu..............    3    1    bc7
............................................................uaucacagccagcuuugaugugcu..............    74   0    bc7
.............................................................aucacagccagcuuugaugug................    1    0    bc7
.............................................................aucacagccagcuuugaugugc...............    14   0    bc7
.............................................................aucacagccagcuuugaugugcu..............    4    0    bc7
..............................................................ucacagccagcuuugaugugc...............    3    0    bc7
..............................................................ucacagccagcuuugaugugcu..............    1    0    bc7
..............................................................ucacagccagcuuugaugugcC..............    1    1    bc7
................................................................acagccagcuuugaugCg................    1    1    bc7
................................................................acagccagcuuugaugug................    1    0    bc7
................................................................acagccagcuuugaugugG...............    1    1    bc7
................................................................acagccagcuuugaugugc...............    41   0    bc7
.................................................................cagccagcuuugaugugc...............    8    0    bc7
```
