# Supplementary material for: Reliable reference miRNAs for quantitative gene expression analysis of stress responses in Caenorhabditis elegans
Source: BMC Genomics. 2014 Mar 21;15:222. doi: 10.1186/1471-2164-15-222 (PMC3997968; doi:10.1186/1471-2164-15-222)
Supplement: Additional file 5: Figure S2 — Graphical output files from mirDeep2 showing the reads, counts per read and mapping on the hairpin for mir-2, mir-46 and mir-47. [file 1471-2164-15-222-S5.pdf]

miRBase precursor : cel-mir-46
Total read count : 175984
cel-miR-46 read count : 146816
cel-miR-46* read count : 29080
remaining reads : 88

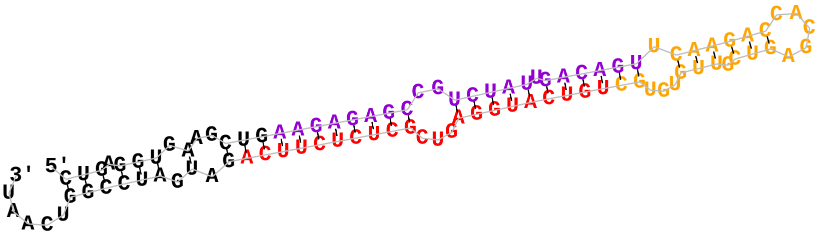

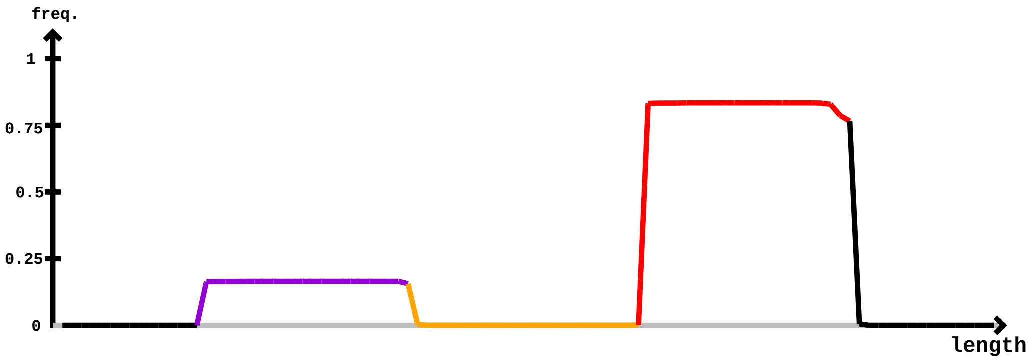

cel-miR-46*

cel-miR-46

```
5'- cugaggugaagcugaagagagccgucuauugacaguucaagaccacgagucguugugugcugucauggagucgcucucuucagaugauccggucaau   -3'    exp
    ....((.....((((((((((..(((((.((((((.((((((.....))).))).)))...))))))))))...)))))))))).....)).......    reads      mm       sample
    .............Caagagagccgucuauugacagu.............................................................    3          1        bc4
    ..............aagagagccgucuauugacag..............................................................    55         0        bc4
    ..............aagagagcAgucuauugacag..............................................................    29         1        bc4
    ..............aagagagcUgucuauugacag..............................................................    1          1        bc4
    ..............aagagagcAgucuauugacagu.............................................................    542        1        bc4
    ..............aagagagccgucuUuugacagu.............................................................    1          1        bc4
    ..............aagagagccgucuauugacaAu.............................................................    1          1        bc4
    ..............aagagaCccgucuauugacagu.............................................................    1          1        bc4
    ..............Uagagagccgucuauugacagu.............................................................    1          1        bc4
    ..............aagagagUcgucuauugacagu.............................................................    1          1        bc4
    ..............aagagagccgucuauugCcagu.............................................................    2          1        bc4
    ..............aagaUagccgucuauugacagu.............................................................    1          1        bc4
    ..............aGgagagccgucuauugacagu.............................................................    2          1        bc4
    ..............aagagagccgucuauugacagu.............................................................    941        0        bc4
    ..............aagagagccgucuauugacCgu.............................................................    1          1        bc4
    ..............aagagagccgucuauugacagA.............................................................    1          1        bc4
    ..............aagagagccguGuauugacagu.............................................................    1          1        bc4
    ..............aagagagccgucuGuugacagu.............................................................    2          1        bc4
    ..............aagagagccgucuauugacagG.............................................................    3          1        bc4
    ..............aagagaAccgucuauugacagu.............................................................    2          1        bc4
    ..............aagagagccgucuauAgacagu.............................................................    1          1        bc4
    ..............aagGgagccgucuauugacagu.............................................................    1          1        bc4
    ..............Gagagagccgucuauugacagu.............................................................    1          1        bc4
    ..............aagagagAcgucuauugacagu.............................................................    1          1        bc4
    ..............aagagagccgucuauugacagC.............................................................    1          1        bc4
    ..............aagagagccgucuauugacaguG............................................................    1          1        bc4
    ..............aagagagcAgucuauugacaguu............................................................    9          1        bc4
    ..............aagagagccgucuauugacaguu............................................................    12         0        bc4
    ..............aagagagccgucuauugacaguuU...........................................................    3          1        bc4
    ...............agagagccgucuauugacagu.............................................................    11         0        bc4
    ...............agagagccgucuauugacaguu............................................................    1          0        bc4
    ..................gagccgucuauugacagu.............................................................    1          0        bc4
    ..................gagcAgucuauugacagu.............................................................    1          1        bc4
    ....................................ucaagaccacgagucguugugugcu....................................    1          0        bc4
```

```
              cel-miR-46*
                                                cel-miR-46
cugaggugaagcugaagagagccgucuauugacaguucaagaccacgagucguugugugcugucauggagucgcucucuucagaugauccggucaau

....................................ucaagaccacgagucguugugugcuCu..................................    1     1     bc4
....................................ucaagaccacgagucguugugugcugucaugga............................    1     0     bc4
....................................ucaagaccacgagucguugugugcugucauggagucgcucucuuca...............    3     0     bc4
....................................ucaagaccacUagucguugugugcugucauggagucgcucucuuca...............    1     1     bc4
......................................aagaccacgagucguugugugcugucauggagucgcucucuuca...............    1     0     bc4
...........................................................cugucauggagucgcucucuu.................    1     0     bc4
...........................................................cugucauggagucgcucucuucU...............    1     1     bc4
...........................................................cugucauggagucgcucucuuca...............    10    0     bc4
...........................................................cugucauggagGcgcucucuuca...............    3     1     bc4
............................................................ugucauggagGcgcucuc...................    3     1     bc4
............................................................ugucauggagucgcucuc...................    6     0     bc4
............................................................ugucauggagGcgcucucu..................    26    1     bc4
............................................................ugucauggagucgcucucu..................    7     0     bc4
............................................................ugucauggagAcgcucucuu.................    1     1     bc4
............................................................ugucauggagucgcucucuG.................    1     1     bc4
............................................................ugucauggaguAgcucucuu.................    2     1     bc4
............................................................ugucauggagucgcucucuu.................    130   0     bc4
............................................................ugucauggagGcgcucucuu.................    234   1     bc4
............................................................ugucauggagCcgcucucuu.................    1     1     bc4
............................................................ugucauggagucgcucucuuc................    78    0     bc4
............................................................ugucauggagucgcucucuuU................    1     1     bc4
............................................................ugucauggagGcgcucucuuc................    93    1     bc4
............................................................ugucauggagCcgcucucuuc................    1     1     bc4
............................................................Cgucauggagucgcucucuuca...............    2     1     bc4
............................................................ugucauggaguGgcucucuuca...............    1     1     bc4
............................................................ugucauggagucgcucucuuca...............    5628  0     bc4
............................................................ugucauggagucgcucuAuuca...............    5     1     bc4
............................................................ugucauggaUucgcucucuuca...............    1     1     bc4
............................................................ugucauggagucgcucucuGca...............    8     1     bc4
............................................................ugucauggagucgGucucuuca...............    1     1     bc4
............................................................ugucauggagucUcucucuuca...............    5     1     bc4
............................................................ugucauggagucgcucucuucC...............    10    1     bc4
............................................................ugucauUgagucgcucucuuca...............    4     1     bc4
............................................................ugucauggagucgcCcucuuca...............    1     1     bc4
............................................................ugucauggagucgUucucuuca...............    1     1     bc4
............................................................ugucaugAagucgcucucuuca...............    1     1     bc4
............................................................ugucauggagucgcucucuAca...............    7     1     bc4
............................................................ugucaugUagucgcucucuuca...............    1     1     bc4
............................................................ugucGuggagucgcucucuuca...............    2     1     bc4
............................................................ugucauggagAcgcucucuuca...............    4     1     bc4
............................................................ugucauggGgucgcucucuuca...............    5     1     bc4
............................................................ugucauggagCcgcucucuuca...............    8     1     bc4
............................................................ugucauggagucgcuGucuuca...............    1     1     bc4
............................................................uguGauggagucgcucucuuca...............    2     1     bc4
............................................................ugucaCggagucgcucucuuca...............    2     1     bc4
............................................................uguUauggagucgcucucuuca...............    1     1     bc4
............................................................ugucauAgagucgcucucuuca...............    1     1     bc4
............................................................ugucauggagGcgcucucuuca...............    1930  1     bc4
............................................................ugucauggaAucgcucucuuca...............    1     1     bc4
............................................................ugucauggCgucgcucucuuca...............    2     1     bc4
............................................................Ggucauggagucgcucucuuca...............    2     1     bc4
............................................................ugucauggagucgcucucuucG...............    10    1     bc4
............................................................ugucauggagucCcucucuuca...............    3     1     bc4
............................................................ugucauggagucgcucCcuuca...............    2     1     bc4
............................................................ugucaugCagucgcucucuuca...............    1     1     bc4
............................................................ugucauggagucgcucucuCca...............    5     1     bc4
............................................................ugucauggagucgcucucCuca...............    2     1     bc4
............................................................ugucauggagucgcucuUuuca...............    2     1     bc4
............................................................ugAcauggagucgcucucuuca...............    1     1     bc4
............................................................ugucUuggagucgcucucuuca...............    1     1     bc4
............................................................ugucauggagucgcucucuuGa...............    1     1     bc4
............................................................ugGcauggagucgcucucuuca...............    1     1     bc4
............................................................ugucauggagucgcuAucuuca...............    1     1     bc4
............................................................ugucauggaguAgcucucuuca...............    4     1     bc4
............................................................ugucauggagucgcuUucuuca...............    5     1     bc4
............................................................ugucauggagucAcucucuuca...............    1     1     bc4
............................................................ugucauggUgucgcucucuuca...............    1     1     bc4
............................................................uUucauggagucgcucucuuca...............    1     1     bc4
............................................................ugucauggaguUgcucucuuca...............    4     1     bc4
```

```
              cel-miR-46*
                                                            cel-miR-46
cugaggugaagcugaagagagccgucuauugacaguucaagaccacgagucguugugugcugucauggagucgcucucuucagaugauccggucaau

............................................................ugucauggagucgAucucuuca...............   1      1   bc4
............................................................ugucauggagucgcucucuucU...............   394    1   bc4
............................................................ugucauggagucgcucucuucaC..............   1      1   bc4
............................................................ugucauggagucgcucucuucaU..............   46     1   bc4
............................................................ugucauggagucgcucucuucag..............   3      0   bc4
............................................................ugucauggagucgcucucuucaA..............   3      1   bc4
............................................................ugucauggagucgcucucuucaAa.............   1      1   bc4
.............................................................gucauggagucgcucucuuca...............   8      0   bc4
.............................................................gucauggagGcgcucucuuca...............   3      1   bc4
.............................................................gucauggagucgcucucuucU...............   1      1   bc4
..............................................................ucauggagucgcucucuuca...............   1      0   bc4
................................................................auggagucgcucucuuca...............   3      0   bc4
................................................................auggagGcgcucucuuca...............   1      1   bc4

..........gcugaagagagccgucuauugacagu.............................................................   1      0   bc1
..............aagagagccgucuauugac................................................................   1      0   bc1
..............aagagagcAgucuauugaca...............................................................   1      1   bc1
..............aagagagccgucuauugacag..............................................................   73     0   bc1
..............aagagagcAgucuauugacag..............................................................   42     1   bc1
..............aagagagccgucuauugacCg..............................................................   1      1   bc1
..............aagagagccgucuauugacGgu.............................................................   1      1   bc1
..............aaUagagccgucuauugacagu.............................................................   1      1   bc1
..............aagagagccgucuauugacaCu.............................................................   1      1   bc1
..............aagagUgccgucuauugacagu.............................................................   1      1   bc1
..............aagagagUcgucuauugacagu.............................................................   2      1   bc1
..............aagagagccgucuauugacaUu.............................................................   1      1   bc1
..............aagagagccgucuauCgacagu.............................................................   1      1   bc1
..............aagagagccgucuauugacagu.............................................................   1238   0   bc1
..............aagagaCccgucuauugacagu.............................................................   1      1   bc1
..............aagagaUccgucuauugacagu.............................................................   1      1   bc1
..............aagagGgccgucuauugacagu.............................................................   1      1   bc1
..............aagagagccgucuaCugacagu.............................................................   2      1   bc1
..............Uagagagccgucuauugacagu.............................................................   1      1   bc1
..............aagagagcAgucuauugacagu.............................................................   786    1   bc1
..............aagagagccgucuauugacagC.............................................................   1      1   bc1
..............aagagagccgucuauuAacagu.............................................................   1      1   bc1
..............aagagagccgucuauugacagG.............................................................   4      1   bc1
..............aagagagccAucuauugacagu.............................................................   2      1   bc1
..............aagagagcAgucuauugacaguu............................................................   16     1   bc1
..............aagagagccgucuauugacaguu............................................................   16     0   bc1
...............agagagccgucuauugacagu.............................................................   15     0   bc1
...............agagagcAgucuauugacagu.............................................................   1      1   bc1
...............agagagccgucuauugacaguu............................................................   1      0   bc1
................gagagccgucuauugacagu.............................................................   1      0   bc1
..................gagcAgucuauugacagu.............................................................   3      1   bc1
..................gagccgucuauugacagu.............................................................   1      0   bc1
..................gaCccgucuauugacagu.............................................................   1      1   bc1
....................................ucaagaccacgagucguugugugc.....................................   1      0   bc1
....................................ucaaUaccacgagucguugugugcu....................................   1      1   bc1
....................................ucaagaccacgagucguugugugcu....................................   3      0   bc1
....................................ucaagaccacgagucguugugugcugucauggagucgcucucuuca...............   1      0   bc1
...........................................................cugucauggagGcgcucucuuca...............   2      1   bc1
...........................................................cugucauggagucgcucucuuca...............   5      0   bc1
...........................................................cugucauggagucgcucucuucU...............   1      1   bc1
............................................................ugucauggagGcgcucuc...................   4      1   bc1
............................................................ugucauggagucgcucuc...................   5      0   bc1
............................................................ugucauggaguGgcucuc...................   1      1   bc1
............................................................ugucauggagucgcucucu..................   6      0   bc1
............................................................ugucauggagGcgcucucu..................   24     1   bc1
............................................................ugucaGggagucgcucucuu.................   1      1   bc1
............................................................ugucauggagucgcucucuG.................   1      1   bc1
............................................................uAucauggagucgcucucuu.................   1      1   bc1
............................................................ugucauggGgucgcucucuu.................   1      1   bc1
............................................................ugucauggagGcgcucucuu.................   238    1   bc1
............................................................ugucauggagucgcucucuu.................   143    0   bc1
............................................................Cgucauggagucgcucucuuc................   1      1   bc1
............................................................ugucauggagucgcucucuuc................   67     0   bc1
............................................................ugucauggagucgcucCcuuc................   1      1   bc1
............................................................ugucauggaUucgcucucuuc................   1      1   bc1
```

```
              cel-miR-46*
                                                            cel-miR-46
cugaggugaagcugaagagagccgucuauugacaguucaagaccacgagucguugugugcugucauggagucgcucucuucagaugauccggucaau

............................................................ugucauggagGcgcucucuuc................    155     1     bc1
............................................................ugucauggagucgcucucuuU................    1       1     bc1
............................................................ugucauggagucgcucucCuca...............    1       1     bc1
............................................................ugucauggagucgAucucuuca...............    3       1     bc1
............................................................ugucauggaCucgcucucuuca...............    1       1     bc1
............................................................ugucauggUgucgcucucuuca...............    2       1     bc1
............................................................ugucauggagucgcucucuAca...............    4       1     bc1
............................................................ugGcauggagucgcucucuuca...............    1       1     bc1
............................................................ugucauggagucgcucucuucC...............    12      1     bc1
............................................................uCucauggagucgcucucuuca...............    1       1     bc1
............................................................ugucauggagucgcucucuGca...............    3       1     bc1
............................................................ugucauggagucgcCcucuuca...............    2       1     bc1
............................................................uUucauggagucgcucucuuca...............    1       1     bc1
............................................................ugucauggagucgcGcucuuca...............    1       1     bc1
............................................................ugucauggagucgcucucuucG...............    7       1     bc1
............................................................uguAauggagucgcucucuuca...............    2       1     bc1
............................................................ugucauggagucgcucucuuca...............    7108    0     bc1
............................................................ugucauggagucCcucucuuca...............    2       1     bc1
............................................................ugucauggagGcgcucucuuca...............    2911    1     bc1
............................................................ugucauggagAcgcucucuuca...............    4       1     bc1
............................................................ugucauggagucgcucucuuUa...............    5       1     bc1
............................................................ugucaCggagucgcucucuuca...............    1       1     bc1
............................................................ugucauggaUucgcucucuuca...............    2       1     bc1
............................................................Agucauggagucgcucucuuca...............    3       1     bc1
............................................................ugucauggagucgUucucuuca...............    5       1     bc1
............................................................ugucauggagucgcucucuCca...............    6       1     bc1
............................................................ugucauggaAucgcucucuuca...............    2       1     bc1
............................................................uguUauggagucgcucucuuca...............    4       1     bc1
............................................................ugucauggagucgcuUucuuca...............    1       1     bc1
............................................................ugucauggagucUcucucuuca...............    3       1     bc1
............................................................ugucauggagucgcuAucuuca...............    2       1     bc1
............................................................ugucauAgagucgcucucuuca...............    2       1     bc1
............................................................ugucauggGgucgcucucuuca...............    6       1     bc1
............................................................ugucauggaguAgcucucuuca...............    6       1     bc1
............................................................ugucauggaguGgcucucuuca...............    1       1     bc1
............................................................ugucauggagCcgcucucuuca...............    13      1     bc1
............................................................ugucauggagucgcucuAuuca...............    2       1     bc1
............................................................ugucauggagucgcucucuucU...............    480     1     bc1
............................................................ugucauggCgucgcucucuuca...............    3       1     bc1
............................................................ugucauggagucgcucAcuuca...............    1       1     bc1
............................................................ugucaugCagucgcucucuuca...............    1       1     bc1
............................................................Cgucauggagucgcucucuuca...............    1       1     bc1
............................................................ugucCuggagucgcucucuuca...............    1       1     bc1
............................................................ugucauggagucgcucuUuuca...............    2       1     bc1
............................................................ugucGuggagucgcucucuuca...............    3       1     bc1
............................................................ugucauggagucgcucCcuuca...............    2       1     bc1
............................................................uAucauggagucgcucucuuca...............    1       1     bc1
............................................................ugucauggaguUgcucucuuca...............    3       1     bc1
............................................................ugucUuggagucgcucucuuca...............    1       1     bc1
............................................................ugucauggagucgcuGucuuca...............    1       1     bc1
............................................................ugucaugAagucgcucucuuca...............    2       1     bc1
............................................................ugucauggagucAcucucuuca...............    5       1     bc1
............................................................ugucaugUagucgcucucuuca...............    3       1     bc1
............................................................ugAcauggagucgcucucuuca...............    1       1     bc1
............................................................ugucauggagucgcucucuucaA..............    6       1     bc1
............................................................ugucauggagucgcucucuucaU..............    36      1     bc1
............................................................ugucauggagucgcucucuucaC..............    2       1     bc1
.............................................................gucauggagucgcucucuuc................    1       0     bc1
.............................................................gucauggagGcgcucucuuca...............    3       1     bc1
.............................................................gucauggagucgcucucuuca...............    15      0     bc1
.............................................................gucauggagucgcucucuucU...............    1       1     bc1
..............................................................ucauggagucgcucucuuca...............    1       0     bc1
..............................................................ucauggagGcgcucucuucaga.............    1       1     bc1
................................................................auggagGcgcucucuuca...............    3       1     bc1
................................................................auggagucgcucucuuca...............    2       0     bc1

.............Caagagagccgucuauugacagu.............................................................    4       1     bc3
..............aagagagccgucuauugac................................................................    2       0     bc3
..............aagagagccgucuauugaca...............................................................    1       0     bc3
```

```
              cel-miR-46*
                                                            cel-miR-46
cugaggugaagcugaagagagccgucuauugacaguucaagaccacgagucguugugugcugucauggagucgcucucuucagaugauccggucaau

..............aagagagcAgucuauugaca...............................................................    1      1      bc3
..............aagagagccgucuauugCcag..............................................................    1      1      bc3
..............aagagagcAgucuauugacag..............................................................    67     1      bc3
..............aagagagccgucuUuugacag..............................................................    1      1      bc3
..............aagagagccgucuauugacGg..............................................................    1      1      bc3
..............aagagagccgucuauugacag..............................................................    203    0      bc3
..............aagagagcGgucuauugacag..............................................................    1      1      bc3
..............aagagagcAgucuauugacagu.............................................................    1096   1      bc3
..............aagagagccgucuauugacagu.............................................................    2684   0      bc3
..............aagagagccgucuauugaUagu.............................................................    1      1      bc3
..............aagagagccgucuauGgacagu.............................................................    1      1      bc3
..............aagagagccgucuauugaAagu.............................................................    2      1      bc3
..............aaUagagccgucuauugacagu.............................................................    2      1      bc3
..............aagagagccgucuauugacCgu.............................................................    1      1      bc3
..............aagagagccgucuauugacaAu.............................................................    2      1      bc3
..............Uagagagccgucuauugacagu.............................................................    2      1      bc3
..............aagagaAccgucuauugacagu.............................................................    1      1      bc3
..............aagagaUccgucuauugacagu.............................................................    2      1      bc3
..............aagagagccgucuauugacagG.............................................................    6      1      bc3
..............aagagUgccgucuauugacagu.............................................................    1      1      bc3
..............Cagagagccgucuauugacagu.............................................................    1      1      bc3
..............aagagagccgGcuauugacagu.............................................................    2      1      bc3
..............aagagagccgucuaCugacagu.............................................................    1      1      bc3
..............aagagagccgucuauuAacagu.............................................................    2      1      bc3
..............aaCagagccgucuauugacagu.............................................................    1      1      bc3
..............aagagagccgCcuauugacagu.............................................................    4      1      bc3
..............aagagagccgucuauugGcagu.............................................................    1      1      bc3
..............aagagagccgucuauugacagC.............................................................    1      1      bc3
..............aagagagccguAuauugacagu.............................................................    5      1      bc3
..............aGgagagccgucuauugacagu.............................................................    1      1      bc3
..............aagagagccgucuauuUacagu.............................................................    1      1      bc3
..............aagaUagccgucuauugacagu.............................................................    1      1      bc3
..............aagagagccgucuauugacaCu.............................................................    1      1      bc3
..............aagagagccgucuauugacGgu.............................................................    6      1      bc3
..............aagagagccgucuauugacUgu.............................................................    1      1      bc3
..............aagagagcUgucuauugacagu.............................................................    1      1      bc3
..............aagaAagccgucuauugacagu.............................................................    1      1      bc3
..............aagagagcAgucuauugacaguu............................................................    17     1      bc3
..............aagagagccgucuauugacaguu............................................................    32     0      bc3
..............aagagagccgucuCuugacaguu............................................................    1      1      bc3
..............aagagagccgucuauugacaguuU...........................................................    4      1      bc3
...............agagagccgucuauugacag..............................................................    3      0      bc3
...............agagagccgucuauugacagu.............................................................    22     0      bc3
...............agagagcAgucuauugacagu.............................................................    1      1      bc3
...............agagagccgucuauugacaguu............................................................    1      0      bc3
................Aagagccgucuauugacagu.............................................................    1      1      bc3
................gagagccgucuauugacagu.............................................................    1      0      bc3
.................agagcAgucuauugacagu.............................................................    1      1      bc3
..................gagccgucuauugacagu.............................................................    4      0      bc3
..................gagcAgucuauugacagu.............................................................    2      1      bc3
....................................ucaagaccacgagucguugugugc.....................................    5      0      bc3
....................................ucaagaccacgagucguugugugcu....................................    4      0      bc3
....................................ucaagaccacgagucguugugugcuU...................................    2      1      bc3
....................................ucaagaccacgagucguugugugcugu..................................    1      0      bc3
....................................ucaagaccacgagucguugugugcuUu..................................    1      1      bc3
....................................ucaagaccacgagucguugugugcugucauggagucgcucucuuca...............    2      0      bc3
...........................................................cugucauggagucgcucucuuc................    1      0      bc3
...........................................................cugucauggagGcgcucucuuc................    1      1      bc3
...........................................................cugucauggagGcgcucucuuca...............    2      1      bc3
...........................................................cugucauggagucgcucucAuca...............    1      1      bc3
...........................................................cugucauggagucgcucucuuca...............    10     0      bc3
...........................................................cugucauggagucgcucucuucU...............    3      1      bc3
............................................................ugucauggagucgcucuc...................    15     0      bc3
............................................................ugucauggagGcgcucuc...................    10     1      bc3
............................................................ugucauggagucgcucuA...................    1      1      bc3
............................................................ugucauggagucgcucucu..................    27     0      bc3
............................................................ugucauggagGcgcucucu..................    74     1      bc3
............................................................ugucauggagucgcucuAu..................    1      1      bc3
............................................................ugucauggagCcgcucucuu.................    3      1      bc3
```

cel-miR-46*

cel-miR-46

cugaggugaagcugaagagagccgucuauugacaguucaagaccacgagucguugugugcugucauggagucgcucucuucagaugauccggucaau

....ugucauggagAcgcucucuu.................	1	1	bc3
....ugucauggaguAgcucucuu.................	1	1	bc3
....ugucauggagucgcucucuG.................	1	1	bc3
....ugucauggagucgcucuAuu.................	1	1	bc3
....ugucauggagGcgcucucuu.................	569	1	bc3
....ugucauggagucgcucucuu.................	385	0	bc3
....ugucUuggagucgcucucuuc................	1	1	bc3
....ugucauggagucgcucucuuU................	1	1	bc3
....ugucauggagucgcucucuuc................	172	0	bc3
....ugucauggagucgcucucuGc................	1	1	bc3
....ugucauAgagucgcucucuuc................	1	1	bc3
....ugucauggagGcgcucucuuc................	303	1	bc3
....ugucauggagucgUucucuuca...............	2	1	bc3
....ugucauggaUucgcucucuuca...............	4	1	bc3
....ugucauggagucgcucucuuAa...............	8	1	bc3
....ugucauggagucgAucucuuca...............	5	1	bc3
....uguGauggagucgcucucuuca...............	2	1	bc3
....ugucauggagucgGucucuuca...............	1	1	bc3
....ugucauggGgucgcucucuuca...............	8	1	bc3
....ugucauUgagucgcucucuuca...............	6	1	bc3
....ugucauAgagucgcucucuuca...............	3	1	bc3
....ugucGuggagucgcucucuuca...............	4	1	bc3
....ugucauggagucgcucucuuGa...............	3	1	bc3
....ugucauggagucgcucucuCca...............	11	1	bc3
....ugucCuggagucgcucucuuca...............	1	1	bc3
....uUucauggagucgcucucuuca...............	5	1	bc3
....ugucauggagucgcucucuAca...............	11	1	bc3
....ugucauggagucgcCcucuuca...............	2	1	bc3
....ugucauggagucgcucCcuuca...............	4	1	bc3
....ugucauggagucgcucucuucC...............	36	1	bc3
....ugucauggagucgcucucuuUa...............	9	1	bc3
....ugucauggagucgcuGucuuca...............	1	1	bc3
....ugucaugUagucgcucucuuca...............	4	1	bc3
....ugucauggaguGgcucucuuca...............	1	1	bc3
....ugucauggagucgcGcucuuca...............	1	1	bc3
....ugucauggagucgcucucuGca...............	5	1	bc3
....ugucauggaCucgcucucuuca...............	2	1	bc3
....ugucauggagucgcucuAuuca...............	7	1	bc3
....ugucauggagAcgcucucuuca...............	10	1	bc3
....ugAcauggagucgcucucuuca...............	7	1	bc3
....ugucauggagucgcucGcuuca...............	1	1	bc3
....ugucauggagGcgcucucuuca...............	4629	1	bc3
....uguUauggagucgcucucuuca...............	3	1	bc3
....Cgucauggagucgcucucuuca...............	3	1	bc3
....ugucauggagucgcucucuucG...............	18	1	bc3
....ugucauggCgucgcucucuuca...............	1	1	bc3
....uAucauggagucgcucucuuca...............	4	1	bc3
....ugucauggagucgcucuUuuca...............	1	1	bc3
....ugGcauggagucgcucucuuca...............	4	1	bc3
....ugucUuggagucgcucucuuca...............	1	1	bc3
....ugucauggaAucgcucucuuca...............	2	1	bc3
....ugucauggaguAgcucucuuca...............	2	1	bc3
....ugucaugAagucgcucucuuca...............	1	1	bc3
....ugucauggagucgcucucCuca...............	1	1	bc3
....ugucauggagucgcuAucuuca...............	15	1	bc3
....ugucauggUgucgcucucuuca...............	8	1	bc3
....ugucauggagucUcucucuuca...............	8	1	bc3
....ugucauggagCcgcucucuuca...............	30	1	bc3
....ugucauggagucgcucucuuca...............	14521	0	bc3
....ugucauggagucAcucucuuca...............	6	1	bc3
....ugucauggagucgcuUucuuca...............	6	1	bc3
....uguAauggagucgcucucuuca...............	2	1	bc3
....ugucauggagucgcucucuucU...............	1000	1	bc3
....ugCcauggagucgcucucuuca...............	3	1	bc3
....ugucauggaguUgcucucuuca...............	9	1	bc3
....ugucauggagucCcucucuuca...............	1	1	bc3
....ugucauCgagucgcucucuuca...............	1	1	bc3
....ugucaCggagucgcucucuuca...............	3	1	bc3
....Agucauggagucgcucucuuca...............	4	1	bc3

cel-miR-46*

cel-miR-46

```
cugaggugaagcugaagagagccgucuauugacaguucaagaccacgagucguugugugcugucauggagucgcucucuucagaugauccggucaau

............................................................ugucaugCagucgcucucuuca...............   2     1   bc3
............................................................ugucauggagucgcucucuucag..............   4     0   bc3
............................................................ugucauggagucgcucucuucaA..............   16    1   bc3
............................................................ugucauggagucgcucucuucaC..............   3     1   bc3
............................................................ugucauggagucgcucucuucaU..............   104   1   bc3
............................................................ugucauggagucgcucucuucaga.............   1     0   bc3
............................................................ugucauggagucgcucucuucagC.............   1     1   bc3
.............................................................gucauggagGcgcucucuu.................   1     1   bc3
.............................................................gucauggagGcgcucucuuc................   1     1   bc3
.............................................................gucauggagGcgcucucuuca...............   8     1   bc3
.............................................................gucauggagucgcucucuuca...............   18    0   bc3
..............................................................ucauggagucgcucucuuca...............   2     0   bc3
...............................................................cauggagGcgcucucuuca...............   1     1   bc3
................................................................auggagucgcucucuuca...............   11    0   bc3
................................................................auggagGcgcucucuuca...............   3     1   bc3

.............Caagagagccgucuauugacagu.............................................................   5     1   bc6
..............aagagagccgucuauugU.................................................................   2     1   bc6
..............aagagagcAgucuauugacag..............................................................   102   1   bc6
..............aagagagccgucuauugacag..............................................................   166   0   bc6
..............aagagGgccgucuauugacag..............................................................   1     1   bc6
..............aagagagccgucuauuUacagu.............................................................   1     1   bc6
..............aagagagcGgucuauugacagu.............................................................   1     1   bc6
..............aagagagccgucuauuAacagu.............................................................   3     1   bc6
..............aagagagccgGcuauugacagu.............................................................   1     1   bc6
..............aaAagagccgucuauugacagu.............................................................   1     1   bc6
..............Gagagagccgucuauugacagu.............................................................   2     1   bc6
..............aagagagccgucuauugacGgu.............................................................   2     1   bc6
..............aagagagccgucuauugacagC.............................................................   1     1   bc6
..............aagagagUcgucuauugacagu.............................................................   1     1   bc6
..............aagagagccgucuauugacagu.............................................................   2869  0   bc6
..............aagagagccgucuauugaAagu.............................................................   2     1   bc6
..............aagagagccgucCauugacagu.............................................................   1     1   bc6
..............aagagagccguGuauugacagu.............................................................   1     1   bc6
..............aagaAagccgucuauugacagu.............................................................   1     1   bc6
..............aagagagccgucuauugCcagu.............................................................   1     1   bc6
..............aagagagcAgucuauugacagu.............................................................   2417  1   bc6
..............aagagagccgucuauugaUagu.............................................................   1     1   bc6
..............aagagagccgucuauugacagG.............................................................   7     1   bc6
..............aagCgagccgucuauugacagu.............................................................   1     1   bc6
..............aagagagccguUuauugacagu.............................................................   1     1   bc6
..............aagagagccgucuUuugacagu.............................................................   1     1   bc6
..............aagagagccgucuauugaGagu.............................................................   1     1   bc6
..............aagagCgccgucuauugacagu.............................................................   3     1   bc6
..............aagagGgccgucuauugacagu.............................................................   2     1   bc6
..............aagagagccgCcuauugacagu.............................................................   1     1   bc6
..............aagagagAcgucuauugacagu.............................................................   2     1   bc6
..............aagagagccgucuauCgacagu.............................................................   1     1   bc6
..............aagagagccgucuauugacCgu.............................................................   3     1   bc6
..............aagagagccgucuauugGcagu.............................................................   1     1   bc6
..............aagagagccgucuauugacagA.............................................................   1     1   bc6
..............aagagagccgucuauugacaguG............................................................   1     1   bc6
..............aagagagcAgucuauugacaguu............................................................   39    1   bc6
..............aagagagccgucuauugacaguu............................................................   40    0   bc6
..............aagagagccgucuauugacaguA............................................................   2     1   bc6
..............aagagagccgucuauugacaguuU...........................................................   2     1   bc6
...............agagagccgGcuauugacag..............................................................   1     1   bc6
...............agagagcAgucuauugacag..............................................................   1     1   bc6
...............agagagcAgucuauugacagu.............................................................   5     1   bc6
...............agagagccgucuauugacagu.............................................................   15    0   bc6
...............agagagccgucuauugacaguu............................................................   1     0   bc6
................gagagccgucuauugacag..............................................................   1     0   bc6
.................agagccgucuauugacagu.............................................................   1     0   bc6
..................gagccgucuauugacagG.............................................................   1     1   bc6
..................gagcAgucuauugacagu.............................................................   11    1   bc6
..................gagccgucuauugacagu.............................................................   6     0   bc6
....................................ucaagaccacgagucguugugugc.....................................   5     0   bc6
....................................ucaagaccacgagucguugugugcu....................................   6     0   bc6
....................................ucaagaccacgagucguugugGgcu....................................   1     1   bc6
```

```
              cel-miR-46*
                                                              cel-miR-46
cugaggugaagcugaagagagccgucuauugacaguucaagaccacgagucguugugugcugucauggagucgcucucuucagaugauccggucaau

....................................ucaagaccacgagucguugugugcuUu..................................   1       1    bc6
....................................ucaagaccacgagucguugugugcugucauggagucgcucucuuca...............   2       0    bc6
..........................................................gcugucauggagucgcucucuuca...............   1       0    bc6
...........................................................cugucauggagucgcucucuu.................   2       0    bc6
...........................................................cugucauggagucgcucucuucU...............   3       1    bc6
...........................................................cugucauggagucgcucucuuca...............   24      0    bc6
...........................................................cugucauggagGcgcucucuuca...............   4       1    bc6
............................................................ugucauggagGcgcucuc...................   8       1    bc6
............................................................ugucauggagucgcucuc...................   11      0    bc6
............................................................ugucauggagucgcucuU...................   2       1    bc6
............................................................ugucauggagucgcucucu..................   27      0    bc6
............................................................ugucauggagGcgcucucu..................   94      1    bc6
............................................................ugucauggagAcgcucucuu.................   2       1    bc6
............................................................ugucauggGgucgcucucuu.................   1       1    bc6
............................................................ugucauggaguGgcucucuu.................   1       1    bc6
............................................................ugucauggUgucgcucucuu.................   1       1    bc6
............................................................ugucauggagucgcucucuu.................   417     0    bc6
............................................................ugucauggagCcgcucucuu.................   3       1    bc6
............................................................ugucauggagGcgcucucuu.................   710     1    bc6
............................................................ugucauggagucgcucucuuc................   188     0    bc6
............................................................ugucauggagucgcucucuuU................   1       1    bc6
............................................................ugucauggagGcgcucucuuc................   383     1    bc6
............................................................ugucauggaguCcucucuuc.................   1       1    bc6
............................................................ugucauggagucgcucucuuca...............   15360   0    bc6
............................................................ugucauggGgucgcucucuuca...............   8       1    bc6
............................................................ugucaugUagucgcucucuuca...............   2       1    bc6
............................................................ugucauggagucgcAcucuuca...............   1       1    bc6
............................................................ugucauggagucgUucucuuca...............   5       1    bc6
............................................................ugucauggaguUgcucucuuca...............   8       1    bc6
............................................................ugucauggagucgGucucuuca...............   3       1    bc6
............................................................ugucauggagucgcCcucuuca...............   3       1    bc6
............................................................Cgucauggagucgcucucuuca...............   9       1    bc6
............................................................uguGauggagucgcucucuuca...............   4       1    bc6
............................................................ugucauggagucgcucucuucG...............   45      1    bc6
............................................................ugucauggagucgcucucuuUa...............   3       1    bc6
............................................................ugucauggagucgcGcucuuca...............   2       1    bc6
............................................................ugCcauggagucgcucucuuca...............   3       1    bc6
............................................................ugucauggagCcgcucucuuca...............   26      1    bc6
............................................................ugucauUgagucgcucucuuca...............   6       1    bc6
............................................................ugucauggagucgcuAucuuca...............   1       1    bc6
............................................................Ggucauggagucgcucucuuca...............   2       1    bc6
............................................................ugucCuggagucgcucucuuca...............   1       1    bc6
............................................................ugucaugAagucgcucucuuca...............   7       1    bc6
............................................................uguAauggagucgcucucuuca...............   1       1    bc6
............................................................ugucauggaCucgcucucuuca...............   3       1    bc6
............................................................ugucauggUgucgcucucuuca...............   7       1    bc6
............................................................ugucGuggagucgcucucuuca...............   7       1    bc6
............................................................uAucauggagucgcucucuuca...............   4       1    bc6
............................................................ugucauggagucgcucucuCca...............   32      1    bc6
............................................................ugucauggagucAcucucuuca...............   4       1    bc6
............................................................ugAcauggagucgcucucuuca...............   5       1    bc6
............................................................ugucauggCgucgcucucuuca...............   7       1    bc6
............................................................ugucauggagucgcuGucuuca...............   1       1    bc6
............................................................ugucauggaguCcucucuuca...............   2       1    bc6
............................................................ugucaCggagucgcucucuuca...............   4       1    bc6
............................................................ugucauggagucgcucuUuuca...............   7       1    bc6
............................................................ugucauggagucgcucucuucC...............   36      1    bc6
............................................................ugucUuggagucgcucucuuca...............   5       1    bc6
............................................................ugucauggaguGgcucucuuca...............   5       1    bc6
............................................................ugucauAgagucgcucucuuca...............   4       1    bc6
............................................................ugucauggaUucgcucucuuca...............   1       1    bc6
............................................................ugucauggagucgcucucuucU...............   1416    1    bc6
............................................................ugucauggagucgcuUucuuca...............   4       1    bc6
............................................................ugucauggagucgcucuCuca...............   3       1    bc6
............................................................ugucauCgagucgcucucuuca...............   3       1    bc6
............................................................ugucauggagGcgcucucuuca...............   5359    1    bc6
............................................................ugucaugCagucgcucucuuca...............   1       1    bc6
............................................................ugucauggagucgcucCcuuca...............   4       1    bc6
............................................................uguUauggagucgcucucuuca...............   4       1    bc6
```

```
               cel-miR-46*
                                                            cel-miR-46
cugaggugaagcugaagagagccgucuauugacaguucaagaccacgagucguugugugcugucauggagucgcucucuucagaugauccggucaau

............................................................ugucauggagucgcucucuAca...............    14     1     bc6
............................................................ugucauggagucgcucucuuAa...............    6      1     bc6
............................................................ugucauggaAucgcucucuuca...............    5      1     bc6
............................................................Agucauggagucgcucucuuca...............    3      1     bc6
............................................................ugucauggaguAgcucucuuca...............    6      1     bc6
............................................................ugucauggagucgcucucuGca...............    8      1     bc6
............................................................ugGcauggagucgcucucuuca...............    3      1     bc6
............................................................ugucauggagAcgcucucuuca...............    14     1     bc6
............................................................ugucauggagucgcucucuucag..............    4      0     bc6
............................................................ugucauggagucgcucucuucaU..............    117    1     bc6
............................................................ugucauggagucgcucucuucaC..............    3      1     bc6
............................................................ugucauggagucgcucucuucaA..............    20     1     bc6
.............................................................gucauggagGcgcucucuu.................    3      1     bc6
.............................................................gucauggagGcgcucucuuca...............    10     1     bc6
.............................................................gucauggagucgcucucuuca...............    31     0     bc6
.............................................................gucauggagucgcucucuucU...............    3      1     bc6
..............................................................ucauggagGcgcucucuuca...............    1      1     bc6
..............................................................ucauggagucgcucucuuca...............    1      0     bc6
................................................................auggagucgcucucuuca...............    19     0     bc6
................................................................auggagucgcucucuucC...............    1      1     bc6
................................................................auggagGcgcucucuuca...............    14     1     bc6
................................................................auggagucgcucucuucU...............    1      1     bc6

.............Caagagagccgucuauugacagu.............................................................    2      1     bc5
.............Caagagagccgucuauugacaguu............................................................    1      1     bc5
..............aagagagcAgucuauugac................................................................    1      1     bc5
..............aagagagccgucuauugac................................................................    1      0     bc5
..............aagagagcAgucuauugaca...............................................................    2      1     bc5
..............aagagagccgucuauugacag..............................................................    265    0     bc5
..............aagagagcAgucuauugacag..............................................................    136    1     bc5
..............aagagagccgucuauugacaA..............................................................    1      1     bc5
..............aagaAagccgucuauugacagu.............................................................    1      1     bc5
..............aagagagccgucuaCugacagu.............................................................    1      1     bc5
..............aagagaCccgucuauugacagu.............................................................    1      1     bc5
..............aagagagccgucuauugacagC.............................................................    2      1     bc5
..............aagagagccgucuauugaGagu.............................................................    1      1     bc5
..............aagagagccgucuauugacGgu.............................................................    3      1     bc5
..............aagagagccgucuauuAacagu.............................................................    2      1     bc5
..............aagaUagccgucuauugacagu.............................................................    1      1     bc5
..............aagagagccgucuauugaUagu.............................................................    2      1     bc5
..............aagagagcUgucuauugacagu.............................................................    1      1     bc5
..............aagagagccgucuauugacUgu.............................................................    1      1     bc5
..............aagagagccAucuauugacagu.............................................................    1      1     bc5
..............aagagagAcgucuauugacagu.............................................................    1      1     bc5
..............aagagagcAgucuauugacagu.............................................................    2526   1     bc5
..............aagagagccgCcuauugacagu.............................................................    1      1     bc5
..............aagagagccgucuGuugacagu.............................................................    1      1     bc5
..............aagagagccgucuauugacaCu.............................................................    1      1     bc5
..............aagagagccUucuauugacagu.............................................................    1      1     bc5
..............aagagagccgucuUuugacagu.............................................................    1      1     bc5
..............aagagagccgucuauugUcagu.............................................................    1      1     bc5
..............aagagagccgucuaAugacagu.............................................................    3      1     bc5
..............aagagCgccgucuauugacagu.............................................................    2      1     bc5
..............aagagagccgAcuauugacagu.............................................................    1      1     bc5
..............aagGgagccgucuauugacagu.............................................................    1      1     bc5
..............aagagagUcgucuauugacagu.............................................................    3      1     bc5
..............aagagagccgucuauugacagG.............................................................    8      1     bc5
..............aagagagccguUuauugacagu.............................................................    1      1     bc5
..............aagagagccgucuauugacCgu.............................................................    2      1     bc5
..............aagagagccgucuauCgacagu.............................................................    1      1     bc5
..............aagagaUccgucuauugacagu.............................................................    2      1     bc5
..............aagagagccgGcuauugacagu.............................................................    1      1     bc5
..............Uagagagccgucuauugacagu.............................................................    1      1     bc5
..............aagagagccgucuauugacagu.............................................................    3534   0     bc5
..............aagagagccgucuaGugacagu.............................................................    1      1     bc5
..............aagagagccgucuauugacaguG............................................................    2      1     bc5
..............aagagagcAgucuauugacaguu............................................................    36     1     bc5
..............aagagagccgucuauugacaguu............................................................    53     0     bc5
..............aagagagccgucuauugacaguuG...........................................................    3      1     bc5
```

```
              cel-miR-46*
                                              cel-miR-46
cugaggugaagcugaagagagccgucuauugacaguucaagaccacgagucguugugugcugucauggagucgcucucuucagaugauccggucaau

..............aagagagccgucuauugacaguuU...........................................................    3      1    bc5
...............agagagccgucuauugacag..............................................................    1      0    bc5
...............agagagcAgucuauugacagu.............................................................    3      1    bc5
...............agagagccgucuauugacagu.............................................................    14     0    bc5
...............agagagcAgucuauugacaguu............................................................    1      1    bc5
...............agagagccgucuauugacaguu............................................................    2      0    bc5
................Aagagccgucuauugacagu.............................................................    1      1    bc5
.................agagcAgucuauugacagu.............................................................    2      1    bc5
..................gagcAgucuauugacagu.............................................................    12     1    bc5
..................gagccgucuauugacagu.............................................................    17     0    bc5
....................................ucaagaccacgagucguugugugc.....................................    7      0    bc5
....................................ucaagaccacgagucguugugugcu....................................    14     0    bc5
....................................ucaagaccacgagucguugugugcC....................................    1      1    bc5
....................................ucaagaccacgagucguugugugcuU...................................    1      1    bc5
....................................ucaagaccacgagucguugugugcuguca................................    1      0    bc5
..........................................ccacgagucguugugugc.....................................    1      0    bc5
.........................................................ugcugucauggagucgcucucuuca...............    1      0    bc5
..........................................................gcugucauggagucgcucucuuca...............    2      0    bc5
...........................................................cugucauggagucgcucucuu.................    2      0    bc5
...........................................................cugucauggagucgcucucuuc................    1      0    bc5
...........................................................cugucauggagGcgcucucuuc................    1      1    bc5
...........................................................cugucauggagGcgcucucuuca...............    5      1    bc5
...........................................................cugucauggagucgcucucuucU...............    1      1    bc5
...........................................................cugucauggagucgcucucuuca...............    29     0    bc5
...........................................................cugucauggagucgcucucuucaU..............    1      1    bc5
............................................................ugucauggagucgcucuU...................    1      1    bc5
............................................................ugucauggagGcgcucuc...................    10     1    bc5
............................................................ugucauggagucgcucuc...................    13     0    bc5
............................................................ugucauggagucgcucucu..................    41     0    bc5
............................................................ugucauggagGcgcucucu..................    157    1    bc5
............................................................ugucauggagucgcucucuG.................    1      1    bc5
............................................................ugucauggagucgcucucuA.................    1      1    bc5
............................................................ugucauggagucgcucucuu.................    662    0    bc5
............................................................ugucauggagucgcCcucuu.................    1      1    bc5
............................................................ugucauggagucgcucuGuu.................    1      1    bc5
............................................................ugucauggagCcgcucucuu.................    1      1    bc5
............................................................ugucauggagucgcucucuC.................    1      1    bc5
............................................................ugucauggagucgcucuUuu.................    1      1    bc5
............................................................ugucaugCagucgcucucuu.................    1      1    bc5
............................................................ugucauggagGcgcucucuu.................    958    1    bc5
............................................................ugucauggagucgcucucuuc................    290    0    bc5
............................................................ugucauggagucgcucuAuuc................    1      1    bc5
............................................................ugucauggagucgcucucuuA................    1      1    bc5
............................................................ugucauggagucgcucucuAc................    1      1    bc5
............................................................ugucauggagGcgcucucuuc................    552    1    bc5
............................................................ugCcauggagucgcucucuuc................    1      1    bc5
............................................................ugucauggagAcgcucucuuc................    1      1    bc5
............................................................ugucauggagucgcucucuuU................    2      1    bc5
............................................................ugucauggagucgcCcucuuca...............    4      1    bc5
............................................................ugucauggagucgcucucuucC...............    55     1    bc5
............................................................ugucGuggagucgcucucuuca...............    2      1    bc5
............................................................ugucauggagucgcucuUuuca...............    7      1    bc5
............................................................ugucaugAagucgcucucuuca...............    2      1    bc5
............................................................ugucauggagGcgcucucuuca...............    7365   1    bc5
............................................................ugucauAgagucgcucucuuca...............    1      1    bc5
............................................................ugucauggagucgcucucuuca...............    20732  0    bc5
............................................................ugucauggGgucgcucucuuca...............    6      1    bc5
............................................................ugucauggagucgAucucuuca...............    3      1    bc5
............................................................ugucauggagucgcucuGuuca...............    2      1    bc5
............................................................Agucauggagucgcucucuuca...............    2      1    bc5
............................................................Cgucauggagucgcucucuuca...............    4      1    bc5
............................................................ugucauggagucgcucucuAca...............    23     1    bc5
............................................................ugucauggagucgcucucuGca...............    3      1    bc5
............................................................uguAauggagucgcucucuuca...............    3      1    bc5
............................................................ugAcauggagucgcucucuuca...............    2      1    bc5
............................................................ugucauggagucAcucucuuca...............    9      1    bc5
............................................................ugucauggagucgcucucuuAa...............    7      1    bc5
............................................................ugucaGggagucgcucucuuca...............    4      1    bc5
............................................................ugucauUgagucgcucucuuca...............    3      1    bc5
```

```
              cel-miR-46*
                                              cel-miR-46
cugaggugaagcugaagagagccgucuauugacaguucaagaccacgagucguugugugcugucauggagucgcucucuucagaugauccggucaau

............................................................ugucUuggagucgcucucuuca...............   1     1   bc5
............................................................uUucauggagucgcucucuuca...............   1     1   bc5
............................................................ugucauggCgucgcucucuuca...............   2     1   bc5
............................................................ugucauggaguAgcucucuuca...............   5     1   bc5
............................................................uguGauggagucgcucucuuca...............   4     1   bc5
............................................................ugucauggagucgcuUucuuca...............   4     1   bc5
............................................................ugucauggagucgcuAucuuca...............   5     1   bc5
............................................................ugucauggagucgcucCcuuca...............   3     1   bc5
............................................................ugucauggagucCcucucuuca...............   1     1   bc5
............................................................ugucauggagucgcucucAuca...............   1     1   bc5
............................................................ugucauggaguUgcucucuuca...............   3     1   bc5
............................................................ugucauggagucgcucucuucG...............   83    1   bc5
............................................................ugucauggagucgcucucCuca...............   4     1   bc5
............................................................ugucauggagucgcucucuCca...............   32    1   bc5
............................................................ugucauggagucgcucucuuGa...............   1     1   bc5
............................................................ugucaCggagucgcucucuuca...............   4     1   bc5
............................................................ugucCuggagucgcucucuuca...............   4     1   bc5
............................................................ugucaugCagucgcucucuuca...............   5     1   bc5
............................................................ugucauggagucgGucucuuca...............   5     1   bc5
............................................................uguUauggagucgcucucuuca...............   2     1   bc5
............................................................Ggucauggagucgcucucuuca...............   2     1   bc5
............................................................ugucauggaUucgcucucuuca...............   2     1   bc5
............................................................ugucauggagucgcucuAuuca...............   4     1   bc5
............................................................ugucauggaguGgcucucuuca...............   3     1   bc5
............................................................ugucauggagCcgcucucuuca...............   14    1   bc5
............................................................ugGcauggagucgcucucuuca...............   6     1   bc5
............................................................ugucauggUgucgcucucuuca...............   5     1   bc5
............................................................uCucauggagucgcucucuuca...............   2     1   bc5
............................................................ugucaugUagucgcucucuuca...............   1     1   bc5
............................................................ugucauggagucgUucucuuca...............   5     1   bc5
............................................................ugCcauggagucgcucucuuca...............   2     1   bc5
............................................................ugucauggagucgcucucuucU...............   1952  1   bc5
............................................................ugucauggaAucgcucucuuca...............   2     1   bc5
............................................................ugucauggagucgcucucuuUa...............   10    1   bc5
............................................................ugucauggagucUcucucuuca...............   2     1   bc5
............................................................ugucauggaCucgcucucuuca...............   5     1   bc5
............................................................uAucauggagucgcucucuuca...............   5     1   bc5
............................................................ugucauggagAcgcucucuuca...............   9     1   bc5
............................................................ugucauggagucgcucucuucaU..............   189   1   bc5
............................................................ugucauggagucgcucucuucaA..............   21    1   bc5
............................................................ugucauggagucgcucucuucaC..............   3     1   bc5
............................................................ugucauggagGcgcucucuucag..............   2     1   bc5
............................................................ugucauggagucgcucucuucag..............   4     0   bc5
............................................................ugucauggagucgcucucuucaCa.............   2     1   bc5
............................................................ugucauggagucgcucucuucaAa.............   2     1   bc5
............................................................ugucauggagucgcucucuucaga.............   1     0   bc5
.............................................................gucauggagGcgcucucuu.................   1     1   bc5
.............................................................gucauggagucgcucucuuca...............   30    0   bc5
.............................................................gucauggagucgcucucuucU...............   1     1   bc5
.............................................................gucauggaguUgcucucuuca...............   1     1   bc5
.............................................................gucauggagGcgcucucuuca...............   14    1   bc5
..............................................................ucauggagucgcucucuucU...............   1     1   bc5
..............................................................ucauggagucgcucucuuca...............   4     0   bc5
...............................................................cauggagGcgcucucuuca...............   2     1   bc5
................................................................auggagucgcucucuuca...............   22    0   bc5
................................................................auggagGcgcucucuuca...............   12    1   bc5

............uCaagagagccgucuauugacagu.............................................................   1     1   bc2
.............Caagagagccgucuauugacagu.............................................................   1     1   bc2
..............aagagagccgucuauugaca...............................................................   1     0   bc2
..............aagagagccgucuauugacG...............................................................   1     1   bc2
..............aagagagccguGuauugacag..............................................................   1     1   bc2
..............aagagagcUgucuauugacag..............................................................   1     1   bc2
..............aagagagccgucuauugacag..............................................................   92    0   bc2
..............aagagagcAgucuauugacag..............................................................   33    1   bc2
..............aagagagccgucuauugUcagu.............................................................   1     1   bc2
..............aagagUgccgucuauugacagu.............................................................   1     1   bc2
..............aagagagccgucuauugacagA.............................................................   1     1   bc2
..............aagagagccgucuauuUacagu.............................................................   2     1   bc2
```

```
               cel-miR-46*
                                                            cel-miR-46
cugaggugaagcugaagagagccgucuauugacaguucaagaccacgagucguugugugcugucauggagucgcucucuucagaugauccggucaau

..............aagagagccUucuauugacagu.............................................................    1      1    bc2
..............aagagagccgucuGuugacagu.............................................................    2      1    bc2
..............aagagagccgucuauugCcagu.............................................................    1      1    bc2
..............aagagaUccgucuauugacagu.............................................................    1      1    bc2
..............aagagagUcgucuauugacagu.............................................................    1      1    bc2
..............aaCagagccgucuauugacagu.............................................................    1      1    bc2
..............aagagagccgucuauugacCgu.............................................................    2      1    bc2
..............Gagagagccgucuauugacagu.............................................................    1      1    bc2
..............aagagagccguUuauugacagu.............................................................    1      1    bc2
..............aagagagccgucuauugacagG.............................................................    3      1    bc2
..............aagagagccgucuauugaUagu.............................................................    1      1    bc2
..............aagagagccgucuauugacagu.............................................................    1397   0    bc2
..............aGgagagccgucuauugacagu.............................................................    1      1    bc2
..............aagagagccguAuauugacagu.............................................................    1      1    bc2
..............aagagagccgucuauugacGgu.............................................................    3      1    bc2
..............aagagagcAgucuauugacagu.............................................................    786    1    bc2
..............aagagagccgucuauugacaCu.............................................................    1      1    bc2
..............aagaUagccgucuauugacagu.............................................................    1      1    bc2
..............aagagagccgucuauugacaguu............................................................    22     0    bc2
..............aagagagcAgucuauugacaguu............................................................    13     1    bc2
..............aagagagccgucuauugacaguuU...........................................................    3      1    bc2
...............agagagccgucuauugacagu.............................................................    2      0    bc2
...............agagagccgucuauugacaguu............................................................    1      0    bc2
.................agagccgucuauugacagu.............................................................    1      0    bc2
..................gagcAgucuauugacagu.............................................................    4      1    bc2
..................gagccgucuauugacagu.............................................................    6      0    bc2
....................................ucaagaccacgagucguugugugc.....................................    1      0    bc2
....................................ucaagaccacgagucguugugugcu....................................    4      0    bc2
..........................................................gcugucauggagucgcucucuuca...............    1      0    bc2
...........................................................cugucauggagGcgcucucuu.................    2      1    bc2
...........................................................cugucauggagucgcucucuuc................    1      0    bc2
...........................................................cugucauggagucgcucucuuca...............    16     0    bc2
...........................................................cugucauggagGcgcucucuuca...............    3      1    bc2
...........................................................Augucauggagucgcucucuuca...............    1      1    bc2
...........................................................cugucauggagucgcucucuucU...............    3      1    bc2
............................................................ugucauggagucgcucuc...................    11     0    bc2
............................................................ugucauggagGcgcucuc...................    9      1    bc2
............................................................ugucauggagucgcucuU...................    3      1    bc2
............................................................ugucauggagucgcucucA..................    1      1    bc2
............................................................ugucauggagucgcucucu..................    17     0    bc2
............................................................uguAauggagucgcucucu..................    1      1    bc2
............................................................ugucauggagGcgcucucu..................    44     1    bc2
............................................................ugucauggagucgcucucuu.................    302    0    bc2
............................................................ugucauggagGcgcucucuu.................    391    1    bc2
............................................................Cgucauggagucgcucucuu.................    1      1    bc2
............................................................ugucauUgagucgcucucuu.................    1      1    bc2
............................................................ugucauggagucgcucucuuc................    151    0    bc2
............................................................ugGcauggagucgcucucuuc................    1      1    bc2
............................................................ugucauggagGcgcucucuuc................    256    1    bc2
............................................................ugucauggagCcgcucucuuc................    1      1    bc2
............................................................ugucauggagucgcucucuuU................    1      1    bc2
............................................................ugucauggaguAgcucucuuc................    1      1    bc2
............................................................ugucauggagucgcucuAuuc................    1      1    bc2
............................................................ugucauggagucgcucucuCca...............    11     1    bc2
............................................................uAucauggagucgcucucuuca...............    4      1    bc2
............................................................ugucauggagucgcucucuuca...............    9246   0    bc2
............................................................ugucGuggagucgcucucuuca...............    6      1    bc2
............................................................ugucaCggagucgcucucuuca...............    4      1    bc2
............................................................ugucauggagucgcucucuuUa...............    3      1    bc2
............................................................ugucauggagucgcucucuucG...............    17     1    bc2
............................................................ugucauggagucgcucucCuca...............    2      1    bc2
............................................................ugucauggagucgcucuUuuca...............    4      1    bc2
............................................................ugucauggagucgcuUucuuca...............    4      1    bc2
............................................................ugucauggagucgcucucuucC...............    26     1    bc2
............................................................ugucauggaguUgcucucuuca...............    1      1    bc2
............................................................uguAauggagucgcucucuuca...............    3      1    bc2
............................................................ugucauggaguGgcucucuuca...............    2      1    bc2
............................................................ugucauggagucAcucucuuca...............    7      1    bc2
............................................................ugucauggaguAgcucucuuca...............    6      1    bc2
```

```
              cel-miR-46*
                                                            cel-miR-46
cugaggugaagcugaagagagccgucuauugacaguucaagaccacgagucguugugugcugucauggagucgcucucuucagaugauccggucaau

............................................................ugucauAgagucgcucucuuca...............    1       1       bc2
............................................................ugucauggaAucgcucucuuca...............    1       1       bc2
............................................................ugucauggagucgUucucuuca...............    3       1       bc2
............................................................ugucauggGgucgcucucuuca...............    8       1       bc2
............................................................ugucauggagucgcucucuAca...............    9       1       bc2
............................................................ugucauggagucgcucucuuGa...............    1       1       bc2
............................................................ugucauggagCcgcucucuuca...............    17      1       bc2
............................................................ugucauggUgucgcucucuuca...............    8       1       bc2
............................................................ugucauggagGcgcucucuuca...............    3268    1       bc2
............................................................ugucauggagucgGucucuuca...............    1       1       bc2
............................................................Ggucauggagucgcucucuuca...............    1       1       bc2
............................................................ugCcauggagucgcucucuuca...............    2       1       bc2
............................................................ugucauggagucgAucucuuca...............    4       1       bc2
............................................................ugucauggaUucgcucucuuca...............    6       1       bc2
............................................................ugucauggagucgcuAucuuca...............    3       1       bc2
............................................................ugucauggagucgcucCcuuca...............    2       1       bc2
............................................................ugucauggagucUcucucuuca...............    2       1       bc2
............................................................ugucauggagucgcucuAuuca...............    2       1       bc2
............................................................Agucauggagucgcucucuuca...............    3       1       bc2
............................................................ugucauggaCucgcucucuuca...............    2       1       bc2
............................................................ugucauggagAcgcucucuuca...............    9       1       bc2
............................................................uguUauggagucgcucucuuca...............    4       1       bc2
............................................................uguGauggagucgcucucuuca...............    2       1       bc2
............................................................Cgucauggagucgcucucuuca...............    3       1       bc2
............................................................ugucauggagucgcucucuGca...............    4       1       bc2
............................................................ugucCuggagucgcucucuuca...............    1       1       bc2
............................................................ugucauggagucgcucuGuuca...............    1       1       bc2
............................................................ugucauggagucgcuGucuuca...............    1       1       bc2
............................................................ugucauggagucgcCcucuuca...............    5       1       bc2
............................................................ugucauCgagucgcucucuuca...............    1       1       bc2
............................................................ugucauggCgucgcucucuuca...............    2       1       bc2
............................................................ugucauggagucgcucucuuAa...............    2       1       bc2
............................................................ugucauggagucgcucucuucU...............    682     1       bc2
............................................................ugucauggagucgcucucuucag..............    1       0       bc2
............................................................ugucauggagucgcucucuucaA..............    13      1       bc2
............................................................ugucauggagucgcucucuucaU..............    90      1       bc2
.............................................................Uucauggagucgcucucuuca...............    1       1       bc2
.............................................................gucauggagucgcucucuucU...............    1       1       bc2
.............................................................gucauggagucgcucucuuca...............    16      0       bc2
.............................................................gucauggagGcgcucucuuca...............    10      1       bc2
...............................................................cauggagucgcucucuuca...............    1       0       bc2
................................................................auggagGcgcucucuuca...............    3       1       bc2
................................................................auggagucgcucucuuca...............    12      0       bc2

.............Caagagagccgucuauugacagu.............................................................    4       1       bc7
..............aagagagccgucuauugac................................................................    3       0       bc7
..............aagagagcAgucuauugaca...............................................................    1       1       bc7
..............aagagagccgCcuauugacag..............................................................    1       1       bc7
..............aagagagccgucuauugacag..............................................................    226     0       bc7
..............aagGgagccgucuauugacag..............................................................    1       1       bc7
..............aagagagcAgucuauugacag..............................................................    122     1       bc7
..............aagagagccgucuauuAacagu.............................................................    2       1       bc7
..............aagagagcUgucuauugacagu.............................................................    2       1       bc7
..............aagagagccgucuauugaAagu.............................................................    2       1       bc7
..............aUgagagccgucuauugacagu.............................................................    1       1       bc7
..............aagagagccguGuauugacagu.............................................................    1       1       bc7
..............aagaAagccgucuauugacagu.............................................................    2       1       bc7
..............aagagagccgucuauugGcagu.............................................................    1       1       bc7
..............aagagagccgAcuauugacagu.............................................................    1       1       bc7
..............aagagagccgucCauugacagu.............................................................    1       1       bc7
..............aagagagccgGcuauugacagu.............................................................    3       1       bc7
..............aagagagccguUuauugacagu.............................................................    2       1       bc7
..............aagagagccgCcuauugacagu.............................................................    5       1       bc7
..............aagagagccgucuauugacGgu.............................................................    3       1       bc7
..............aaAagagccgucuauugacagu.............................................................    1       1       bc7
..............aGgagagccgucuauugacagu.............................................................    1       1       bc7
..............aagagagAcgucuauugacagu.............................................................    4       1       bc7
..............aagagagcGgucuauugacagu.............................................................    1       1       bc7
..............aagagagccgucuauAgacagu.............................................................    1       1       bc7
```

```
              cel-miR-46*
                                                            cel-miR-46
cugaggugaagcugaagagagccgucuauugacaguucaagaccacgagucguugugugcugucauggagucgcucucuucagaugauccggucaau

..............aagagagccgucuauugacagG.............................................................    13      1    bc7
..............aagagaCccgucuauugacagu.............................................................    2       1    bc7
..............aagagagccgucuauuUacagu.............................................................    1       1    bc7
..............aagagagccgucuauugacagC.............................................................    1       1    bc7
..............aagagaUccgucuauugacagu.............................................................    1       1    bc7
..............aagGgagccgucuauugacagu.............................................................    1       1    bc7
..............aagagagccgucuauugacagu.............................................................    3345    0    bc7
..............aagagagccguAuauugacagu.............................................................    1       1    bc7
..............aagagagccgucuauugacUgu.............................................................    2       1    bc7
..............aagagagcAgucuauugacagu.............................................................    2338    1    bc7
..............aagagCgccgucuauugacagu.............................................................    1       1    bc7
..............aagagagUcgucuauugacagu.............................................................    1       1    bc7
..............aagagagccCucuauugacagu.............................................................    1       1    bc7
..............aagagagccgucuauugacCgu.............................................................    4       1    bc7
..............Gagagagccgucuauugacagu.............................................................    3       1    bc7
..............aagagUgccgucuauugacagu.............................................................    1       1    bc7
..............aagagGgccgucuauugacagu.............................................................    1       1    bc7
..............aagagagccgucuauugacaguG............................................................    3       1    bc7
..............aagagagcAgucuauugacaguu............................................................    25      1    bc7
..............aagagagccgucuauugacaguu............................................................    31      0    bc7
..............aagagagccgucuauugacaguuU...........................................................    1       1    bc7
..............aagagagccgucuauugacaguuG...........................................................    1       1    bc7
...............agagagccgucuauugacag..............................................................    4       0    bc7
...............agagagccgucuauugacagu.............................................................    22      0    bc7
...............agagGgccgucuauugacagu.............................................................    1       1    bc7
...............agagagccgucuauugacaguu............................................................    2       0    bc7
.................agagccgucuauugacagu.............................................................    1       0    bc7
..................gagcAgucuauugacagu.............................................................    11      1    bc7
..................gagccgucuauugacagu.............................................................    14      0    bc7
....................................ucaagaccacgagucguugugug......................................    1       0    bc7
....................................ucaagaccacgagucguugugugc.....................................    4       0    bc7
....................................ucaagaccacgagucguugugugcu....................................    7       0    bc7
..........................................................gcugucauggagucgcucucuuc................    1       0    bc7
...........................................................cugucauggagucgcucucuu.................    2       0    bc7
...........................................................cugucauggagGcgcucucuu.................    3       1    bc7
...........................................................cugucauggagucgcucucuuca...............    19      0    bc7
...........................................................cugucauggagucgcucucuucU...............    4       1    bc7
...........................................................cugucauggagGcgcucucuuca...............    3       1    bc7
............................................................ugucauggagGcgcucuc...................    14      1    bc7
............................................................ugucauggagucgcucuc...................    19      0    bc7
............................................................ugucauggagucgcucucu..................    66      0    bc7
............................................................ugucauggaAucgcucucu..................    1       1    bc7
............................................................ugucauggagGcgcucucu..................    198     1    bc7
............................................................ugucauggagucgcucuUu..................    1       1    bc7
............................................................ugucauggagGcgcucucuu.................    1378    1    bc7
............................................................ugucUuggagucgcucucuu.................    1       1    bc7
............................................................ugucauUgagucgcucucuu.................    1       1    bc7
............................................................ugAcauggagucgcucucuu.................    1       1    bc7
............................................................ugucauggagucgcucucuG.................    4       1    bc7
............................................................ugucauggagucUcucucuu.................    1       1    bc7
............................................................ugucauggagucgcucucuu.................    819     0    bc7
............................................................ugucauggaAucgcucucuu.................    1       1    bc7
............................................................ugucauggagAcgcucucuu.................    1       1    bc7
............................................................ugucauggagucgcucucuA.................    1       1    bc7
............................................................ugucauggGgucgcucucuu.................    3       1    bc7
............................................................ugCcauggagucgcucucuu.................    1       1    bc7
............................................................ugucauggagucgcucucGu.................    1       1    bc7
............................................................ugucauggaguUgcucucuu.................    1       1    bc7
............................................................ugucauggaCucgcucucuu.................    1       1    bc7
............................................................ugucauggUgucgcucucuu.................    5       1    bc7
............................................................ugucauggagCcgcucucuu.................    6       1    bc7
............................................................ugucauggagGcgcucucuuc................    579     1    bc7
............................................................ugucauggGgucgcucucuuc................    1       1    bc7
............................................................Ggucauggagucgcucucuuc................    1       1    bc7
............................................................ugucauggagucgcucucuuA................    1       1    bc7
............................................................ugucauggagucgcucucuAc................    1       1    bc7
............................................................ugucauggagucgcucucuuc................    349     0    bc7
............................................................uguUauggagucgcucucuuc................    1       1    bc7
............................................................ugucauggagucgcucucuuU................    1       1    bc7
```

```
              cel-miR-46*
                                                            cel-miR-46
cugaggugaagcugaagagagccgucuauugacaguucaagaccacgagucguugugugcugucauggagucgcucucuucagaugauccggucaau

............................................................ugucauggagucAcucucuuca...............    16      1     bc7
............................................................ugGcauggagucgcucucuuca...............    5       1     bc7
............................................................ugucauggagucgcucucGuca...............    1       1     bc7
............................................................ugucauggaguAgcucucuuca...............    6       1     bc7
............................................................ugucUuggagucgcucucuuca...............    2       1     bc7
............................................................ugucauggagucgcGcucuuca...............    2       1     bc7
............................................................ugucauggagucgcucucCuca...............    2       1     bc7
............................................................uUucauggagucgcucucuuca...............    2       1     bc7
............................................................uguAauggagucgcucucuuca...............    1       1     bc7
............................................................ugucGuggagucgcucucuuca...............    8       1     bc7
............................................................ugucaugAagucgcucucuuca...............    2       1     bc7
............................................................ugucauggagucgcucucuucG...............    55      1     bc7
............................................................ugucauggCgucgcucucuuca...............    3       1     bc7
............................................................ugucauggagucgcucCcuuca...............    4       1     bc7
............................................................ugucauggagucUcucucuuca...............    1       1     bc7
............................................................Agucauggagucgcucucuuca...............    4       1     bc7
............................................................uCucauggagucgcucucuuca...............    3       1     bc7
............................................................uguGauggagucgcucucuuca...............    4       1     bc7
............................................................ugucauggagucgcucAcuuca...............    3       1     bc7
............................................................Cgucauggagucgcucucuuca...............    8       1     bc7
............................................................ugucauUgagucgcucucuuca...............    2       1     bc7
............................................................ugucauggagucgcucucuucC...............    52      1     bc7
............................................................ugucaAggagucgcucucuuca...............    1       1     bc7
............................................................ugucauggagucgcucucuucU...............    1939    1     bc7
............................................................ugucaugUagucgcucucuuca...............    2       1     bc7
............................................................ugucaCggagucgcucucuuca...............    3       1     bc7
............................................................ugucauggagucgcucucuAca...............    26      1     bc7
............................................................ugucauggaguGgcucucuuca...............    4       1     bc7
............................................................ugucaGggagucgcucucuuca...............    2       1     bc7
............................................................ugucauggaCucgcucucuuca...............    3       1     bc7
............................................................ugucauggagucgcucucuuUa...............    6       1     bc7
............................................................ugucauggagucgGucucuuca...............    2       1     bc7
............................................................ugucauggagCcgcucucuuca...............    49      1     bc7
............................................................ugucauggagucgcucuUuuca...............    15      1     bc7
............................................................ugucauggagucgcuAucuuca...............    4       1     bc7
............................................................ugucauggagucgcucucuuca...............    19121   0     bc7
............................................................ugucauggaUucgcucucuuca...............    5       1     bc7
............................................................ugucCuggagucgcucucuuca...............    1       1     bc7
............................................................ugucauggagucgcucucuGca...............    14      1     bc7
............................................................ugucauAgagucgcucucuuca...............    8       1     bc7
............................................................ugCcauggagucgcucucuuca...............    4       1     bc7
............................................................ugucauggagucgcucucuCca...............    39      1     bc7
............................................................ugucauggUgucgcucucuuca...............    15      1     bc7
............................................................ugucaugCagucgcucucuuca...............    2       1     bc7
............................................................ugucauggagucgAucucuuca...............    6       1     bc7
............................................................ugucauggagucgcucuAuuca...............    7       1     bc7
............................................................ugucauggagucgUucucuuca...............    4       1     bc7
............................................................uguUauggagucgcucucuuca...............    6       1     bc7
............................................................ugucauggagucgcuGucuuca...............    2       1     bc7
............................................................ugAcauggagucgcucucuuca...............    6       1     bc7
............................................................ugucauggaguUgcucucuuca...............    9       1     bc7
............................................................ugucauggagAcgcucucuuca...............    16      1     bc7
............................................................uAucauggagucgcucucuuca...............    2       1     bc7
............................................................ugucauggagucgcucucuuGa...............    1       1     bc7
............................................................ugucauggagGcgcucucuuca...............    6267    1     bc7
............................................................ugucauggGgucgcucucuuca...............    14      1     bc7
............................................................ugucauggagucgcucucuuAa...............    2       1     bc7
............................................................ugucauggagucgcucucAuca...............    1       1     bc7
............................................................ugucauggagucgcCcucuuca...............    4       1     bc7
............................................................ugucauggagucgcuUucuuca...............    7       1     bc7
............................................................Ggucauggagucgcucucuuca...............    1       1     bc7
............................................................ugucauggagucCcucucuuca...............    3       1     bc7
............................................................ugucauCgagucgcucucuuca...............    1       1     bc7
............................................................ugucauggagucgcucucuucaA..............    24      1     bc7
............................................................ugucauggagucgcucucuucaU..............    195     1     bc7
............................................................ugucauggagucgcucucuucag..............    1       0     bc7
............................................................ugucauggagGcgcucucuucag..............    2       1     bc7
............................................................ugucauggagucgcucucuucaC..............    3       1     bc7
............................................................ugucauggagucgcucucuucaUa.............    1       1     bc7
```

```
              cel-miR-46*
                                                            cel-miR-46
cugaggugaagcugaagagagccgucuauugacaguucaagaccacgagucguugugugcugucauggagucgcucucuucagaugauccggucaau

............................................................ugucauggagucgcucucuucagU.............   1    1    bc7
............................................................ugucauggagucgcucucuucaUau............   1    1    bc7
.............................................................gucauggagGcgcucucuuc................   1    1    bc7
.............................................................gucauggagucgUucucuuca...............   1    1    bc7
.............................................................gucauggagGcgcucucuuca...............   16   1    bc7
.............................................................gucauggagucgcucucuucU...............   3    1    bc7
.............................................................gucauggagucgcucucuuca...............   27   0    bc7
..............................................................ucauggagucgcucucuuca...............   1    0    bc7
..............................................................ucauggagGcgcucucuuca...............   1    1    bc7
..............................................................ucauggagAcgcucucuuca...............   1    1    bc7
...............................................................cauggagGcgcucucuuca...............   1    1    bc7
...............................................................cauggagucgcucucuuca...............   1    0    bc7
................................................................auggagucgcucucuuca...............   19   0    bc7
................................................................auggagGcgcucucuuca...............   15   1    bc7
................................................................auggagucgcucucuucaU..............   1    1    bc7
```
