# Supplementary material for: Reliable reference miRNAs for quantitative gene expression analysis of stress responses in Caenorhabditis elegans
Source: BMC Genomics. 2014 Mar 21;15:222. doi: 10.1186/1471-2164-15-222 (PMC3997968; doi:10.1186/1471-2164-15-222)
Supplement: Additional file 6: Figure S3 — Graphical output files from mirDeep2 showing the reads, counts per read and mapping on the hairpin for mir-2, mir-46 and mir-47. [file 1471-2164-15-222-S6.pdf]

miRBase precursor : cel-mir-47
Total read count : 173005
cel-miR-47 read count : 144266
cel-miR-47* read count : 28716
remaining reads : 23

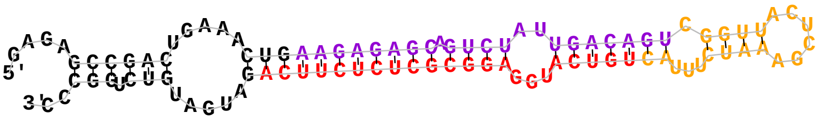

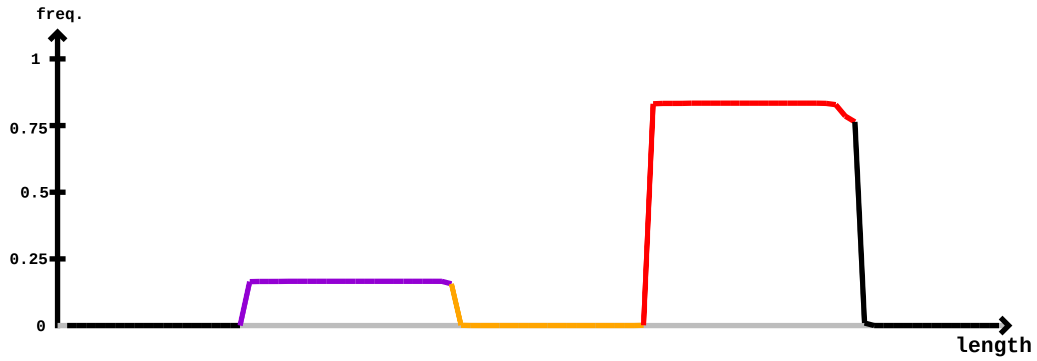

cel-miR-47*

cel-miR-47

5'- gagagccgacugaaacugaagagagcagucuauugacagucgguuacucgaaaucuuuacugucauggaggcgcucucuucagaugaugucuggccc -3' exp

....((((((.....(((((((((((.((((..(((((((.((((......))))...)))))))...))))))))))))))).....))).)))..	reads	mm	sample
..................aagagagcagucuauugacaA..	1	1	bc4
..................aagagagcUgucuauugacag..	1	1	bc4
..................aagagagcCgucuauugacag..	55	1	bc4
..................aagagagcagucuauugacag..	29	0	bc4
..................aagagagcagucuauugUcagu...	1	1	bc4
..................aagagagcCgucuauugacagu...	941	1	bc4
..................aagagagcagucuauugaAagu...	1	1	bc4
..................aagagagcagucuauugGcagu...	1	1	bc4
..................aagagagcagucuauugaUagu...	1	1	bc4
..................aagagaUcagucuauugacagu...	1	1	bc4
..................aagagagUagucuauugacagu...	1	1	bc4
..................aagagagcagucuauugacagG...	1	1	bc4
..................aagagagcagucuauugacagu...	542	0	bc4
..................aagagagcagucuGuugacagu...	1	1	bc4
..................aagagagcagucuauugCcagu...	1	1	bc4
..................aagagagcagucuauugacaguU..	9	1	bc4
..................aagagagcagucuauugacaguA..	1	1	bc4
..................aagagagcagucuauugacagucgguuacucgaaaucuuuac.....................................	1	0	bc4
...................agagagcCgucuauugacagu...	11	1	bc4
......................gagcagucuauugacagu...	1	0	bc4
......................gagcCgucuauugacagu...	1	1	bc4
....cugucauggagUcgcucucuu.................	1	1	bc4
....cugucauggagUcgcucucuuca...............	10	1	bc4
....cugucauggaggcgcucucuuca...............	3	0	bc4
....ugucauggagUcgcucuc...................	6	1	bc4
....ugucauggaggcgcucuc...................	3	0	bc4
....ugucauggaggcgcucucu..................	26	0	bc4
....ugucauggagUcgcucucu..................	7	1	bc4
....ugucauggaggcgcucucuG.................	1	1	bc4
....ugucauggaggcgAucucuu.................	1	1	bc4
....ugucauggaggcUcucucuu.................	1	1	bc4
....ugucauggagUcgcucucuu.................	130	1	bc4
....ugucauggaUgcgcucucuu.................	1	1	bc4
....ugucauggagCcgcucucuu.................	1	1	bc4

```
                  cel-miR-47*
                                          cel-miR-47
gagagccgacugaaacugaagagagcagucuauugacagucgguuacucgaaaucuuuacugucauggaggcgcucucuucagaugaugucuggccc

............................................................ugucauggaggcgcucucuu.................   234    0   bc4
............................................................ugucauggagAcgcucucuu.................   1      1   bc4
............................................................ugucauggaggcgAucucuuc................   1      1   bc4
............................................................ugucauggaggcgcucucuuU................   3      1   bc4
............................................................ugucauggaggGgcucucuuc................   1      1   bc4
............................................................ugucauggaggcgcucucuAc................   1      1   bc4
............................................................ugucauggaggcgcucucuuc................   93     0   bc4
............................................................ugucauggagUcgcucucuuc................   78     1   bc4
............................................................ugucauggagCcgcucucuuc................   1      1   bc4
............................................................ugucauggaggcgcucucuuca...............   1930   0   bc4
............................................................ugucauggGggcgcucucuuca...............   4      1   bc4
............................................................ugucauggaggcgcucuUuuca...............   1      1   bc4
............................................................ugucauggaggcgcucucuGca...............   2      1   bc4
............................................................ugucauggaggcgcucucuucC...............   8      1   bc4
............................................................ugucauggaggcgcucucuCca...............   2      1   bc4
............................................................ugucauggaggcAcucucuuca...............   1      1   bc4
............................................................ugucauggaggcgcucucuuUa...............   1      1   bc4
............................................................ugucauggCggcgcucucuuca...............   2      1   bc4
............................................................ugucauggaggcgAucucuuca...............   1      1   bc4
............................................................ugucauggaggcgcuUucuuca...............   3      1   bc4
............................................................ugucauggaggcgUucucuuca...............   1      1   bc4
............................................................ugucauggaUgcgcucucuuca...............   1      1   bc4
............................................................ugucauggaggGgcucucuuca...............   2      1   bc4
............................................................ugucauggaggcUcucucuuca...............   3      1   bc4
............................................................ugucauggUggcgcucucuuca...............   1      1   bc4
............................................................ugucauggagAcgcucucuuca...............   4      1   bc4
............................................................ugucauggagUcgcucucuuca...............   5628   1   bc4
............................................................ugucauggagCcgcucucuuca...............   8      1   bc4
............................................................ugucauggaggcgcucucuucG...............   5      1   bc4
............................................................ugucauggaggcCcucucuuca...............   2      1   bc4
............................................................ugucauggaggcgcucucuucU...............   289    1   bc4
............................................................ugucGuggaggcgcucucuuca...............   1      1   bc4
............................................................Cgucauggaggcgcucucuuca...............   1      1   bc4
............................................................ugucaCggaggcgcucucuuca...............   1      1   bc4
............................................................ugGcauggaggcgcucucuuca...............   1      1   bc4
............................................................ugucauggaggcgcucucuAca...............   2      1   bc4
............................................................ugucauCgaggcgcucucuuca...............   1      1   bc4
............................................................ugucauggaggcgcucucuucaA..............   4      1   bc4
............................................................ugucauggagUcgcucucuucag..............   3      1   bc4
............................................................ugucauggaggcgcucucuucaU..............   77     1   bc4
.............................................................gucauggaggcgcucucuuca...............   3      0   bc4
.............................................................gucauggagUcgcucucuuca...............   8      1   bc4
..............................................................ucauggagUcgcucucuuca...............   1      1   bc4
................................................................auggaggcgcucucuuca...............   1      0   bc4
................................................................auggagUcgcucucuuca...............   3      1   bc4

..................aagagagcCgucuauugac............................................................   1      1   bc1
..................aagagagcagucuauugaca...........................................................   1      0   bc1
..................aagagagcCgucuauugacag..........................................................   73     1   bc1
..................aagagagGagucuauugacag..........................................................   1      1   bc1
..................aagagagcagucuauugacag..........................................................   42     0   bc1
..................aagagagcagucuauugacagu.........................................................   786    0   bc1
..................aagagagcagucuauugacagC.........................................................   2      1   bc1
..................aagagagcagucuauugCcagu.........................................................   2      1   bc1
..................aagagGgcagucuauugacagu.........................................................   2      1   bc1
..................aagagagcCgucuauugacagu.........................................................   1238   1   bc1
..................aagagagcagucuauugaAagu.........................................................   1      1   bc1
..................aagGgagcagucuauugacagu.........................................................   1      1   bc1
..................aagagaUcagucuauugacagu.........................................................   1      1   bc1
..................aagagagcagucuauugacagG.........................................................   3      1   bc1
..................aagagagcagucuauugacaUu.........................................................   1      1   bc1
..................aagagagcagucuauugacGgu.........................................................   1      1   bc1
..................aagagCgcagucuauugacagu.........................................................   1      1   bc1
..................aagagaCcagucuauugacagu.........................................................   1      1   bc1
..................aagagagcagucuauugacaguc........................................................   2      0   bc1
..................aagagagcagucuauugacaguU........................................................   16     1   bc1
...................agagagcCgucuauugacagu.........................................................   15     1   bc1
...................agagagcagucuauugacagu.........................................................   1      0   bc1
....................gagagcCgucuauugacagu.........................................................   1      1   bc1
```

```
                  cel-miR-47*
                                                            cel-miR-47
gagagccgacugaaacugaagagagcagucuauugacagucgguuacucgaaaucuuuacugucauggaggcgcucucuucagaugaugucuggccc

......................gagcagucuauugacagu.........................................................    3      0    bc1
......................gagcCgucuauugacagu.........................................................    1      1    bc1
........................................cgguuacucgaaaucuuuac.....................................    3      0    bc1
...........................................................cugucauggaggcgcucucuuca...............    2      0    bc1
...........................................................cugucauggagUcgcucucuuca...............    5      1    bc1
............................................................ugucauggaggcgcucuc...................    4      0    bc1
............................................................ugucauggagUcgcucuc...................    5      1    bc1
............................................................ugucauggagUcgcucucu..................    6      1    bc1
............................................................ugucauggaggcgcucucu..................    24     0    bc1
............................................................ugucauggUggcgcucucuu.................    2      1    bc1
............................................................ugGcauggaggcgcucucuu.................    1      1    bc1
............................................................ugucauggaggcgcucucuu.................    238    0    bc1
............................................................ugucauggagUcgcucucuu.................    143    1    bc1
............................................................ugucauggaggcgcucucuuc................    155    0    bc1
............................................................ugucauggGggcgcucucuuc................    1      1    bc1
............................................................ugucauggagUcgcucucuuc................    67     1    bc1
............................................................ugucauggaggcgcucucuuU................    4      1    bc1
............................................................ugucauggaggcgcucucCuca...............    1      1    bc1
............................................................ugucauggaggcUcucucuuca...............    1      1    bc1
............................................................ugucauggaggcgcuAucuuca...............    2      1    bc1
............................................................ugucauggaggcgcucucuuGa...............    1      1    bc1
............................................................ugucauggaggcgcucucuCca...............    3      1    bc1
............................................................ugucaugUaggcgcucucuuca...............    1      1    bc1
............................................................ugucauUgaggcgcucucuuca...............    1      1    bc1
............................................................ugucauggaggAgcucucuuca...............    3      1    bc1
............................................................ugucauggagAcgcucucuuca...............    4      1    bc1
............................................................ugucauggaggcgcucucuucC...............    10     1    bc1
............................................................uguUauggaggcgcucucuuca...............    1      1    bc1
............................................................uAucauggaggcgcucucuuca...............    1      1    bc1
............................................................ugucauggaggcgcuGucuuca...............    1      1    bc1
............................................................ugucGuggaggcgcucucuuca...............    1      1    bc1
............................................................ugucaAggaggcgcucucuuca...............    1      1    bc1
............................................................ugucauggaggcgcuUucuuca...............    8      1    bc1
............................................................ugucauggaggcCcucucuuca...............    1      1    bc1
............................................................ugucauAgaggcgcucucuuca...............    1      1    bc1
............................................................ugucauggGggcgcucucuuca...............    6      1    bc1
............................................................ugucauggaggcgcucucuucU...............    355    1    bc1
............................................................ugucauggUggcgcucucuuca...............    5      1    bc1
............................................................ugucauggagUcgcucucuuca...............    7108   1    bc1
............................................................ugucauggaggcgcucucuAca...............    3      1    bc1
............................................................uCucauggaggcgcucucuuca...............    1      1    bc1
............................................................ugucauggaggcgcucucuucG...............    7      1    bc1
............................................................ugucauggaggGgcucucuuca...............    11     1    bc1
............................................................ugucauggaggcgcucuUuuca...............    4      1    bc1
............................................................ugucauggCggcgcucucuuca...............    2      1    bc1
............................................................ugucauggaggcgcucCcuuca...............    1      1    bc1
............................................................ugucauggaggcgcucucuuca...............    2911   0    bc1
............................................................ugucauggaggcgcucucuuAa...............    1      1    bc1
............................................................ugucauggaggUgcucucuuca...............    2      1    bc1
............................................................ugucauggaggcAcucucuuca...............    2      1    bc1
............................................................ugucauggaCgcgcucucuuca...............    2      1    bc1
............................................................ugucauggagCcgcucucuuca...............    13     1    bc1
............................................................ugucauggaggcgcucucuucaA..............    4      1    bc1
............................................................ugucauggaggcgcucucuucaU..............    99     1    bc1
............................................................ugucauggaggcgcucucuucaC..............    1      1    bc1
.............................................................gucauggagUcgcucucuuc................    1      1    bc1
.............................................................gucauggagUcgcucucuuca...............    15     1    bc1
.............................................................gucauggaggcgcucucuucU...............    1      1    bc1
.............................................................gucauggaggcgcucucuuca...............    3      0    bc1
.............................................................gucauggaggcgcucucuucaU..............    1      1    bc1
..............................................................ucauggagUcgcucucuuca...............    1      1    bc1
..............................................................ucauggaggcgcucucuucaga.............    1      0    bc1
................................................................auggaggcgcucucuuca...............    3      0    bc1
................................................................auggaggcgcucucuucU...............    1      1    bc1
................................................................auggagUcgcucucuuca...............    2      1    bc1

..................aagagagcCgucuauugac............................................................    2      1    bc3
..................aagagagcagucuauugaca...........................................................    1      0    bc3
..................aagagagcCgucuauugaca...........................................................    1      1    bc3
```

```
                  cel-miR-47*
                                                                                cel-miR-47
gagagccgacugaaacugaagagagcagucuauugacagucgguuacucgaaaucuuuacugucauggaggcgcucucuucagaugaugucuggccc

..................aagagagcagucuauugacag..........................................................   67     0   bc3
..................aagagagcagucuauuUacag..........................................................   1      1   bc3
..................aagagagcGgucuauugacag..........................................................   1      1   bc3
..................aagagagcCgucuauugacag..........................................................   203    1   bc3
..................Uagagagcagucuauugacagu.........................................................   1      1   bc3
..................aagagagcagucuauugaUagu.........................................................   1      1   bc3
..................aagagagcaUucuauugacagu.........................................................   2      1   bc3
..................aagagagcagucuGuugacagu.........................................................   1      1   bc3
..................aagagagcCgucuauugacagu.........................................................   2684   1   bc3
..................Gagagagcagucuauugacagu.........................................................   1      1   bc3
..................aagagagcagucuauugacagG.........................................................   4      1   bc3
..................aagagagcaguUuauugacagu.........................................................   1      1   bc3
..................aagagagcUgucuauugacagu.........................................................   1      1   bc3
..................aagagagcagucuauugacagu.........................................................   1096   0   bc3
..................aagagagcagucuauugacaguU........................................................   17     1   bc3
..................aagagagcagucuauugacaguc........................................................   1      0   bc3
..................aagagagcagucuauugacagucgguuacucgaaaucuuuac.....................................   1      0   bc3
...................agagagcCgucuauugacag..........................................................   3      1   bc3
...................agagagcCgucuauugacagu.........................................................   22     1   bc3
...................agagagcagucuauugacagu.........................................................   1      0   bc3
....................gagagcCgucuauugacagu.........................................................   1      1   bc3
.....................agagcagucuauugacagu.........................................................   1      0   bc3
......................gagcagucuauugacagu.........................................................   2      0   bc3
......................gagcCgucuauugacagu.........................................................   4      1   bc3
........................................cgguuacucgaaaucuuuac.....................................   5      0   bc3
...........................................................cugucauggagUcgcucucuuc................   1      1   bc3
...........................................................cugucauggaggcgcucucuuc................   1      0   bc3
...........................................................cugucauggaggcgcucucuuca...............   2      0   bc3
...........................................................cugucauggagUcgcucucuuca...............   10     1   bc3
............................................................ugucauggaggcgcucuc...................   10     0   bc3
............................................................ugucauggagUcgcucuc...................   15     1   bc3
............................................................ugucauggaggcgcucucG..................   1      1   bc3
............................................................ugucauggGggcgcucucu..................   1      1   bc3
............................................................ugucauggaggcgcucucC..................   1      1   bc3
............................................................ugucauggaggcgcucucu..................   74     0   bc3
............................................................ugucauggagUcgcucucu..................   27     1   bc3
............................................................ugucaugUaggcgcucucuu.................   1      1   bc3
............................................................Cgucauggaggcgcucucuu.................   1      1   bc3
............................................................ugucauggaggcgcucucCu.................   2      1   bc3
............................................................ugucauggagAcgcucucuu.................   1      1   bc3
............................................................ugucauggagCcgcucucuu.................   3      1   bc3
............................................................ugGcauggaggcgcucucuu.................   1      1   bc3
............................................................ugucauggaggGgcucucuu.................   1      1   bc3
............................................................ugucauggGggcgcucucuu.................   2      1   bc3
............................................................uguUauggaggcgcucucuu.................   1      1   bc3
............................................................ugucauggaggcgcucucuu.................   569    0   bc3
............................................................ugucauggUggcgcucucuu.................   2      1   bc3
............................................................ugucauggagUcgcucucuu.................   385    1   bc3
............................................................ugucGuggaggcgcucucuuc................   1      1   bc3
............................................................ugucauggaggcgcAcucuuc................   1      1   bc3
............................................................ugucauggaggcgUucucuuc................   1      1   bc3
............................................................ugucauggCggcgcucucuuc................   1      1   bc3
............................................................ugucauggagUcgcucucuuc................   172    1   bc3
............................................................ugucauggaggcgcucucuuc................   303    0   bc3
............................................................ugucauggaggcgcucucuuA................   1      1   bc3
............................................................ugAcauggaggcgcucucuuc................   1      1   bc3
............................................................ugucauggaggGgcucucuuc................   2      1   bc3
............................................................ugucauggaggcgcucucuuU................   5      1   bc3
............................................................Agucauggaggcgcucucuuc................   1      1   bc3
............................................................ugucaugAaggcgcucucuuca...............   1      1   bc3
............................................................ugucauggaggcCcucucuuca...............   3      1   bc3
............................................................Agucauggaggcgcucucuuca...............   2      1   bc3
............................................................ugucGuggaggcgcucucuuca...............   1      1   bc3
............................................................ugucauggaggcgcucucuucU...............   678    1   bc3
............................................................ugucauggaggcgUucucuuca...............   1      1   bc3
............................................................ugucauggaggcgcucucuucC...............   10     1   bc3
............................................................ugucauggaCgcgcucucuuca...............   2      1   bc3
............................................................ugucaugUaggcgcucucuuca...............   1      1   bc3
............................................................ugucauggaggcgcucucuCca...............   7      1   bc3
```

cel-miR-47*

cel-miR-47

```
gagagccgacugaaacugaagagagcagucuauugacagucgguuacucgaaaucuuuacugucauggaggcgcucucuucagaugaugucuggccc

............................................................ugucUuggaggcgcucucuuca...............	3	1	bc3
............................................................ugucauggaggAgcucucuuca...............	6	1	bc3
............................................................ugucauggaggcgcuUucuuca...............	6	1	bc3
............................................................ugucauggaggcgcucucuuUa...............	2	1	bc3
............................................................ugucauggaggcUcucucuuca...............	2	1	bc3
............................................................ugucauggaggcgcucucuAca...............	3	1	bc3
............................................................ugucauggaggcgcucucuuca...............	4629	0	bc3
............................................................ugucauggagCcgcucucuuca...............	30	1	bc3
............................................................ugucauggaggcgcucucuuAa...............	8	1	bc3
............................................................ugucauggaggcgcucuUuuca...............	3	1	bc3
............................................................ugucauggaggcgcucucuGca...............	4	1	bc3
............................................................ugucauggaUgcgcucucuuca...............	1	1	bc3
............................................................ugucauggCggcgcucucuuca...............	1	1	bc3
............................................................ugucauggGggcgcucucuuca...............	6	1	bc3
............................................................ugucauggagAcgcucucuuca...............	10	1	bc3
............................................................ugucauggaggcgAucucuuca...............	1	1	bc3
............................................................ugucauUgaggcgcucucuuca...............	1	1	bc3
............................................................ugucauggaggcgcucucuucG...............	5	1	bc3
............................................................uguUauggaggcgcucucuuca...............	2	1	bc3
............................................................ugucauggaggUgcucucuuca...............	1	1	bc3
............................................................ugucauggaggGgcucucuuca...............	9	1	bc3
............................................................ugucauggaggcgcuAucuuca...............	1	1	bc3
............................................................ugucauggaggcgcucCcuuca...............	3	1	bc3
............................................................ugGcauggaggcgcucucuuca...............	2	1	bc3
............................................................ugucaCggaggcgcucucuuca...............	2	1	bc3
............................................................ugucauggaggcAcucucuuca...............	4	1	bc3
............................................................ugucauAgaggcgcucucuuca...............	2	1	bc3
............................................................uAucauggaggcgcucucuuca...............	3	1	bc3
............................................................ugucauggUggcgcucucuuca...............	3	1	bc3
............................................................ugucauggagUcgcucucuuca...............	14521	1	bc3
............................................................ugucauggagUcgcucucuucag..............	4	1	bc3
............................................................ugucauggaggcgcucucuucaU..............	241	1	bc3
............................................................ugucauggaggcgcucucuucaA..............	13	1	bc3
............................................................ugucauggagUcgcucucuucaga.............	1	1	bc3
.............................................................gucauggaggcgcucucuu.................	1	0	bc3
.............................................................gucauggaggcgcucucuuc................	1	0	bc3
.............................................................gucauggaggcgcucucuuca...............	8	0	bc3
.............................................................gucauggagUcgcucucuuca...............	18	1	bc3
.............................................................gucauggaggcgcucucuucU...............	1	1	bc3
..............................................................ucauggagUcgcucucuuca...............	2	1	bc3
...............................................................cauggaggcgcucucuuca...............	1	0	bc3
................................................................auggaggcgcucucuuca...............	3	0	bc3
................................................................auggagUcgcucucuuca...............	11	1	bc3
................................................................auggaggcgcucucuucU...............	1	1	bc3

.................Caagagagcagucuauugacagu.........................................................	1	1	bc6
..................aagagCgcagucuauugacag..........................................................	1	1	bc6
..................aagagagcagucuauugacag..........................................................	102	0	bc6
..................aagagagGagucuauugacag..........................................................	1	1	bc6
..................aagagagcagucuauugaAag..........................................................	1	1	bc6
..................Gagagagcagucuauugacag..........................................................	1	1	bc6
..................aagagagcCgucuauugacag..........................................................	166	1	bc6
..................aagagagcagucuauugacGgu.........................................................	2	1	bc6
..................aagagCgcagucuauugacagu.........................................................	1	1	bc6
..................aagagGgcagucuauugacagu.........................................................	3	1	bc6
..................aagagagcagucuauugacagA.........................................................	1	1	bc6
..................aagagagcaguGuauugacagu.........................................................	1	1	bc6
..................aagagaCcagucuauugacagu.........................................................	2	1	bc6
..................aagagagcagucuauugacaCu.........................................................	1	1	bc6
..................aagagagcagucuCuugacagu.........................................................	1	1	bc6
..................aagagagcagucuauugGcagu.........................................................	1	1	bc6
..................aGgagagcagucuauugacagu.........................................................	1	1	bc6
..................aagagUgcagucuauugacagu.........................................................	3	1	bc6
..................aagagagcGgucuauugacagu.........................................................	1	1	bc6
..................aagagagcagucuauugacagu.........................................................	2417	0	bc6
..................aagagagcagucuauugacaAu.........................................................	3	1	bc6
..................aUgagagcagucuauugacagu.........................................................	1	1	bc6
..................aagagagcagucCauugacagu.........................................................	1	1	bc6
..................aagagagcagucuauUacagu..........................................................	1	1	bc6
```

```
                  cel-miR-47*
                                                            cel-miR-47
gagagccgacugaaacugaagagagcagucuauugacagucgguuacucgaaaucuuuacugucauggaggcgcucucuucagaugaugucuggccc

..................aagagagcagucuauugaAagu.........................................................   2      1   bc6
..................aagagagcagCcuauugacagu.........................................................   3      1   bc6
..................aagagagcagucuUuugacagu.........................................................   1      1   bc6
..................aagagagAagucuauugacagu.........................................................   1      1   bc6
..................aagagagcagucuauugacCgu.........................................................   1      1   bc6
..................Gagagagcagucuauugacagu.........................................................   1      1   bc6
..................aagGgagcagucuauugacagu.........................................................   1      1   bc6
..................aagagagcCgucuauugacagu.........................................................   2869   1   bc6
..................aagagagcagucuauugacagG.........................................................   10     1   bc6
..................aagagagcagucuauugacaguc........................................................   1      0   bc6
..................aagagagcagucuauugacaguG........................................................   1      1   bc6
..................aagagagcagucuauugacaguA........................................................   1      1   bc6
..................aagagagcagucuauugacaguU........................................................   39     1   bc6
..................aagagagcagucuauugacagucU.......................................................   1      1   bc6
..................aagagagcagucuauugacagucgguuacucgaaaucuuuac.....................................   1      0   bc6
...................agagagcagucuauugacag..........................................................   1      0   bc6
...................agagagcCgucuauugacagu.........................................................   15     1   bc6
...................agagagcagucuauugacagu.........................................................   5      0   bc6
....................gagagcCgucuauugacag..........................................................   1      1   bc6
.....................agagcCgucuauugacagu.........................................................   1      1   bc6
......................gagcagucuauugacagu.........................................................   11     0   bc6
......................gagcCgucuauugacagu.........................................................   6      1   bc6
........................................cgguuacucgaaaucuuuac.....................................   4      0   bc6
...........................................................cugucauggagUcgcucucuu.................   2      1   bc6
...........................................................cugucauggaggcgcucucuucU...............   1      1   bc6
...........................................................cugucauggaggcgcucucuuca...............   4      0   bc6
...........................................................cugucauggagUcgcucucuuca...............   24     1   bc6
............................................................ugucauggaggcgcucuc...................   8      0   bc6
............................................................ugucauggagUcgcucuc...................   11     1   bc6
............................................................ugucauggaggcgcucucu..................   94     0   bc6
............................................................ugucauggaggcgcucucG..................   1      1   bc6
............................................................ugucauggaggGgcucucu..................   1      1   bc6
............................................................ugucauggagUcgcucucu..................   27     1   bc6
............................................................ugucauggaggcgcucGcu..................   1      1   bc6
............................................................ugucauggGggcgcucucu..................   1      1   bc6
............................................................ugGcauggaggcgcucucu..................   1      1   bc6
............................................................ugucauggaggcgcucuGuu.................   1      1   bc6
............................................................ugucauggagCcgcucucuu.................   3      1   bc6
............................................................ugucauggaggcgcucucuu.................   710    0   bc6
............................................................ugucauggaggcgcucuUuu.................   1      1   bc6
............................................................ugucauggaggcgcucucuA.................   1      1   bc6
............................................................ugucUuggaggcgcucucuu.................   1      1   bc6
............................................................ugucauggagUcgcucucuu.................   417    1   bc6
............................................................ugucaugAaggcgcucucuu.................   1      1   bc6
............................................................ugucauggagAcgcucucuu.................   2      1   bc6
............................................................ugucauggaggAgcucucuu.................   2      1   bc6
............................................................ugucauggaggcgcucCcuu.................   1      1   bc6
............................................................ugucauggCggcgcucucuu.................   1      1   bc6
............................................................ugucauggaUgcgcucucuu.................   1      1   bc6
............................................................ugucauggaggcgcucucuG.................   2      1   bc6
............................................................ugucauggaggcgcCcucuu.................   1      1   bc6
............................................................ugucauggaggcgcucucuuU................   6      1   bc6
............................................................ugucauggaggcgcucucuuc................   383    0   bc6
............................................................ugucauggaggcgcuGucuuc................   1      1   bc6
............................................................ugucauggaggcgcucucuGc................   1      1   bc6
............................................................ugucauggaggcgcucucCuc................   1      1   bc6
............................................................ugucauggagUcgcucucuuc................   188    1   bc6
............................................................ugCcauggaggcgcucucuuc................   1      1   bc6
............................................................Ggucauggaggcgcucucuuc................   1      1   bc6
............................................................ugucauggaggcgcucucuuUa...............   2      1   bc6
............................................................ugucauUgaggcgcucucuuca...............   3      1   bc6
............................................................ugucaCggaggcgcucucuuca...............   1      1   bc6
............................................................ugucauggaggcAcucucuuca...............   1      1   bc6
............................................................ugucauggaggcCcucucuuca...............   4      1   bc6
............................................................ugucauggaggcgcucCcuuca...............   1      1   bc6
............................................................ugucauggaggcgcucucuucU...............   1016   1   bc6
............................................................ugucauggaggcgcuUucuuca...............   2      1   bc6
............................................................ugAcauggaggcgcucucuuca...............   3      1   bc6
............................................................ugucauggagUcgcucucuuca...............   15360  1   bc6
```

```
                  cel-miR-47*
                                                            cel-miR-47
gagagccgacugaaacugaagagagcagucuauugacagucgguuacucgaaaucuuuacugucauggaggcgcucucuucagaugaugucuggccc

............................................................ugucauggaggGgcucucuuca...............  12      1     bc6
............................................................ugucCuggaggcgcucucuuca...............  2       1     bc6
............................................................ugucauggaggAgcucucuuca...............  7       1     bc6
............................................................ugucauggaAgcgcucucuuca...............  1       1     bc6
............................................................ugucauggaggcgcCcucuuca...............  5       1     bc6
............................................................ugucauggaggcgcGcucuuca...............  2       1     bc6
............................................................ugucauggCggcgcucucuuca...............  3       1     bc6
............................................................ugucauggaggcgcucucuucC...............  18      1     bc6
............................................................ugCcauggaggcgcucucuuca...............  4       1     bc6
............................................................ugucauggaggcgcucuUuuca...............  4       1     bc6
............................................................ugucauggGggcgcucucuuca...............  8       1     bc6
............................................................uguUauggaggcgcucucuuca...............  1       1     bc6
............................................................ugucauggaggcgcucucuGca...............  3       1     bc6
............................................................ugucauggaggcgcucucuuAa...............  2       1     bc6
............................................................ugucauggaggcgcucucuAca...............  4       1     bc6
............................................................ugucaugCaggcgcucucuuca...............  2       1     bc6
............................................................Cgucauggaggcgcucucuuca...............  5       1     bc6
............................................................ugucauggaggcgcuAucuuca...............  1       1     bc6
............................................................ugucauggaggcgcucucuucG...............  24      1     bc6
............................................................uguAauggaggcgcucucuuca...............  1       1     bc6
............................................................ugucauggaggUgcucucuuca...............  6       1     bc6
............................................................ugucauCgaggcgcucucuuca...............  1       1     bc6
............................................................ugucauggaggcgcucuGuuca...............  3       1     bc6
............................................................ugucauggaggcgcucucuCca...............  20      1     bc6
............................................................ugucauggagAcgcucucuuca...............  14      1     bc6
............................................................ugucauggaggcgcAcucuuca...............  1       1     bc6
............................................................ugucauggUggcgcucucuuca...............  4       1     bc6
............................................................ugGcauggaggcgcucucuuca...............  1       1     bc6
............................................................ugucauggaggcgcucuCuuca...............  2       1     bc6
............................................................ugucauggagCcgcucucuuca...............  26      1     bc6
............................................................ugucauggaggcgcucucuuca...............  5359    0     bc6
............................................................ugucauggaggcgcucGcuuca...............  2       1     bc6
............................................................ugucGuggaggcgcucucuuca...............  3       1     bc6
............................................................Ggucauggaggcgcucucuuca...............  1       1     bc6
............................................................ugucaugUaggcgcucucuuca...............  2       1     bc6
............................................................ugucUuggaggcgcucucuuca...............  1       1     bc6
............................................................ugucauggagUcgcucucuucag..............  4       1     bc6
............................................................ugucauggUggcgcucucuucag..............  1       1     bc6
............................................................ugucauggaggcgcucucuucaU..............  249     1     bc6
............................................................ugucauggaggcgcucucuucaA..............  10      1     bc6
............................................................ugucauggaggcgcucucuucaC..............  1       1     bc6
............................................................ugucauggaggcgcucucuucaAa.............  1       1     bc6
.............................................................gucauggaggcgcucucuu.................  3       0     bc6
.............................................................gucauggagUcgcucucuuca...............  31      1     bc6
.............................................................gucauggaggcgcucucuuca...............  10      0     bc6
.............................................................gucauggaggcgcucucuucU...............  7       1     bc6
..............................................................ucauggaggcgcucucuuca...............  1       0     bc6
..............................................................ucauggagUcgcucucuuca...............  1       1     bc6
................................................................auggagUcgcucucuuca...............  19      1     bc6
................................................................auggaggcgcucucuuca...............  14      0     bc6

..................aagagagcagucuauugac............................................................  1       0     bc5
..................aagagagcCgucuauugac............................................................  1       1     bc5
..................aagagagcagucuauugaca...........................................................  2       0     bc5
..................aagagagcagucuauugaUag..........................................................  1       1     bc5
..................aagagagcCgucuauugacag..........................................................  265     1     bc5
..................aagagagcagucuauugacag..........................................................  136     0     bc5
..................aagagagcagucuauugacCgu.........................................................  1       1     bc5
..................aGgagagcagucuauugacagu.........................................................  2       1     bc5
..................aagagGgcagucuauugacagu.........................................................  2       1     bc5
..................aagagagcagucuauugacagG.........................................................  4       1     bc5
..................aagagagcagucuauugUcagu.........................................................  1       1     bc5
..................aagagagcagucuauCgacagu.........................................................  1       1     bc5
..................aagagagcCgucuauugacagu.........................................................  3534    1     bc5
..................aagagagcagucuauugacGgu.........................................................  5       1     bc5
..................aagagagcagucuauugaUagu.........................................................  2       1     bc5
..................aagagCgcagucuauugacagu.........................................................  1       1     bc5
..................aagagagcagAcuauugacagu.........................................................  2       1     bc5
..................aagagaAcagucuauugacagu.........................................................  1       1     bc5
```

```
                  cel-miR-47*
                                                            cel-miR-47
gagagccgacugaaacugaagagagcagucuauugacagucgguuacucgaaaucuuuacugucauggaggcgcucucuucagaugaugucuggccc

..................aagagagcagucuauuCacagu.........................................................   1     1   bc5
..................aagagagcagucuauugacagC.........................................................   2     1   bc5
..................aagaAagcagucuauugacagu.........................................................   1     1   bc5
..................aaAagagcagucuauugacagu.........................................................   1     1   bc5
..................aagagagcagucuauugacagu.........................................................   2526  0   bc5
..................aagagagcagucuauugCcagu.........................................................   1     1   bc5
..................aagCgagcagucuauugacagu.........................................................   1     1   bc5
..................aagagagcagucuauugacaUu.........................................................   1     1   bc5
..................aagagagcagGcuauugacagu.........................................................   2     1   bc5
..................aagagaUcagucuauugacagu.........................................................   2     1   bc5
..................aagaUagcagucuauugacagu.........................................................   1     1   bc5
..................aagagagcUgucuauugacagu.........................................................   1     1   bc5
..................aagagagcagucuGuugacagu.........................................................   2     1   bc5
..................Gagagagcagucuauugacagu.........................................................   1     1   bc5
..................aagagagcagCcuauugacagu.........................................................   1     1   bc5
..................aagagagcagucuauugacaAu.........................................................   1     1   bc5
..................aagagagcaAucuauugacagu.........................................................   1     1   bc5
..................aagagagcagucuaCugacagu.........................................................   2     1   bc5
..................aagagagcagucuauugacaguU........................................................   36    1   bc5
..................aagagagcagucuauugacaguc........................................................   3     0   bc5
..................aagagagcagucuauugacaguA........................................................   1     1   bc5
..................aagagagcagucuauugacaguUg.......................................................   1     1   bc5
...................agagagcCgucuauugacag..........................................................   1     1   bc5
...................agagagcCgucuauugacagu.........................................................   14    1   bc5
...................agagagcagucuauugacagu.........................................................   3     0   bc5
...................agagagcagucuauugacaguU........................................................   1     1   bc5
.....................agagcagucuauugacagu.........................................................   2     0   bc5
......................gagcagucuauugacagu.........................................................   12    0   bc5
......................gagcCgucuauugacagu.........................................................   17    1   bc5
........................................cgguuacucgaaaucuuuac.....................................   6     0   bc5
...........................................................cugucauggagUcgcucucuu.................   2     1   bc5
...........................................................cugucauggagUcgcucucuuc................   1     1   bc5
...........................................................cugucauggaggcgcucucuuc................   1     0   bc5
...........................................................cugucauggaggcgcucucuucU...............   1     1   bc5
...........................................................cugucauggaggcgcucucuuca...............   5     0   bc5
...........................................................cugucauggagUcgcucucuuca...............   29    1   bc5
...........................................................cugucauggaggcgcucucuucaU..............   2     1   bc5
............................................................ugucauggagUcgcucuc...................   13    1   bc5
............................................................ugucauggaggcgcucuc...................   10    0   bc5
............................................................ugucauggaggcgcucuUu..................   1     1   bc5
............................................................ugucauggagUcgcucucu..................   41    1   bc5
............................................................ugucauggaggcgcucucu..................   157   0   bc5
............................................................ugucauggCggcgcucucu..................   1     1   bc5
............................................................ugucauggaggcgcucucG..................   3     1   bc5
............................................................ugucauggagCcgcucucuu.................   1     1   bc5
............................................................ugucauggaggcgcucucuA.................   1     1   bc5
............................................................uCucauggaggcgcucucuu.................   1     1   bc5
............................................................ugucauggagUcgcucucuu.................   662   1   bc5
............................................................ugucauggaggcgcucucuu.................   958   0   bc5
............................................................ugucauggUggcgcucucuu.................   2     1   bc5
............................................................ugucaGggaggcgcucucuu.................   1     1   bc5
............................................................ugucauggaggAgcucucuu.................   2     1   bc5
............................................................ugucauggaggcgcucucCu.................   1     1   bc5
............................................................ugucauggaggcgcucCcuu.................   1     1   bc5
............................................................ugCcauggaggcgcucucuu.................   1     1   bc5
............................................................ugucauggaggcgcuUucuu.................   2     1   bc5
............................................................ugucauggaggGgcucucuu.................   1     1   bc5
............................................................ugucauggaggcUcucucuuc................   2     1   bc5
............................................................ugucauAgaggcgcucucuuc................   1     1   bc5
............................................................ugucauggaggcgcucucuuU................   11    1   bc5
............................................................ugAcauggaggcgcucucuuc................   1     1   bc5
............................................................ugucauggaggcgcCcucuuc................   1     1   bc5
............................................................ugucauggagAcgcucucuuc................   1     1   bc5
............................................................ugucauggaggcgcucucuuG................   1     1   bc5
............................................................ugucauggaggcgcucucuuc................   552   0   bc5
............................................................ugGcauggaggcgcucucuuc................   1     1   bc5
............................................................ugucauggaggcAcucucuuc................   1     1   bc5
............................................................ugucauggCggcgcucucuuc................   1     1   bc5
............................................................ugucauggaggcgcucuAuuc................   1     1   bc5
```

```
                  cel-miR-47*
                                                            cel-miR-47
gagagccgacugaaacugaagagagcagucuauugacagucgguuacucgaaaucuuuacugucauggaggcgcucucuucagaugaugucuggccc

............................................................ugucauggaggcgcucucuuA................    1       1    bc5
............................................................ugucauggUggcgcucucuuc................    1       1    bc5
............................................................ugucauggagUcgcucucuuc................    290     1    bc5
............................................................ugucauggaggcgcucucuuAa...............    3       1    bc5
............................................................ugucauggaggcgcucAcuuca...............    1       1    bc5
............................................................ugucauggaggcgcucucuucC...............    22      1    bc5
............................................................ugucauggaggcgcucucuuca...............    7365    0    bc5
............................................................ugucauggagUcgcucucuuca...............    20732   1    bc5
............................................................ugucauggaggcAcucucuuca...............    1       1    bc5
............................................................ugucauggaggcgcucucuCca...............    17      1    bc5
............................................................ugucauggaggcgcucucuucU...............    1289    1    bc5
............................................................Agucauggaggcgcucucuuca...............    1       1    bc5
............................................................ugucaAggaggcgcucucuuca...............    3       1    bc5
............................................................ugucUuggaggcgcucucuuca...............    1       1    bc5
............................................................uguUauggaggcgcucucuuca...............    2       1    bc5
............................................................ugucauUgaggcgcucucuuca...............    6       1    bc5
............................................................ugucauggaggcgUucucuuca...............    1       1    bc5
............................................................ugucaugAaggcgcucucuuca...............    2       1    bc5
............................................................ugCcauggaggcgcucucuuca...............    1       1    bc5
............................................................ugucauggaggcgGucucuuca...............    1       1    bc5
............................................................ugucauggaggcgcucucuGca...............    3       1    bc5
............................................................uguAauggaggcgcucucuuca...............    1       1    bc5
............................................................ugucauggGggcgcucucuuca...............    6       1    bc5
............................................................ugucauggaggcgcucucCuca...............    2       1    bc5
............................................................ugucauggaggcgcucuAuuca...............    1       1    bc5
............................................................ugGcauggaggcgcucucuuca...............    1       1    bc5
............................................................ugucauggaggcgcucucuAca...............    3       1    bc5
............................................................Cgucauggaggcgcucucuuca...............    3       1    bc5
............................................................uUucauggaggcgcucucuuca...............    1       1    bc5
............................................................ugucauggagAcgcucucuuca...............    9       1    bc5
............................................................ugucauggaggAgcucucuuca...............    2       1    bc5
............................................................ugucauggaggGgcucucuuca...............    9       1    bc5
............................................................ugucaCggaggcgcucucuuca...............    1       1    bc5
............................................................ugucauggaggcgcGcucuuca...............    1       1    bc5
............................................................ugucauggaggcgcuUucuuca...............    2       1    bc5
............................................................ugucauggaggcgcucCcuuca...............    2       1    bc5
............................................................ugucGuggaggcgcucucuuca...............    2       1    bc5
............................................................ugucauggaggcgcuAucuuca...............    2       1    bc5
............................................................ugucauggCggcgcucucuuca...............    2       1    bc5
............................................................ugucauggaggcUcucucuuca...............    1       1    bc5
............................................................ugucauggUggcgcucucuuca...............    2       1    bc5
............................................................ugucauggaggUgcucucuuca...............    5       1    bc5
............................................................ugucauggaggcgcCcucuuca...............    1       1    bc5
............................................................ugucauAgaggcgcucucuuca...............    3       1    bc5
............................................................ugucauggaggcgcucucuuUa...............    5       1    bc5
............................................................ugucauggaggcgAucucuuca...............    4       1    bc5
............................................................ugucauggaggcgcucucuucG...............    32      1    bc5
............................................................ugucaugUaggcgcucucuuca...............    2       1    bc5
............................................................ugucauggaggcgcAcucuuca...............    1       1    bc5
............................................................ugAcauggaggcgcucucuuca...............    1       1    bc5
............................................................ugucauggagCcgcucucuuca...............    14      1    bc5
............................................................ugucauggaCgcgcucucuuca...............    1       1    bc5
............................................................ugucauggaggcgcucuUuuca...............    3       1    bc5
............................................................ugucauggagUcgcucucuucag..............    4       1    bc5
............................................................ugucauggaggcgcucucuucaU..............    408     1    bc5
............................................................ugucauggaggcgcucucuucaA..............    15      1    bc5
............................................................ugucauggaggcgcucucuucag..............    2       0    bc5
............................................................ugucauggagUcgcucucuucaga.............    1       1    bc5
............................................................ugucauggaggcgcucucuucaUa.............    2       1    bc5
.............................................................gucauggaggcgcucucuu.................    1       0    bc5
.............................................................gucauggagUcgcucucuuca...............    30      1    bc5
.............................................................gucauggaggcgcucucuuca...............    14      0    bc5
.............................................................gucauggaggcgcucucuucU...............    5       1    bc5
..............................................................ucauggagUcgcucucuuca...............    4       1    bc5
...............................................................cauggaggcgcucucuuca...............    2       0    bc5
................................................................auggagUcgcucucuuca...............    22      1    bc5
................................................................auggaggcgcucucuuca...............    12      0    bc5

..................aagagagcCgucuauugaca...........................................................    1       1    bc2
```

cel-miR-47*

cel-miR-47

```
gagagccgacugaaacugaagagagcagucuauugacagucgguuacucgaaaucuuuacugucauggaggcgcucucuucagaugaugucuggccc

..................aagagagcagucuauugacGg..........................................................  1     1  bc2
..................aagagagcagucuauugacag..........................................................  33    0  bc2
..................aagagagcCgucuauugacag..........................................................  92    1  bc2
..................aagagagcUgucuauugacag..........................................................  1     1  bc2
..................aagagaUcagucuauugacagu.........................................................  1     1  bc2
..................aagagagcagucuauugacUgu.........................................................  1     1  bc2
..................aagagagcagAcuauugacagu.........................................................  1     1  bc2
..................aagagagcagucuauugacagu.........................................................  786   0  bc2
..................aagagagcaUucuauugacagu.........................................................  1     1  bc2
..................aagagagcaguAuauugacagu.........................................................  1     1  bc2
..................aagagagcagucuaCugacagu.........................................................  1     1  bc2
..................aagagagcCgucuauugacagu.........................................................  1397  1  bc2
..................aagagagcagCcuauugacagu.........................................................  1     1  bc2
..................aagagagcagGcuauugacagu.........................................................  1     1  bc2
..................aagagagcagucuauugacaAu.........................................................  1     1  bc2
..................aagagagcagucCauugacagu.........................................................  1     1  bc2
..................Gagagagcagucuauugacagu.........................................................  1     1  bc2
..................aagagagGagucuauugacagu.........................................................  2     1  bc2
..................aagCgagcagucuauugacagu.........................................................  1     1  bc2
..................aagagagcagucuauugacaguU........................................................  13    1  bc2
...................agagagcCgucuauugacagu.........................................................  2     1  bc2
...................agagagcagucuauugacagucU.......................................................  1     1  bc2
.....................agagcCgucuauugacagu.........................................................  1     1  bc2
......................gagcagucuauugacagu.........................................................  4     0  bc2
......................gagcCgucuauugacagu.........................................................  6     1  bc2
...........................................................cugucauggaggcgcucucuu.................  2     0  bc2
...........................................................cugucauggagUcgcucucuuc................  1     1  bc2
...........................................................cugucauggaggcgcucucuuca...............  3     0  bc2
...........................................................cugucauggagUcgcucucuuca...............  16    1  bc2
............................................................ugucauggaggcgcucuc...................  9     0  bc2
............................................................ugucauggagUcgcucuc...................  11    1  bc2
............................................................ugucauggaggcgcucucu..................  44    0  bc2
............................................................ugucauggagUcgcucucu..................  17    1  bc2
............................................................ugucauggaggcgcucucuC.................  1     1  bc2
............................................................ugucauggUggcgcucucuu.................  1     1  bc2
............................................................ugucauggaggcgcucucuu.................  391   0  bc2
............................................................ugucauggaggcgcucucuA.................  1     1  bc2
............................................................ugucauggCggcgcucucuu.................  1     1  bc2
............................................................ugucaAggaggcgcucucuu.................  1     1  bc2
............................................................ugucauggGggcgcucucuu.................  1     1  bc2
............................................................ugucauggaggGgcucucuu.................  2     1  bc2
............................................................ugucauggagUcgcucucuu.................  302   1  bc2
............................................................ugucaugAaggcgcucucuu.................  1     1  bc2
............................................................ugucauggaggGgcucucuuc................  1     1  bc2
............................................................ugucauggaggcgcGcucuuc................  1     1  bc2
............................................................ugucauggaggcgcucucuuU................  4     1  bc2
............................................................ugucauggagCcgcucucuuc................  1     1  bc2
............................................................ugucauggGggcgcucucuuc................  1     1  bc2
............................................................ugucauggaggcgcCcucuuc................  1     1  bc2
............................................................ugucauggaggcgcucucuuc................  256   0  bc2
............................................................ugucauggagUcgcucucuuc................  151   1  bc2
............................................................ugucauggaggcgcucucuuGa...............  2     1  bc2
............................................................ugucauggaggcCcucucuuca...............  1     1  bc2
............................................................ugucauggaggGgcucucuuca...............  3     1  bc2
............................................................ugucauggaggcgcucuGuuca...............  1     1  bc2
............................................................Ggucauggaggcgcucucuuca...............  1     1  bc2
............................................................ugucauggaUgcgcucucuuca...............  1     1  bc2
............................................................ugucauggaggcgcucuUuuca...............  4     1  bc2
............................................................ugucGuggaggcgcucucuuca...............  1     1  bc2
............................................................ugucauggagAcgcucucuuca...............  9     1  bc2
............................................................ugucauggaggcgcucucuucU...............  455   1  bc2
............................................................ugucauggagCcgcucucuuca...............  17    1  bc2
............................................................uAucauggaggcgcucucuuca...............  1     1  bc2
............................................................ugucauggaggcgUucucuuca...............  1     1  bc2
............................................................ugucUuggaggcgcucucuuca...............  1     1  bc2
............................................................ugAcauggaggcgcucucuuca...............  1     1  bc2
............................................................ugucauggaggcgcucCcuuca...............  1     1  bc2
............................................................ugucauggaggcgcucucuAca...............  4     1  bc2
............................................................ugucauggaggcgcucucuGca...............  3     1  bc2
```

```
                  cel-miR-47*
                                                            cel-miR-47
gagagccgacugaaacugaagagagcagucuauugacagucgguuacucgaaaucuuuacugucauggaggcgcucucuucagaugaugucuggccc

............................................................uguUauggaggcgcucucuuca...............  2      1   bc2
............................................................ugucauggaggcgcucucuuca...............  3268   0   bc2
............................................................ugucauggaggcgcucucuuUa...............  1      1   bc2
............................................................ugucauggaggcgcuUucuuca...............  1      1   bc2
............................................................ugucauUgaggcgcucucuuca...............  1      1   bc2
............................................................ugGcauggaggcgcucucuuca...............  1      1   bc2
............................................................ugucauggGggcgcucucuuca...............  4      1   bc2
............................................................ugucauggUggcgcucucuuca...............  1      1   bc2
............................................................Cgucauggaggcgcucucuuca...............  1      1   bc2
............................................................ugucauggaggAgcucucuuca...............  2      1   bc2
............................................................ugucauggaggcUcucucuuca...............  3      1   bc2
............................................................uguAauggaggcgcucucuuca...............  1      1   bc2
............................................................ugucauggaggcgcucucuucG...............  6      1   bc2
............................................................ugucauggaggcgAucucuuca...............  2      1   bc2
............................................................ugucauggaggcgcucucuCca...............  3      1   bc2
............................................................ugucauggaggcgcucucAuca...............  1      1   bc2
............................................................ugucauggagUcgcucucuuca...............  9246   1   bc2
............................................................ugucauggaggcgcucucuuAa...............  2      1   bc2
............................................................ugucauggaggcAcucucuuca...............  1      1   bc2
............................................................ugucauAgaggcgcucucuuca...............  1      1   bc2
............................................................uguGauggaggcgcucucuuca...............  1      1   bc2
............................................................ugucCuggaggcgcucucuuca...............  1      1   bc2
............................................................ugucauggaggcgcucucuucC...............  6      1   bc2
............................................................ugucauggaggcgcucuAuuca...............  3      1   bc2
............................................................ugucauggaggcgcuAucuuca...............  2      1   bc2
............................................................ugucauggaggcgcucucuucaU..............  129    1   bc2
............................................................ugucauggaggcgcucucuucaA..............  7      1   bc2
............................................................ugucauggagUcgcucucuucag..............  1      1   bc2
.............................................................gucauggaggcgcucucuuca...............  10     0   bc2
.............................................................gCcauggaggcgcucucuuca...............  1      1   bc2
.............................................................gucauggagUcgcucucuuca...............  16     1   bc2
...............................................................cauggagUcgcucucuuca...............  1      1   bc2
................................................................auggagUcgcucucuuca...............  12     1   bc2
................................................................auggaggcgcucucuuca...............  3      0   bc2

.................Caagagagcagucuauugacagu.........................................................  1      1   bc7
..................aagagagcCgucuauugac............................................................  3      1   bc7
..................aagagagcagucuauugaca...........................................................  1      0   bc7
..................aagagagcCgucuauugacag..........................................................  226    1   bc7
..................aagagagcagucuauugacag..........................................................  122    0   bc7
..................aagagagcagucuauugacGg..........................................................  1      1   bc7
..................aagagagcagucuauuCacag..........................................................  1      1   bc7
..................aagagagcagucCauugacag..........................................................  1      1   bc7
..................aagagagcagucuauugaGagu.........................................................  2      1   bc7
..................aagagagcagCcuauugacagu.........................................................  2      1   bc7
..................aagagCgcagucuauugacagu.........................................................  2      1   bc7
..................aagagagcagucuauugacGgu.........................................................  2      1   bc7
..................aagagagcagucuauugacagu.........................................................  2338   0   bc7
..................aaAagagcagucuauugacagu.........................................................  2      1   bc7
..................Gagagagcagucuauugacagu.........................................................  2      1   bc7
..................aagagagcCgucuauugacagu.........................................................  3345   1   bc7
..................aagagagcagAcuauugacagu.........................................................  2      1   bc7
..................aagagagcaguGuauugacagu.........................................................  1      1   bc7
..................aagagagcagucCauugacagu.........................................................  1      1   bc7
..................aagagagcagucuauuGcagu..........................................................  2      1   bc7
..................aagagagcagucuauugUcagu.........................................................  1      1   bc7
..................aagagUgcagucuauugacagu.........................................................  2      1   bc7
..................aagagagcUgucuauugacagu.........................................................  2      1   bc7
..................aagagagcagucuGuugacagu.........................................................  1      1   bc7
..................aagagagcagucuauugacCgu.........................................................  3      1   bc7
..................aagagagcagucuauCgacagu.........................................................  1      1   bc7
..................aagagagcagucuUuugacagu.........................................................  1      1   bc7
..................aagagagcagucuauugaAagu.........................................................  2      1   bc7
..................aagagagcagucuauugacagG.........................................................  3      1   bc7
..................aagagGgcagucuauugacagu.........................................................  1      1   bc7
..................aagagagcaguUuauugacagu.........................................................  1      1   bc7
..................aagagagcagucuauugaUagu.........................................................  2      1   bc7
..................aagagagcGgucuauugacagu.........................................................  1      1   bc7
..................aagagagcagucuauugacaguc........................................................  1      0   bc7
```

cel-miR-47*

cel-miR-47

gagagccgacugaaacugaagagagcagucuauugacagucgguuacucgaaaucuuuacugucauggaggcgcucucuucagaugaugucuggccc

..................aagagagcagucuauugacaguA..	1	1	bc7
..................aagagagcagucuauugacaguU..	25	1	bc7
..................aagagagcagucuauugacaguUg...	1	1	bc7
...................agagagcCgucuauugacag..	4	1	bc7
...................agagagcCgucuauugacagu...	22	1	bc7
....................Aagagcagucuauugacagu...	1	1	bc7
.....................agagcCgucuauugacagu...	1	1	bc7
......................gagcagucuauugacagu...	11	0	bc7
......................gagcCgucuauugacagu...	14	1	bc7
....cgguuacCcgaaaucuuuac.....................................	1	1	bc7
....cgguuacucgaaaucuuuac.....................................	1	0	bc7
....cugucauggagUcgcucucuu.................	2	1	bc7
....cugucauggaggcgcucucuu.................	3	0	bc7
....cugucauggaggcgcucucuuca...............	3	0	bc7
....cugucauggaggcgcucucuucU...............	3	1	bc7
....cugucauggagUcgcucucuuca...............	19	1	bc7
....ugucauggagUcgcucuc...................	19	1	bc7
....ugucauggaggcgcucuc...................	14	0	bc7
....ugucauggaggcgcuUucu..................	1	1	bc7
....ugucauggaggcgcucucG..................	1	1	bc7
....ugucauggaggcgcCcucu..................	1	1	bc7
....ugucauggaggcgcucucA..................	1	1	bc7
....ugucauggaggcgcucCcu..................	1	1	bc7
....ugucauggaggcgcucucu..................	198	0	bc7
....ugucauggaggAgcucucu..................	1	1	bc7
....ugucauggagUcgcucucu..................	66	1	bc7
....ugucauggaggcgcucuUu..................	1	1	bc7
....uguGauggaggcgcucucuu.................	1	1	bc7
....ugucauggagAcgcucucuu.................	1	1	bc7
....ugucauggagUcgcucucuu.................	819	1	bc7
....ugucauggaggcgcuAucuu.................	1	1	bc7
....ugucauggaggcgcCcucuu.................	1	1	bc7
....ugucauggaggAgcucucuu.................	2	1	bc7
....ugucauggaggcgGucucuu.................	1	1	bc7
....ugucauggagCcgcucucuu.................	6	1	bc7
....ugucauggUggcgcucucuu.................	4	1	bc7
....uUucauggaggcgcucucuu.................	1	1	bc7
....ugucauggaggcgcucucuA.................	1	1	bc7
....ugucauggaggcCcucucuu.................	1	1	bc7
....ugucauggaggcgUucucuu.................	1	1	bc7
....ugucauggaggcgcucucuu.................	1378	0	bc7
....Cgucauggaggcgcucucuu.................	1	1	bc7
....ugGcauggaggcgcucucuu.................	2	1	bc7
....ugucauggaggGgcucucuu.................	1	1	bc7
....ugucaAggaggcgcucucuu.................	1	1	bc7
....ugucauggaggcgcucucuG.................	3	1	bc7
....ugucauggaggcgAucucuuc................	1	1	bc7
....ugucauggaggcgcucucuuc................	579	0	bc7
....ugGcauggaggcgcucucuuc................	1	1	bc7
....ugCcauggaggcgcucucuuc................	1	1	bc7
....ugucaugUaggcgcucucuuc................	1	1	bc7
....ugucauggUggcgcucucuuc................	1	1	bc7
....ugucaugAaggcgcucucuuc................	1	1	bc7
....ugucauggaggcgcucCcuuc................	1	1	bc7
....Agucauggaggcgcucucuuc................	1	1	bc7
....ugucGuggaggcgcucucuuc................	1	1	bc7
....ugucauggaggcgcuUucuuc................	1	1	bc7
....ugucauggaggcgcucucuuU................	10	1	bc7
....ugucauggagUcgcucucuuc................	349	1	bc7
....ugucauggaggcgcucAcuuc................	1	1	bc7
....ugucauggCggcgcucucuuc................	1	1	bc7
....ugucGuggaggcgcucucuuca...............	6	1	bc7
....ugucauggaggcgAucucuuca...............	3	1	bc7
....ugucauggaUgcgcucucuuca...............	1	1	bc7
....ugucauggaggGgcucucuuca...............	13	1	bc7
....ugucaCggaggcgcucucuuca...............	2	1	bc7
....ugucauggaggcUcucucuuca...............	3	1	bc7
....ugucauggaggcgcucucuucC...............	13	1	bc7
....ugucauggaggcgcuAucuuca...............	6	1	bc7

```
                  cel-miR-47*
                                                    cel-miR-47
gagagccgacugaaacugaagagagcagucuauugacagucgguuacucgaaaucuuuacugucauggaggcgcucucuucagaugaugucuggccc

............................................................ugucauggagCcgcucucuuca...............   49      1   bc7
............................................................ugucauggaggcgcucucuCca...............   22      1   bc7
............................................................uguAauggaggcgcucucuuca...............   1       1   bc7
............................................................ugGcauggaggcgcucucuuca...............   7       1   bc7
............................................................ugucauggaggcgcucGcuuca...............   1       1   bc7
............................................................ugucauggaggcgcucucCuca...............   1       1   bc7
............................................................ugucauggaggcgcuGucuuca...............   1       1   bc7
............................................................ugucauggaggcCcucucuuca...............   1       1   bc7
............................................................ugucauggaggcgcAcucuuca...............   1       1   bc7
............................................................ugucauggaggcgcucuUuuca...............   4       1   bc7
............................................................ugucauCgaggcgcucucuuca...............   1       1   bc7
............................................................ugCcauggaggcgcucucuuca...............   1       1   bc7
............................................................ugucauAgaggcgcucucuuca...............   1       1   bc7
............................................................ugucauggaggcgcucucuAca...............   5       1   bc7
............................................................uguUauggaggcgcucucuuca...............   3       1   bc7
............................................................ugucauggaggcgUucucuuca...............   2       1   bc7
............................................................ugucauggagUcgcucucuuca...............   19121   1   bc7
............................................................ugucauUgaggcgcucucuuca...............   1       1   bc7
............................................................ugucauggaggcgcucCcuuca...............   3       1   bc7
............................................................ugucauggaggcgcCcucuuca...............   2       1   bc7
............................................................ugucauggaggcgcucucuuAa...............   7       1   bc7
............................................................ugucauggaggcgcucucuucG...............   18      1   bc7
............................................................ugucaGggaggcgcucucuuca...............   1       1   bc7
............................................................ugucaugAaggcgcucucuuca...............   2       1   bc7
............................................................ugucauggaggcgcuUucuuca...............   1       1   bc7
............................................................ugucauggaCgcgcucucuuca...............   1       1   bc7
............................................................ugucauggaggcgcucucuuca...............   6267    0   bc7
............................................................ugucauggagAcgcucucuuca...............   16      1   bc7
............................................................ugucauggaggcgcucucuucU...............   1349    1   bc7
............................................................ugucauggaggcgcucucuuGa...............   2       1   bc7
............................................................ugucauggaggcAcucucuuca...............   4       1   bc7
............................................................uAucauggaggcgcucucuuca...............   2       1   bc7
............................................................ugucauggGggcgcucucuuca...............   6       1   bc7
............................................................ugucauggaggUgcucucuuca...............   3       1   bc7
............................................................ugucauggaggcgcucucuuUa...............   2       1   bc7
............................................................Cgucauggaggcgcucucuuca...............   5       1   bc7
............................................................ugucauggaggcgcucuAuuca...............   2       1   bc7
............................................................ugucauggaggAgcucucuuca...............   4       1   bc7
............................................................ugucCuggaggcgcucucuuca...............   1       1   bc7
............................................................Ggucauggaggcgcucucuuca...............   4       1   bc7
............................................................ugucaugCaggcgcucucuuca...............   2       1   bc7
............................................................ugucauggCggcgcucucuuca...............   1       1   bc7
............................................................ugucauggUggcgcucucuuca...............   10      1   bc7
............................................................ugucauggagUcgcucucuucag..............   1       1   bc7
............................................................ugucauggaggcgcucucuucag..............   2       0   bc7
............................................................ugucauggaggcgcucucuucaU..............   364     1   bc7
............................................................ugucauggaggcgcucucuucaA..............   13      1   bc7
............................................................ugucauggaggcgcucucuucaAa.............   2       1   bc7
............................................................ugucauggaggcgcucucuucaCa.............   1       1   bc7
.............................................................gucauggaggcgcucucuuc.................   1       0   bc7
.............................................................gucauggagUcgcucucuuca...............   27      1   bc7
.............................................................gucauggaggcgcucucuucU...............   4       1   bc7
.............................................................gucauggaggcgcucucuCca...............   1       1   bc7
.............................................................gucauggaggcgcucucuuca...............   16      0   bc7
..............................................................ucauggaggcgcucucuuca...............   1       0   bc7
..............................................................ucauggagUcgcucucuuca...............   1       1   bc7
..............................................................ucauggagAcgcucucuuca...............   1       1   bc7
...............................................................cauggaggcgcucucuuca...............   1       0   bc7
...............................................................cauggagUcgcucucuuca...............   1       1   bc7
................................................................auggagUcgcucucuuca...............   19      1   bc7
................................................................auggaggcgcucucuuca...............   15      0   bc7
```
