# Supplementary figures and images for: Reliable reference miRNAs for quantitative gene expression analysis of stress responses in Caenorhabditis elegans
Source: BMC Genomics. 2014 Mar 21;15:222. doi: 10.1186/1471-2164-15-222 (PMC3997968; doi:10.1186/1471-2164-15-222)

## All conditions

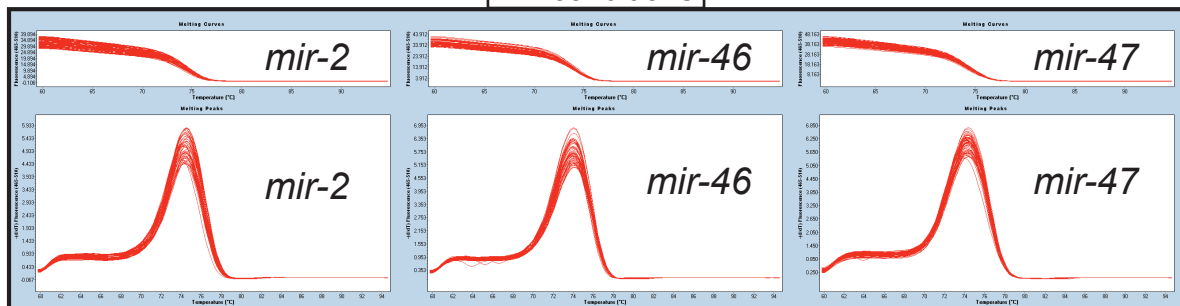

## UV

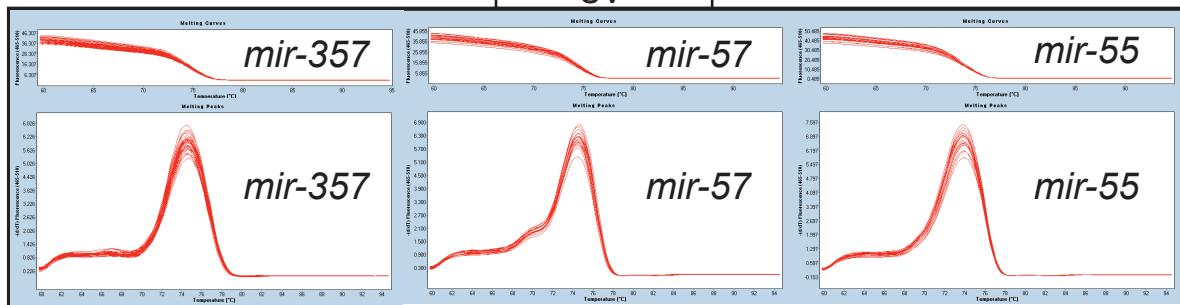

## Heat stress

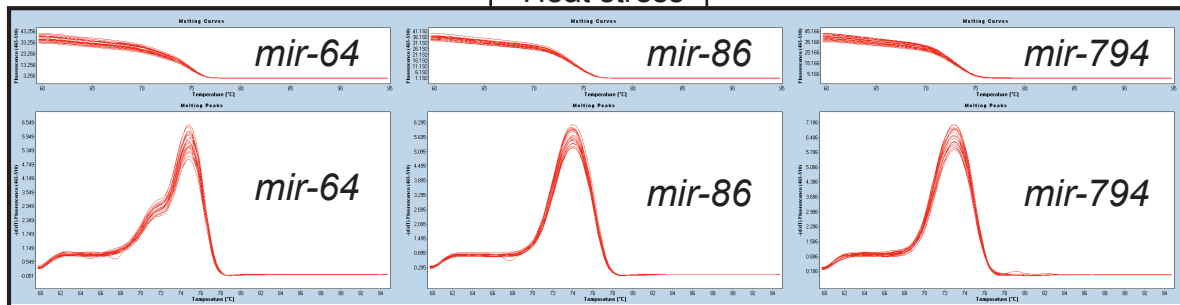

## Hypoxia N2+hif-1

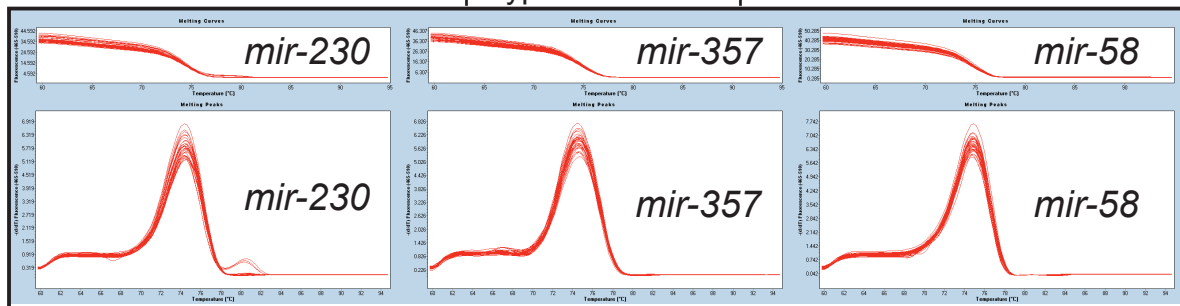

## Hypoxia N2

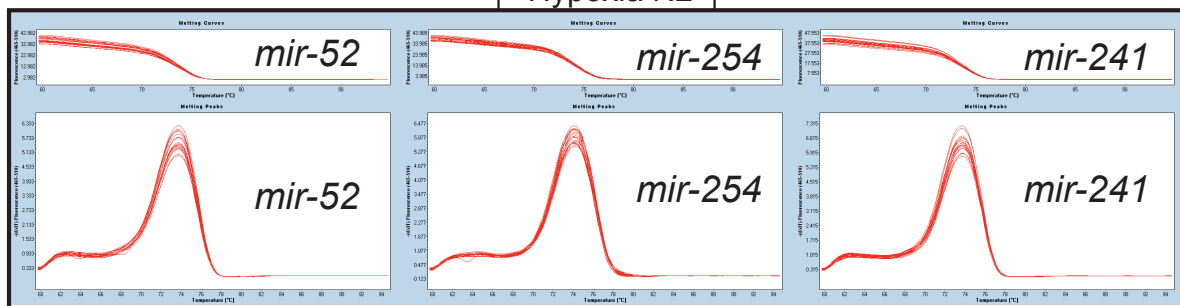

Supplement: Additional file 7: Figure S4 — Melting curves (smaller graphs on the top) and melting peaks (bigger graphs at the bottom) of the primers used to validate selected miRNAs by qPCR. All pairs of primers show harmonious curves and single peaks, which indicate specificity in amplifying one product only. [file 1471-2164-15-222-S7.pdf]
