# Supplementary material for: Reliable reference miRNAs for quantitative gene expression analysis of stress responses in Caenorhabditis elegans
Source: BMC Genomics. 2014 Mar 21;15:222. doi: 10.1186/1471-2164-15-222 (PMC3997968; doi:10.1186/1471-2164-15-222)
Supplement: Additional file 8: Figure S5 — RNA quality and integrity assessed by Nanodrop and Bioanalyzer. Bioanalyzer image for all samples (numbering of the samples as in b) where high integrity of the RNA samples is observed. bp = base pairs. b. 260/280 and 260/230 ratios obtained by the Nanodrop as well as RIN (RNA Integrity Number) measurements obtained by the Bioanalyzer for all the samples. [file 1471-2164-15-222-S8.pdf]

a

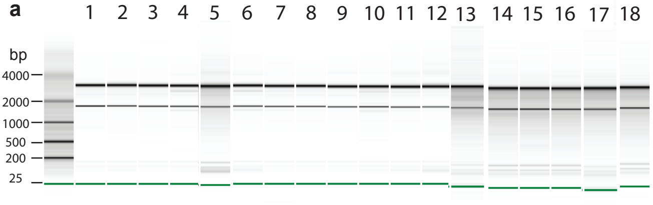

b

	Sample type	260/280	260/230	RIN
1	UV sample 1	2.2	2.51	9.6
2	UV sample 2	2.19	2.48	9.7
3	UV sample 3	2.21	2.43	9.7
4	Temp. sample 1	2.21	2.45	9.6
5	Temp. sample 2	2.06	2.25	9.6
6	Temp. sample 3	2.17	2.02	9.8
7	N2 Hypoxia sample 1	2.19	2.43	9.9
8	N2 Hypoxia sample 2	2.20	2.47	10
9	N2 Hypoxia sample 3	2.19	2.43	9.2
10	N2 control sample 1	2.19	2.45	9.2
11	N2 control sample 2	2.17	2.36	9.9
12	N2 control sample 3	2.14	2.24	10
13	hif-1 Hypoxia sample 1	2.20	2.52	7.2
14	hif-1 Hypoxia sample 2	2.21	2.28	7.1
15	hif-1 Hypoxia sample 3	2.21	2.51	7.2
16	hif-1 Hypoxia control sample 1	2.21	2.42	7.2
17	hif-1 Hypoxia control sample 2	2.22	2.40	7.2
18	hif-1 Hypoxia control sample 3	2.20	2.49	7.5
